# Supplementary material for: Identifying psychiatrist characteristics associated with likelihood of recommending involuntary hospitalization for patients using a novel tool to assess decision-making
Source: PLOS Ment Health. 2026 Apr 20;3(4):e0000598. doi: 10.1371/journal.pmen.0000598 (PMC13095014; doi:10.1371/journal.pmen.0000598)
Supplement: S1 File — The supplemental S1 File contains summaries of aggregated data, output from all statistical analyses reported in the paper, and the underlying statistical code from SAS. (PDF) [file pmen.0000598.s001.pdf]

Lavie National survey
Descriptive Information
Missing data in main variables

The FREQ Procedure

missc5	Frequency	Percent	Cumulative Frequency	Cumulative Percent
0	187	76.02	187	76.02
1	26	10.57	213	86.59
2	10	4.07	223	90.65
3	7	2.85	230	93.50
4	5	2.03	235	95.53
5	11	4.47	246	100.00

missdemos	Frequency	Percent	Cumulative Frequency	Cumulative Percent
0	229	93.09	229	93.09
1	15	6.10	244	99.19
2	1	0.41	245	99.59
4	1	0.41	246	100.00

Lavie National survey
Descriptive Information
Demographic variable frequencies

The FREQ Procedure

man, woman, QU, NB				
**gender**	**Frequency**	**Percent**	**Cumulative Frequency**	**Cumulative Percent**
**0_0_0_0**	3	1.22	3	1.22
**0_0_1_0**	1	0.41	4	1.63
**0_1_0_0**	128	52.03	132	53.66
**0_1_0_1**	1	0.41	133	54.07
**1_0_0_0**	112	45.53	245	99.59
**1_0_1_0**	1	0.41	246	100.00

malefemale	Frequency	Percent	Cumulative Frequency	Cumulative Percent
**.**	6	2.44	6	2.44
**0**	128	52.03	134	54.47
**1**	112	45.53	246	100.00

White				
**White**	**Frequency**	**Percent**	**Cumulative Frequency**	**Cumulative Percent**
**0**	102	41.46	102	41.46
**1**	144	58.54	246	100.00

Black				
**Black**	**Frequency**	**Percent**	**Cumulative Frequency**	**Cumulative Percent**
**0**	238	96.75	238	96.75
**1**	8	3.25	246	100.00

SAsian				
**SAsian**	**Frequency**	**Percent**	**Cumulative Frequency**	**Cumulative Percent**
**0**	213	86.59	213	86.59
**1**	33	13.41	246	100.00

EAsian				
**EAsian**	**Frequency**	**Percent**	**Cumulative Frequency**	**Cumulative Percent**
**0**	211	85.77	211	85.77
**1**	35	14.23	246	100.00

Lavie National survey
Descriptive Information
Demographic variable frequencies

The FREQ Procedure

MENA				
MENA	Frequency	Percent	Cumulative Frequency	Cumulative Percent
0	241	97.97	241	97.97
1	5	2.03	246	100.00

Orace				
Orace	Frequency	Percent	Cumulative Frequency	Cumulative Percent
0	227	92.28	227	92.28
1	19	7.72	246	100.00

Skip_race				
Skip_race	Frequency	Percent	Cumulative Frequency	Cumulative Percent
0	238	96.75	238	96.75
1	8	3.25	246	100.00

N_Race				
N_Race	Frequency	Percent	Cumulative Frequency	Cumulative Percent
0	3	1.22	3	1.22
1	234	95.12	237	96.34
2	9	3.66	246	100.00

Latino				
Latino	Frequency	Percent	Cumulative Frequency	Cumulative Percent
.	3	1.22	3	1.22
0	206	83.74	209	84.96
1	35	14.23	244	99.19
99	2	0.81	246	100.00

minority	Frequency	Percent	Cumulative Frequency	Cumulative Percent
.	11	4.47	11	4.47
0	113	45.93	124	50.41
1	122	49.59	246	100.00

Lavie National survey
Descriptive Information
Demographic variable frequencies

The FREQ Procedure

agecat3	Frequency	Percent	Cumulative Frequency	Cumulative Percent
.	1	0.41	1	0.41
1	76	30.89	77	31.30
2	111	45.12	188	76.42
3	58	23.58	246	100.00

status				
status	Frequency	Percent	Cumulative Frequency	Cumulative Percent
.	3	1.22	3	1.22
0	96	39.02	99	40.24
1	135	54.88	234	95.12
2	12	4.88	246	100.00

status2	Frequency	Percent	Cumulative Frequency	Cumulative Percent
.	3	1.22	3	1.22
0	96	39.02	99	40.24
1	147	59.76	246	100.00

Site_region				
Site_region	Frequency	Percent	Cumulative Frequency	Cumulative Percent
1	40	16.26	40	16.26
2	18	7.32	58	23.58
3	34	13.82	92	37.40
4	98	39.84	190	77.24
5	56	22.76	246	100.00

Lavie National survey
Descriptive Information
Demographic variable frequencies

The FREQ Procedure

Frequency<br>Percent<br>Row Pct<br>Col Pct

Table of malefemale by gender

	gender(man, woman, QU, NB)						
malefemale	0_0_0_0	0_0_1_0	0_1_0_0	0_1_0_1	1_0_0_0	1_0_1_0	Total
.	3<br>1.22<br>50.00<br>100.00	1<br>0.41<br>16.67<br>100.00	0<br>0.00<br>0.00<br>0.00	1<br>0.41<br>16.67<br>100.00	0<br>0.00<br>0.00<br>0.00	1<br>0.41<br>16.67<br>100.00	6<br>2.44
0	0<br>0.00<br>0.00<br>0.00	0<br>0.00<br>0.00<br>0.00	128<br>52.03<br>100.00<br>100.00	0<br>0.00<br>0.00<br>0.00	0<br>0.00<br>0.00<br>0.00	0<br>0.00<br>0.00<br>0.00	128<br>52.03
1	0<br>0.00<br>0.00<br>0.00	0<br>0.00<br>0.00<br>0.00	0<br>0.00<br>0.00<br>0.00	0<br>0.00<br>0.00<br>0.00	112<br>45.53<br>100.00<br>100.00	0<br>0.00<br>0.00<br>0.00	112<br>45.53
Total	3<br>1.22	1<br>0.41	128<br>52.03	1<br>0.41	112<br>45.53	1<br>0.41	246<br>100.00

Lavie National survey
Descriptive Information
Demographic variable frequencies

The FREQ Procedure

Frequency<br>Percent<br>Row Pct<br>Col Pct

Table of races by minority

races(White, Black, SAsian, EAsian, MENA, ORace, skip_race)	minority			
	.	0	1	Total
**0_0_0_0_0_0_0**	3<br>1.22<br>100.00<br>27.27	0<br>0.00<br>0.00<br>0.00	0<br>0.00<br>0.00<br>0.00	3<br>1.22
**0_0_0_0_0_0_1**	8<br>3.25<br>100.00<br>72.73	0<br>0.00<br>0.00<br>0.00	0<br>0.00<br>0.00<br>0.00	8<br>3.25
**0_0_0_0_0_1_0**	0<br>0.00<br>0.00<br>0.00	0<br>0.00<br>0.00<br>0.00	19<br>7.72<br>100.00<br>15.57	19<br>7.72
**0_0_0_0_1_0_0**	0<br>0.00<br>0.00<br>0.00	0<br>0.00<br>0.00<br>0.00	2<br>0.81<br>100.00<br>1.64	2<br>0.81
**0_0_0_1_0_0_0**	0<br>0.00<br>0.00<br>0.00	0<br>0.00<br>0.00<br>0.00	31<br>12.60<br>100.00<br>25.41	31<br>12.60
**0_0_1_0_0_0_0**	0<br>0.00<br>0.00<br>0.00	0<br>0.00<br>0.00<br>0.00	30<br>12.20<br>100.00<br>24.59	30<br>12.20
**0_0_1_1_0_0_0**	0<br>0.00<br>0.00<br>0.00	0<br>0.00<br>0.00<br>0.00	1<br>0.41<br>100.00<br>0.82	1<br>0.41
**0_1_0_0_0_0_0**	0<br>0.00<br>0.00<br>0.00	0<br>0.00<br>0.00<br>0.00	7<br>2.85<br>100.00<br>5.74	7<br>2.85
**0_1_0_0_1_0_0**	0<br>0.00<br>0.00<br>0.00	0<br>0.00<br>0.00<br>0.00	1<br>0.41<br>100.00<br>0.82	1<br>0.41
**1_0_0_0_0_0_0**	0<br>0.00<br>0.00<br>0.00	113<br>45.93<br>82.48<br>100.00	24<br>9.76<br>17.52<br>19.67	137<br>55.69
**1_0_0_0_1_0_0**	0<br>0.00<br>0.00<br>0.00	0<br>0.00<br>0.00<br>0.00	2<br>0.81<br>100.00<br>1.64	2<br>0.81
**1_0_0_1_0_0_0**	0<br>0.00<br>0.00<br>0.00	0<br>0.00<br>0.00<br>0.00	3<br>1.22<br>100.00<br>2.46	3<br>1.22

Lavie National survey
Descriptive Information
Demographic variable frequencies

The FREQ Procedure

Frequency<br>Percent<br>Row Pct<br>Col Pct

Table of races by minority				
	minority			
races(White, Black, SAsian, EAsian, MENA, ORace, skip_race)	.	0	1	Total
1_0_1_0_0_0_0	0<br>0.00<br>0.00<br>0.00	0<br>0.00<br>0.00<br>0.00	2<br>0.81<br>100.00<br>1.64	2<br>0.81
Total	11<br>4.47	113<br>45.93	122<br>49.59	246<br>100.00

Lavie National survey
Descriptive Information
Demographic variable frequencies

The FREQ Procedure

**Frequency**<br>**Percent**<br>**Row Pct**<br>**Col Pct**

Table of focus1_age by agecat3					
	agecat3				
focus1_age(focus1_age)	.	1	2	3	Total
.	1<br>0.41<br>100.00<br>100.00	0<br>0.00<br>0.00<br>0.00	0<br>0.00<br>0.00<br>0.00	0<br>0.00<br>0.00<br>0.00	1<br>0.41
1	0<br>0.00<br>0.00<br>0.00	1<br>0.41<br>100.00<br>1.32	0<br>0.00<br>0.00<br>0.00	0<br>0.00<br>0.00<br>0.00	1<br>0.41
2	0<br>0.00<br>0.00<br>0.00	75<br>30.49<br>100.00<br>98.68	0<br>0.00<br>0.00<br>0.00	0<br>0.00<br>0.00<br>0.00	75<br>30.49
3	0<br>0.00<br>0.00<br>0.00	0<br>0.00<br>0.00<br>0.00	111<br>45.12<br>100.00<br>100.00	0<br>0.00<br>0.00<br>0.00	111<br>45.12
4	0<br>0.00<br>0.00<br>0.00	0<br>0.00<br>0.00<br>0.00	0<br>0.00<br>0.00<br>0.00	36<br>14.63<br>100.00<br>62.07	36<br>14.63
5	0<br>0.00<br>0.00<br>0.00	0<br>0.00<br>0.00<br>0.00	0<br>0.00<br>0.00<br>0.00	13<br>5.28<br>100.00<br>22.41	13<br>5.28
6	0<br>0.00<br>0.00<br>0.00	0<br>0.00<br>0.00<br>0.00	0<br>0.00<br>0.00<br>0.00	7<br>2.85<br>100.00<br>12.07	7<br>2.85
7	0<br>0.00<br>0.00<br>0.00	0<br>0.00<br>0.00<br>0.00	0<br>0.00<br>0.00<br>0.00	2<br>0.81<br>100.00<br>3.45	2<br>0.81
Total	1<br>0.41	76<br>30.89	111<br>45.12	58<br>23.58	246<br>100.00

Lavie National survey
Descriptive Information
Ns and Means of main variables

The MEANS Procedure

Variable	Label	N	Mean	Std Dev	Minimum	Maximum
admit5		246	0.4756098	0.2457281	0	1.0000000
avgabscon5		223	68.0367713	14.2118303	21.0000000	100.0000000
SiteNo	SiteNo	246	5.0772358	2.5551329	1.0000000	8.0000000
malefemale		240	0.4666667	0.4999303	0	1.0000000
agecat3		245	1.9265306	0.7374011	1.0000000	3.0000000
minority		235	0.5191489	0.5006996	0	1.0000000
paternalism		240	2.6908333	0.6689304	1.0000000	4.4000000
worry_px	worry_px	240	2.1416667	0.5059617	1.0000000	4.0000000
use_laws	use_laws	241	3.0871369	0.8345910	1.0000000	4.0000000
risk_comfort	risk_comfort	241	2.9543568	0.6208124	1.0000000	4.0000000
ih_benefits	ih_benefits	241	2.5684647	0.5813789	2.0000000	4.0000000
perception_self_admit	perception_self_admit	241	2.0207469	0.5733537	1.0000000	3.0000000
perception_inst_admit	perception_inst_admit	240	1.8416667	0.6270851	1.0000000	3.0000000

Lavie National survey
Descriptive Information
distribution of admit decisions

The FREQ Procedure

admit5	Frequency	Percent	Cumulative Frequency	Cumulative Percent
**0**	13	5.28	13	5.28
**0.2**	55	22.36	68	27.64
**0.4**	57	23.17	125	50.81
**0.6**	77	31.30	202	82.11
**0.8**	35	14.23	237	96.34
**1**	9	3.66	246	100.00

Lavie National survey
Descriptive Information
correlations between admit rates, confidence in admit, and absolute level of confidence

The CORR Procedure

**3 Variables:**	admit5 avgreccon5 avgabscon5

Simple Statistics						
**Variable**	**N**	**Mean**	**Std Dev**	**Sum**	**Minimum**	**Maximum**
**admit5**	246	0.47561	0.24573	117.00000	0	1.00000
**avgreccon5**	223	-0.32556	36.58902	-72.60000	-100.00000	97.00000
**avgabscon5**	223	68.03677	14.21183	15172	21.00000	100.00000

Pearson Correlation Coefficients<br>Prob > |r| under H0: Rho=0<br>Number of Observations			
	**admit5**	**avgreccon5**	**avgabscon5**
**admit5**	1.00000<br><br>246	0.96648<br><.0001<br>223	0.09955<br>0.1384<br>223
**avgreccon5**	0.96648<br><.0001<br>223	1.00000<br><br>223	0.05890<br>0.3813<br>223
**avgabscon5**	0.09955<br>0.1384<br>223	0.05890<br>0.3813<br>223	1.00000<br><br>223

Lavie National survey
descriptive data for attendings only

The FREQ Procedure

yearspracticing				
**yearspracticing**	**Frequency**	**Percent**	**Cumulative Frequency**	**Cumulative Percent**
**1**	37	38.54	37	38.54
**2**	22	22.92	59	61.46
**3**	11	11.46	70	72.92
**4**	12	12.50	82	85.42
**5**	7	7.29	89	92.71
**6**	2	2.08	91	94.79
**7**	3	3.13	94	97.92
**9**	2	2.08	96	100.00

training_pes				
**training_pes**	**Frequency**	**Percent**	**Cumulative Frequency**	**Cumulative Percent**
**0**	42	43.75	42	43.75
**1**	54	56.25	96	100.00

percent_er				
**percent_er**	**Frequency**	**Percent**	**Cumulative Frequency**	**Cumulative Percent**
**1**	50	52.08	50	52.08
**2**	20	20.83	70	72.92
**3**	7	7.29	77	80.21
**4**	7	7.29	84	87.50
**5**	3	3.13	87	90.63
**6**	9	9.38	96	100.00

er_exp	Frequency	Percent	Cumulative Frequency	Cumulative Percent
**0**	50	52.08	50	52.08
**1**	46	47.92	96	100.00

Lavie National survey
descriptive data for attendings only
settings

The FREQ Procedure

EPS				
EPS	Frequency	Percent	Cumulative Frequency	Cumulative Percent
0	65	67.71	65	67.71
1	31	32.29	96	100.00

outpx	Frequency	Percent	Cumulative Frequency	Cumulative Percent
0	47	48.96	47	48.96
1	49	51.04	96	100.00

Private				
Private	Frequency	Percent	Cumulative Frequency	Cumulative Percent
0	80	83.33	80	83.33
1	16	16.67	96	100.00

AMC_opx				
AMC_opx	Frequency	Percent	Cumulative Frequency	Cumulative Percent
0	65	67.71	65	67.71
1	31	32.29	96	100.00

Comm_opx				
Comm_opx	Frequency	Percent	Cumulative Frequency	Cumulative Percent
0	91	94.79	91	94.79
1	5	5.21	96	100.00

PHP				
PHP	Frequency	Percent	Cumulative Frequency	Cumulative Percent
0	95	98.96	95	98.96
1	1	1.04	96	100.00

Lavie National survey
descriptive data for attendings only
settings

The FREQ Procedure

Frequency
Percent
Row Pct
Col Pct

Table of SUD_opx by SUD_ipx			
	SUD_ipx(SUD_ipx)		
SUD_opx(SUD_opx)	0	1	Total
0	92<br>95.83<br>98.92<br>96.84	1<br>1.04<br>1.08<br>100.00	93<br>96.88
1	3<br>3.13<br>100.00<br>3.16	0<br>0.00<br>0.00<br>0.00	3<br>3.13
Total	95<br>98.96	1<br>1.04	96<br>100.00

inpx2	Frequency	Percent	Cumulative Frequency	Cumulative Percent
0	51	53.13	51	53.13
1	45	46.88	96	100.00

AMC_ipx				
AMC_ipx	Frequency	Percent	Cumulative Frequency	Cumulative Percent
0	57	59.38	57	59.38
1	39	40.63	96	100.00

Comm_ipx				
Comm_ipx	Frequency	Percent	Cumulative Frequency	Cumulative Percent
0	94	97.92	94	97.92
1	2	2.08	96	100.00

State_H				
State_H	Frequency	Percent	Cumulative Frequency	Cumulative Percent
0	95	98.96	95	98.96
1	1	1.04	96	100.00

CL				
CL	Frequency	Percent	Cumulative Frequency	Cumulative Percent
0	86	89.58	86	89.58
1	10	10.42	96	100.00

Lavie National survey
descriptive data for attendings only
settings

The FREQ Procedure

ukpx	Frequency	Percent	Cumulative Frequency	Cumulative Percent
0	82	85.42	82	85.42
1	14	14.58	96	100.00

Correctional				
Correctional	Frequency	Percent	Cumulative Frequency	Cumulative Percent
0	95	98.96	95	98.96
1	1	1.04	96	100.00

VA_H				
VA_H	Frequency	Percent	Cumulative Frequency	Cumulative Percent
0	88	91.67	88	91.67
1	8	8.33	96	100.00

County_H				
County_H	Frequency	Percent	Cumulative Frequency	Cumulative Percent
0	91	94.79	91	94.79
1	5	5.21	96	100.00

Lavie National survey
descriptive data for attendings only
settings

The FREQ Procedure

N_Settings				
N_Settings	Frequency	Percent	Cumulative Frequency	Cumulative Percent
1	52	54.17	52	54.17
2	29	30.21	81	84.38
3	12	12.50	93	96.88
4	2	2.08	95	98.96
6	1	1.04	96	100.00

outpx__pes_inpxNOpes				
settings2	Frequency	Percent	Cumulative Frequency	Cumulative Percent
0_0_0	9	9.38	9	9.38
0_0_1	20	20.83	29	30.21
0_1_0	9	9.38	38	39.58
0_1_1	9	9.38	47	48.96
1_0_0	25	26.04	72	75.00
1_0_1	11	11.46	83	86.46
1_1_0	8	8.33	91	94.79
1_1_1	5	5.21	96	100.00

inpx2	Frequency	Percent	Cumulative Frequency	Cumulative Percent
0	51	53.13	51	53.13
1	45	46.88	96	100.00

outpx	Frequency	Percent	Cumulative Frequency	Cumulative Percent
0	47	48.96	47	48.96
1	49	51.04	96	100.00

EPS				
EPS	Frequency	Percent	Cumulative Frequency	Cumulative Percent
0	65	67.71	65	67.71
1	31	32.29	96	100.00

Lavie National survey
descriptive data for attendings only
settings

The FREQ Procedure

eps__percent_er_pes				
**esettings**	**Frequency**	**Percent**	**Cumulative Frequency**	**Cumulative Percent**
**0_1_.**	50	52.08	50	52.08
**0_2_.**	1	1.04	51	53.13
**0_2_0**	7	7.29	58	60.42
**0_2_1**	4	4.17	62	64.58
**0_3_0**	1	1.04	63	65.63
**0_3_1**	1	1.04	64	66.67
**0_4_0**	1	1.04	65	67.71
**1_2_0**	2	2.08	67	69.79
**1_2_1**	6	6.25	73	76.04
**1_3_0**	3	3.13	76	79.17
**1_3_1**	2	2.08	78	81.25
**1_4_.**	1	1.04	79	82.29
**1_4_0**	3	3.13	82	85.42
**1_4_1**	2	2.08	84	87.50
**1_5_0**	2	2.08	86	89.58
**1_5_1**	1	1.04	87	90.63
**1_6_0**	2	2.08	89	92.71
**1_6_1**	7	7.29	96	100.00

Lavie National survey
descriptive data for attendings only
settings

The FREQ Procedure

Frequency<br>Percent<br>Row Pct<br>Col Pct

Table of pes by er_exp			
	er_exp		
pes(pes)	0	1	Total
0	0<br>0.00<br>0.00<br>.	21<br>47.73<br>100.00<br>47.73	21<br>47.73
1	0<br>0.00<br>0.00<br>.	23<br>52.27<br>100.00<br>52.27	23<br>52.27
Total	0<br>0.00	44<br>100.00	44<br>100.00
Frequency Missing = 52			

Lavie National survey
descriptive data for attendings only
settings

The CORR Procedure

4 Variables:	inpx2 outpx pes er_exp

Simple Statistics							
**Variable**	**N**	**Mean**	**Std Dev**	**Sum**	**Minimum**	**Maximum**	**Label**
**inpx2**	96	0.46875	0.50164	45.00000	0	1.00000	
**outpx**	96	0.51042	0.50252	49.00000	0	1.00000	
**pes**	44	0.52273	0.50526	23.00000	0	1.00000	pes
**er_exp**	96	0.47917	0.50219	46.00000	0	1.00000	

Pearson Correlation Coefficients<br>Prob > |r| under H0: Rho=0<br>Number of Observations				
	**inpx2**	**outpx**	**pes**	**er_exp**
**inpx2**	1.00000<br><br>96	-0.29100<br>0.0040<br>96	-0.31550<br>0.0370<br>44	-0.10707<br>0.2991<br>96
**outpx**	-0.29100<br>0.0040<br>96	1.00000<br><br>96	-0.13650<br>0.3769<br>44	-0.06170<br>0.5504<br>96
**pes**<br>**pes**	-0.31550<br>0.0370<br>44	-0.13650<br>0.3769<br>44	1.00000<br><br>44	.<br>.<br>44
**er_exp**	-0.10707<br>0.2991<br>96	-0.06170<br>0.5504<br>96	.<br>.<br>44	1.00000<br><br>96

Lavie National survey
descriptive data for trainees only

The FREQ Procedure

PGY				
**PGY**	**Frequency**	**Percent**	**Cumulative Frequency**	**Cumulative Percent**
**1**	35	23.81	35	23.81
**2**	45	30.61	80	54.42
**3**	31	21.09	111	75.51
**4**	28	19.05	139	94.56
**5**	8	5.44	147	100.00

Lavie National survey
Significance testing for admit likelihood

The TTEST Procedure

Variable: admit5

malefemale	Method	N	Mean	Std Dev	Std Err	Minimum	Maximum
**0**		128	0.4656	0.2435	0.0215	0	1.0000
**1**		112	0.4893	0.2414	0.0228	0	1.0000
**Diff (1-2)**	**Pooled**		-0.0237	0.2425	0.0314		
**Diff (1-2)**	**Satterthwaite**		-0.0237		0.0314		

malefemale	Method	Mean	95% CL Mean		Std Dev	95% CL Std Dev	
**0**		0.4656	0.4230	0.5082	0.2435	0.2169	0.2776
**1**		0.4893	0.4441	0.5345	0.2414	0.2134	0.2779
**Diff (1-2)**	**Pooled**	-0.0237	-0.0855	0.0382	0.2425	0.2225	0.2664
**Diff (1-2)**	**Satterthwaite**	-0.0237	-0.0854	0.0381			

Method	Variances	DF	t Value	Pr > |t|
**Pooled**	Equal	238	-0.75	0.4515
**Satterthwaite**	Unequal	234.31	-0.75	0.4513

Equality of Variances				
**Method**	**Num DF**	**Den DF**	**F Value**	**Pr > F**
**Folded F**	127	111	1.02	0.9283

Lavie National survey
Significance testing for admit likelihood

The TTEST Procedure

Variable: admit5

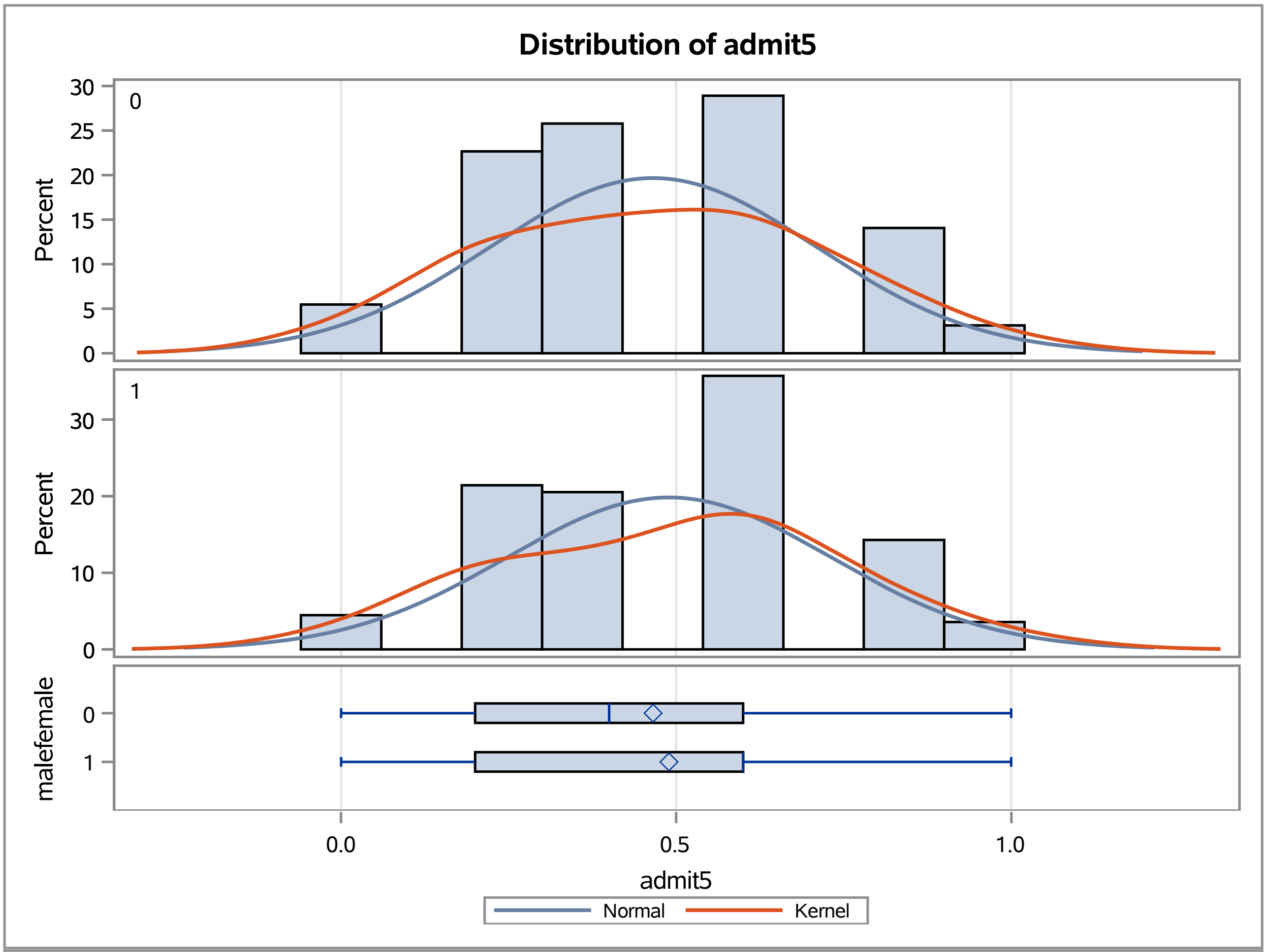

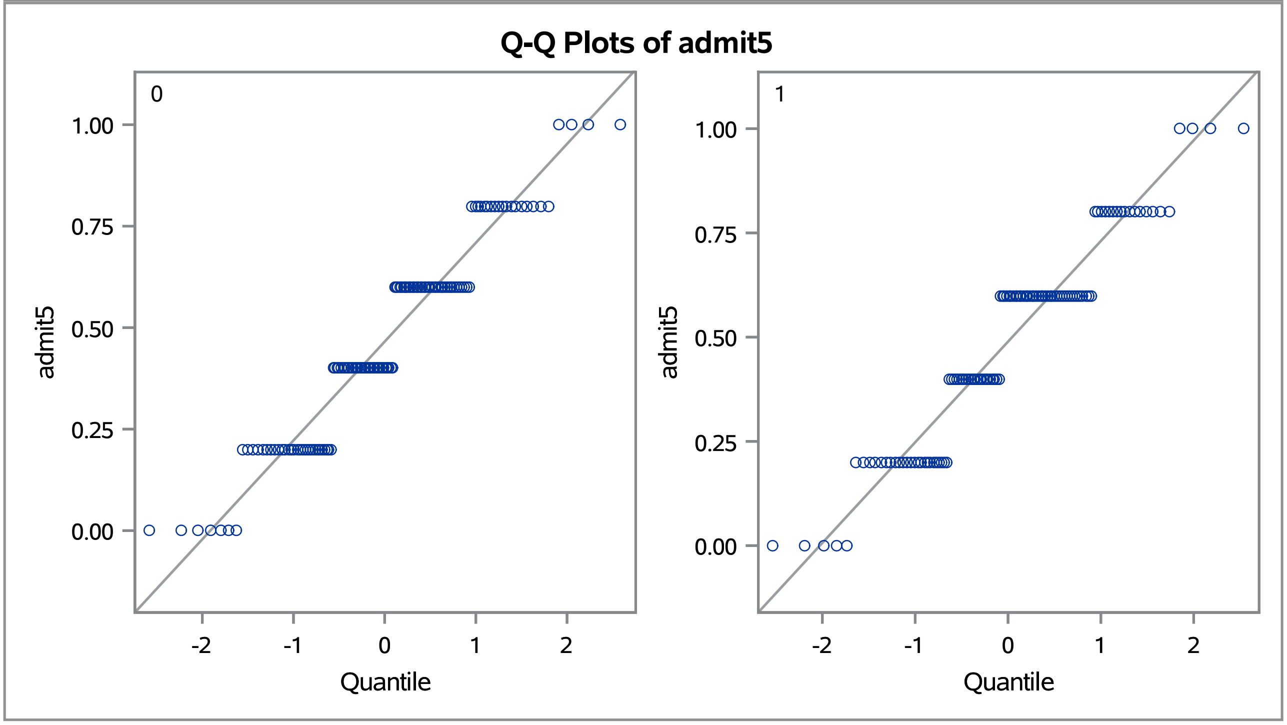

Lavie National survey
Significance testing for admit likelihood

The TTEST Procedure

Variable: admit5

minority	Method	N	Mean	Std Dev	Std Err	Minimum	Maximum
0		113	0.4584	0.2325	0.0219	0	1.0000
1		122	0.4902	0.2534	0.0229	0	1.0000
Diff (1-2)	Pooled		-0.0318	0.2436	0.0318		
Diff (1-2)	Satterthwaite		-0.0318		0.0317		

minority	Method	Mean	95% CL Mean		Std Dev	95% CL Std Dev	
0		0.4584	0.4151	0.5017	0.2325	0.2056	0.2675
1		0.4902	0.4447	0.5356	0.2534	0.2251	0.2899
Diff (1-2)	Pooled	-0.0318	-0.0944	0.0309	0.2436	0.2233	0.2679
Diff (1-2)	Satterthwaite	-0.0318	-0.0942	0.0307			

Method	Variances	DF	t Value	Pr > |t|
Pooled	Equal	233	-1.00	0.3190
Satterthwaite	Unequal	232.98	-1.00	0.3174

Equality of Variances				
Method	Num DF	Den DF	F Value	Pr > F
Folded F	121	112	1.19	0.3569

Lavie National survey
Significance testing for admit likelihood

The TTEST Procedure

Variable: admit5

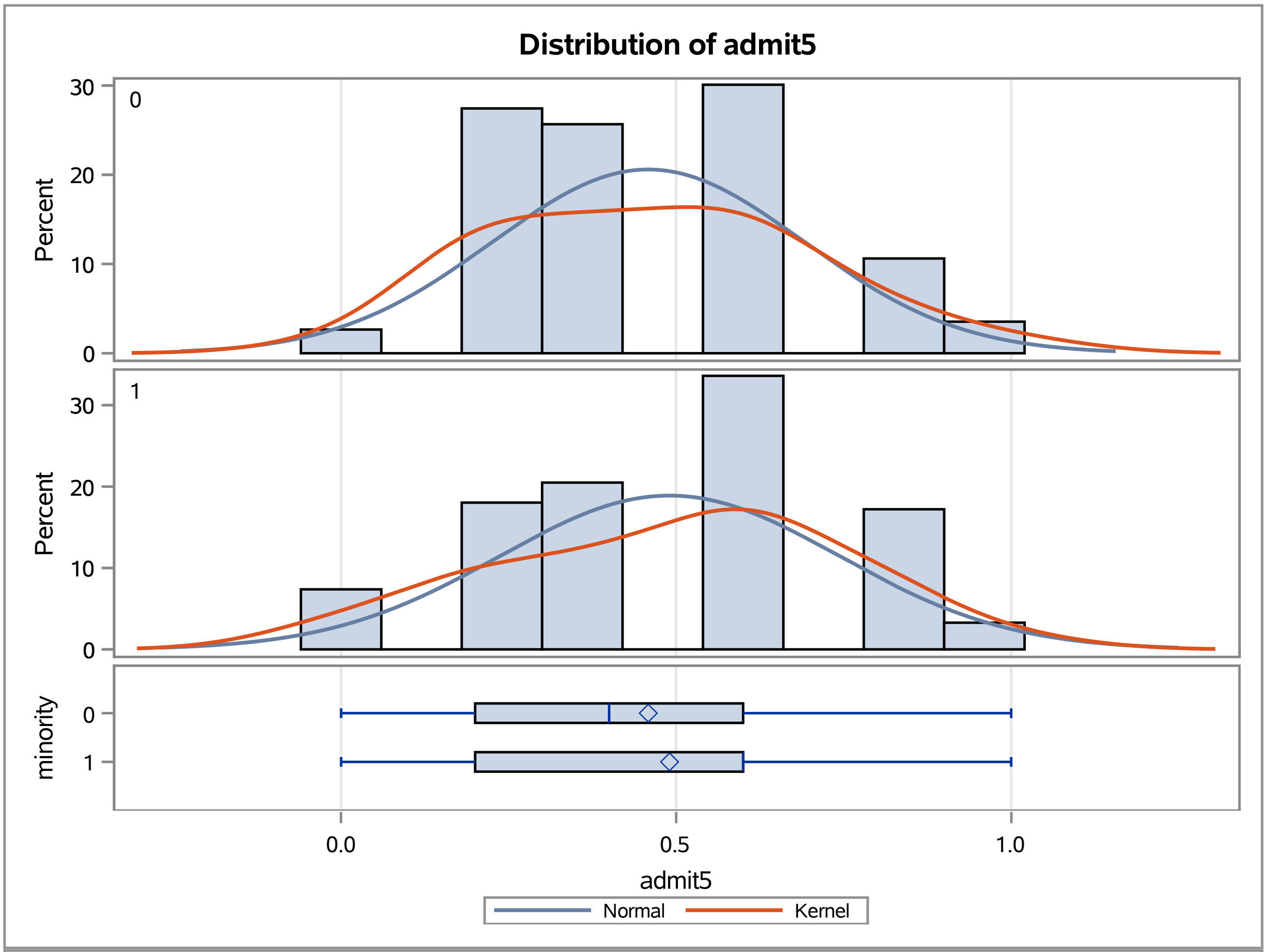

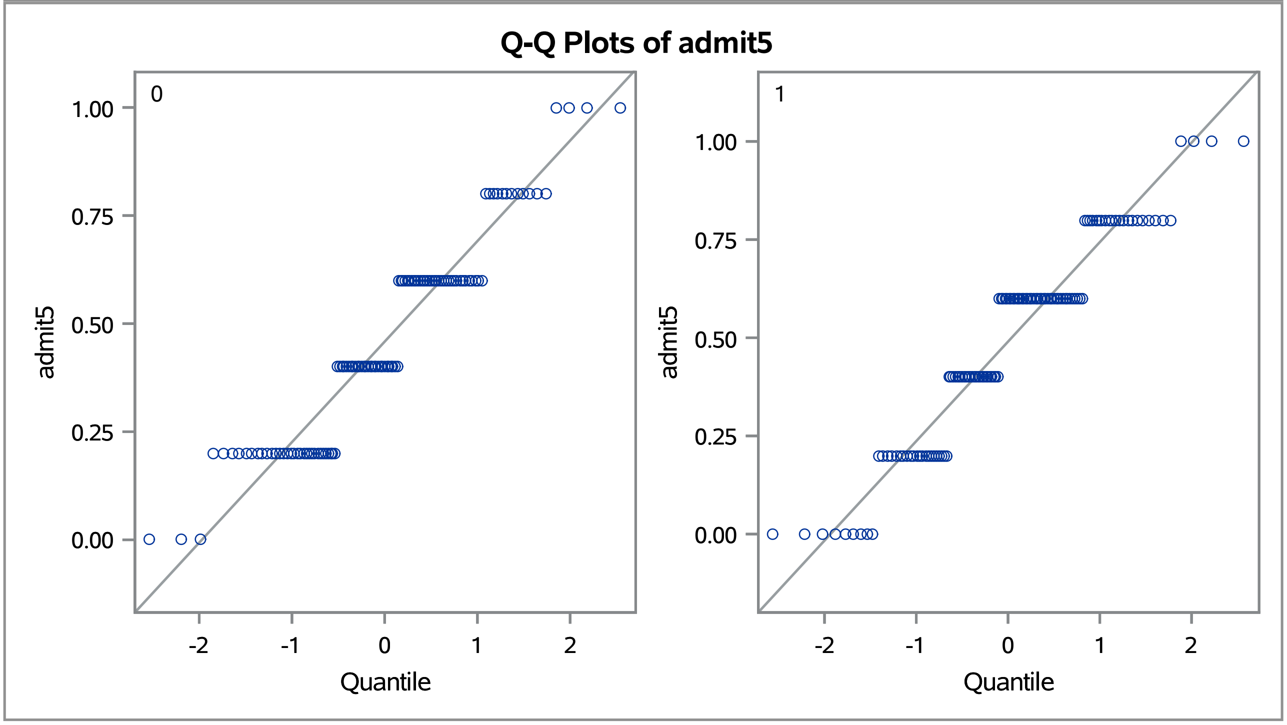

Lavie National survey
Significance testing for admit likelihood

The Mixed Procedure

Model Information	
**Data Set**	WORK.NAT4B
**Dependent Variable**	admit5
**Covariance Structure**	Diagonal
**Estimation Method**	REML
**Residual Variance Method**	Profile
**Fixed Effects SE Method**	Model-Based
**Degrees of Freedom Method**	Residual

Class Level Information		
**Class**	**Levels**	**Values**
**agecat3**	3	1 2 3

Dimensions	
**Covariance Parameters**	1
**Columns in X**	4
**Columns in Z**	0
**Subjects**	1
**Max Obs per Subject**	245

Number of Observations	
**Number of Observations Read**	246
**Number of Observations Used**	245
**Number of Observations Not Used**	1

Covariance Parameter Estimates	
**Cov Parm**	**Estimate**
**Residual**	0.05962

Fit Statistics	
**-2 Res Log Likelihood**	17.5
**AIC (Smaller is Better)**	19.5
**AICC (Smaller is Better)**	19.5
**BIC (Smaller is Better)**	23.0

Lavie National survey
Significance testing for admit likelihood

The Mixed Procedure

Type 3 Tests of Fixed Effects				
Effect	Num DF	Den DF	F Value	Pr > F
**agecat3**	2	242	1.15	0.3176

Least Squares Means						
Effect	agecat3	Estimate	Standard Error	DF	t Value	Pr > |t|
**agecat3**	1	0.4447	0.02801	242	15.88	<.0001
**agecat3**	2	0.4847	0.02318	242	20.91	<.0001
**agecat3**	3	0.5069	0.03206	242	15.81	<.0001

Lavie National survey
Significance testing for admit likelihood

The TTEST Procedure

Variable: admit5

status2	Method	N	Mean	Std Dev	Std Err	Minimum	Maximum
0		96	0.4729	0.2614	0.0267	0	1.0000
1		147	0.4776	0.2334	0.0193	0	1.0000
Diff (1-2)	Pooled		-0.00463	0.2448	0.0321		
Diff (1-2)	Satterthwaite		-0.00463		0.0329		

status2	Method	Mean	95% CL Mean		Std Dev	95% CL Std Dev	
0		0.4729	0.4200	0.5259	0.2614	0.2289	0.3046
1		0.4776	0.4395	0.5156	0.2334	0.2095	0.2637
Diff (1-2)	Pooled	-0.00463	-0.0679	0.0587	0.2448	0.2248	0.2688
Diff (1-2)	Satterthwaite	-0.00463	-0.0695	0.0603			

Method	Variances	DF	t Value	Pr > |t|
Pooled	Equal	241	-0.14	0.8854
Satterthwaite	Unequal	186.78	-0.14	0.8881

Equality of Variances				
Method	Num DF	Den DF	F Value	Pr > F
Folded F	95	146	1.25	0.2182

Lavie National survey
Significance testing for admit likelihood

The TTEST Procedure

Variable: admit5

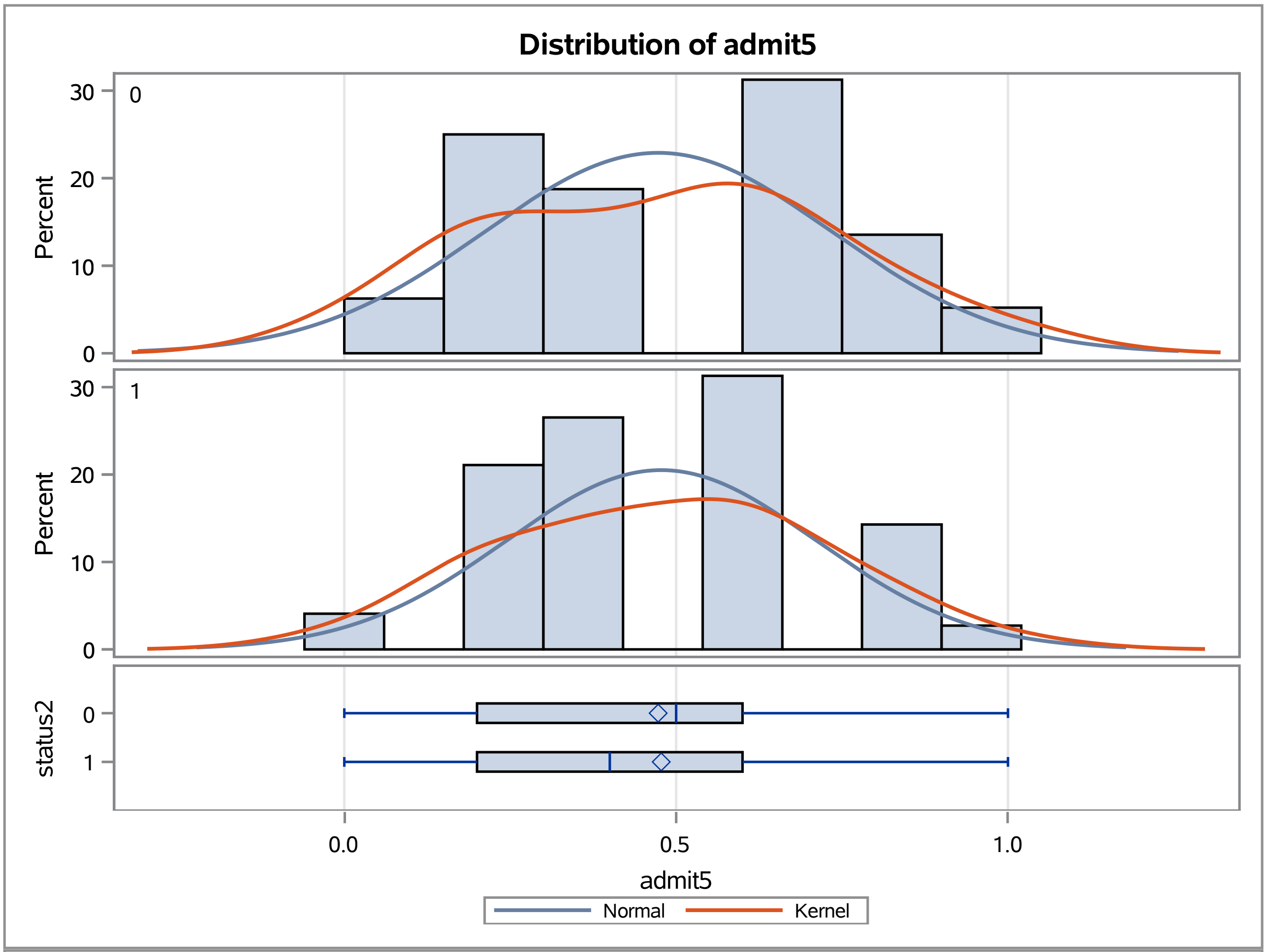

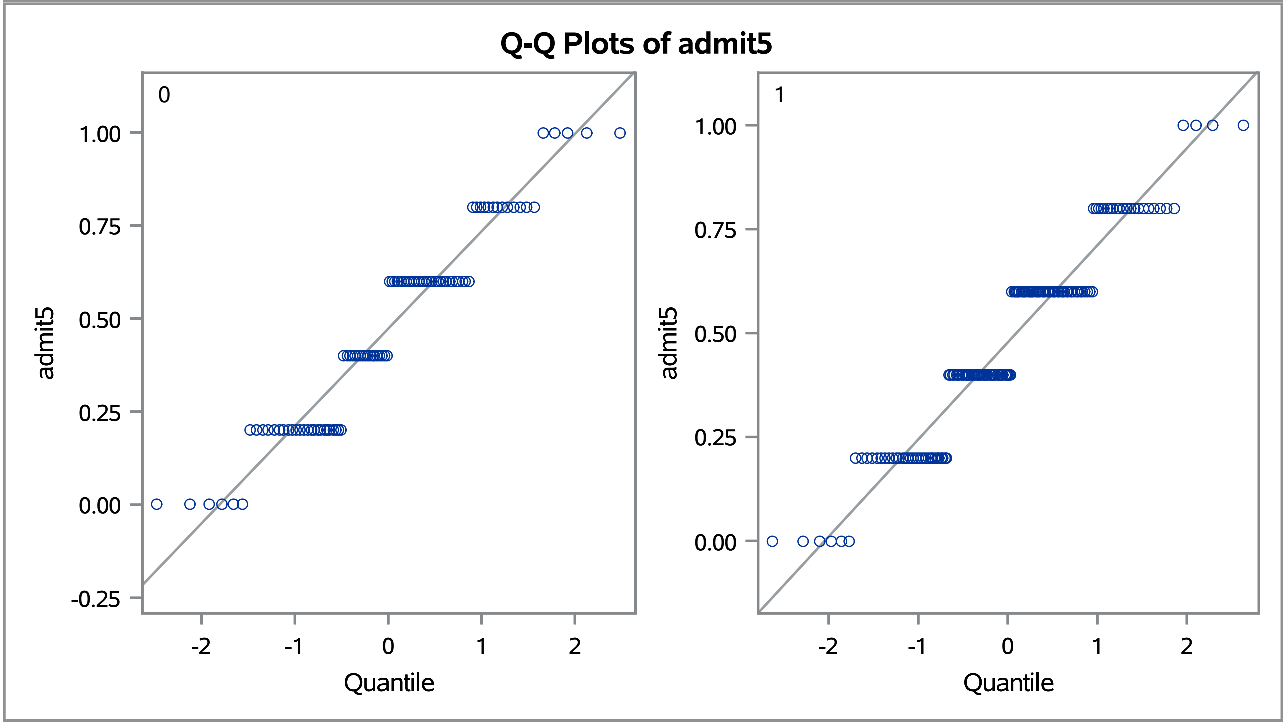

Lavie National survey
Significance testing for admit likelihood

The Mixed Procedure

Model Information	
**Data Set**	WORK.NAT4B
**Dependent Variable**	admit5
**Covariance Structure**	Diagonal
**Estimation Method**	REML
**Residual Variance Method**	Profile
**Fixed Effects SE Method**	Model-Based
**Degrees of Freedom Method**	Residual

Class Level Information		
**Class**	**Levels**	**Values**
**Site_region**	5	1 2 3 4 5

Dimensions	
**Covariance Parameters**	1
**Columns in X**	6
**Columns in Z**	0
**Subjects**	1
**Max Obs per Subject**	246

Number of Observations	
**Number of Observations Read**	246
**Number of Observations Used**	246
**Number of Observations Not Used**	0

Covariance Parameter Estimates	
**Cov Parm**	**Estimate**
**Residual**	0.05580

Fit Statistics	
**-2 Res Log Likelihood**	7.1
**AIC (Smaller is Better)**	9.1
**AICC (Smaller is Better)**	9.2
**BIC (Smaller is Better)**	12.6

Lavie National survey
Significance testing for admit likelihood

The Mixed Procedure

Type 3 Tests of Fixed Effects				
Effect	Num DF	Den DF	F Value	Pr > F
**Site_region**	4	241	6.03	0.0001

Least Squares Means						
Effect	Site_region	Estimate	Standard Error	DF	t Value	Pr > |t|
**Site_region**	1	0.6050	0.03735	241	16.20	<.0001
**Site_region**	2	0.6111	0.05568	241	10.98	<.0001
**Site_region**	3	0.4647	0.04051	241	11.47	<.0001
**Site_region**	4	0.4286	0.02386	241	17.96	<.0001
**Site_region**	5	0.4286	0.03157	241	13.58	<.0001

Differences of Least Squares Means							
Effect	Site_region	Site_region	Estimate	Standard Error	DF	t Value	Pr > |t|
**Site_region**	1	2	-0.00611	0.06705	241	-0.09	0.9275
**Site_region**	1	3	0.1403	0.05510	241	2.55	0.0115
**Site_region**	1	4	0.1764	0.04432	241	3.98	<.0001
**Site_region**	1	5	0.1764	0.04890	241	3.61	0.0004
**Site_region**	2	3	0.1464	0.06886	241	2.13	0.0345
**Site_region**	2	4	0.1825	0.06058	241	3.01	0.0029
**Site_region**	2	5	0.1825	0.06401	241	2.85	0.0047
**Site_region**	3	4	0.03613	0.04702	241	0.77	0.4429
**Site_region**	3	5	0.03613	0.05136	241	0.70	0.4824
**Site_region**	4	5	-336E-18	0.03957	241	-0.00	1.0000

Lavie National survey
Significance testing for admit likelihood predictors for attendings only

The TTEST Procedure

Variable: admit5

er_exp	Method	N	Mean	Std Dev	Std Err	Minimum	Maximum
0		50	0.4720	0.2673	0.0378	0	1.0000
1		46	0.4739	0.2577	0.0380	0	1.0000
Diff (1-2)	Pooled		-0.00191	0.2627	0.0537		
Diff (1-2)	Satterthwaite		-0.00191		0.0536		

er_exp	Method	Mean	95% CL Mean		Std Dev	95% CL Std Dev	
0		0.4720	0.3960	0.5480	0.2673	0.2233	0.3331
1		0.4739	0.3974	0.5504	0.2577	0.2138	0.3246
Diff (1-2)	Pooled	-0.00191	-0.1085	0.1047	0.2627	0.2300	0.3065
Diff (1-2)	Satterthwaite	-0.00191	-0.1083	0.1045			

Method	Variances	DF	t Value	Pr > |t|
Pooled	Equal	94	-0.04	0.9716
Satterthwaite	Unequal	93.786	-0.04	0.9716

Equality of Variances				
Method	Num DF	Den DF	F Value	Pr > F
Folded F	49	45	1.08	0.8070

Lavie National survey
Significance testing for admit likelihood predictors for attendings only

The TTEST Procedure

Variable: admit5

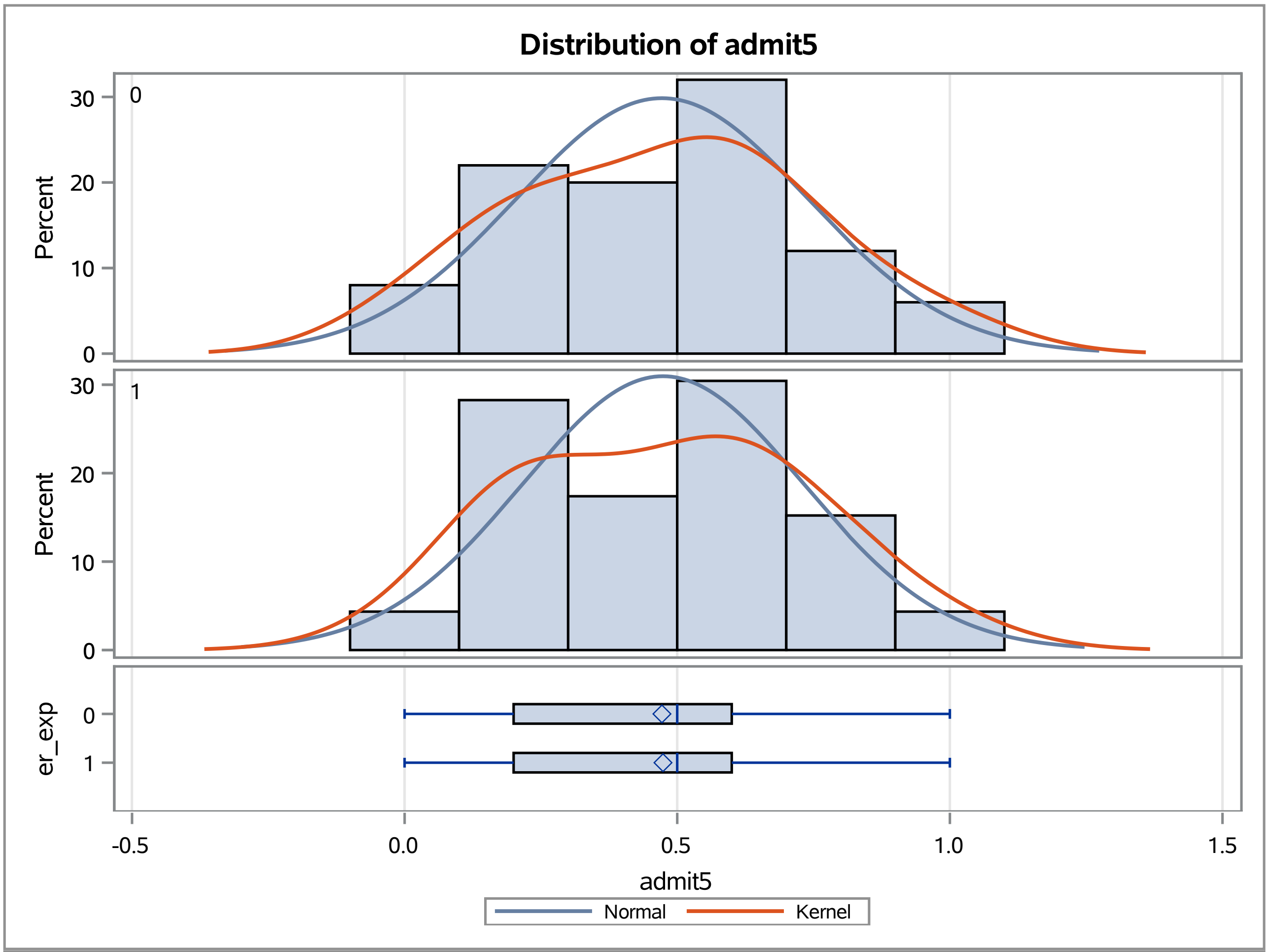

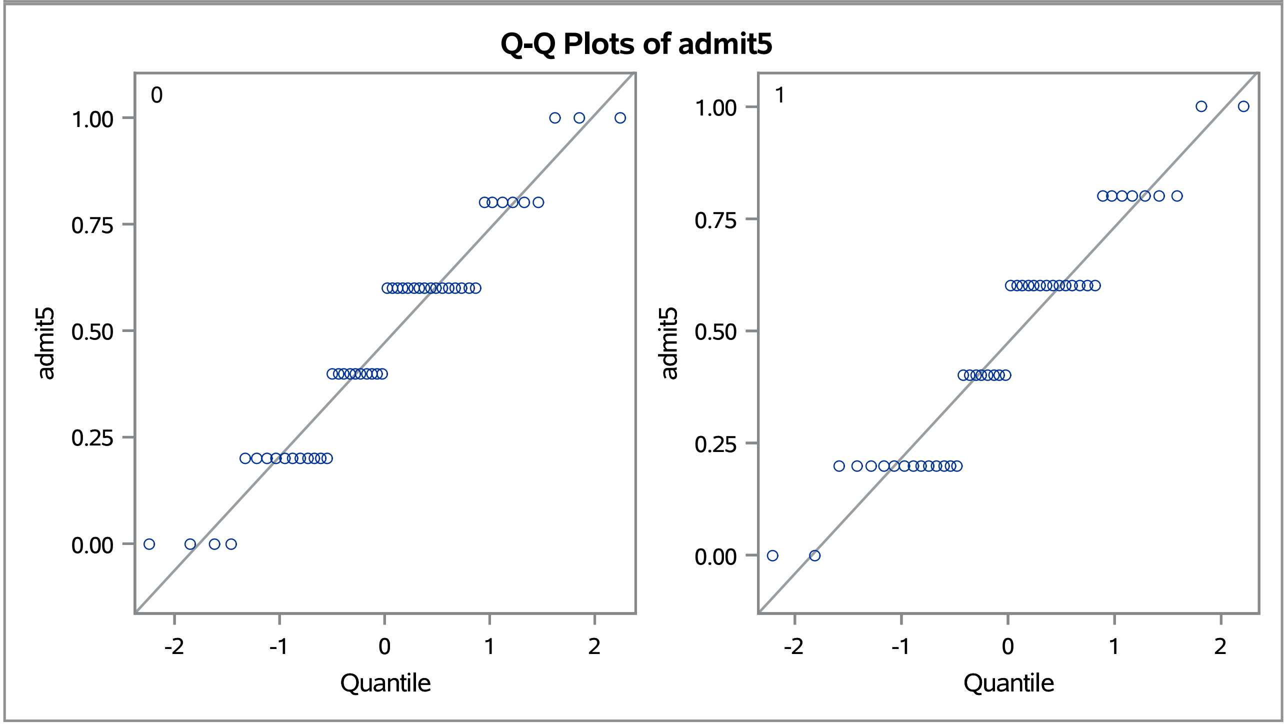

Lavie National survey
Significance testing for admit likelihood predictors for attendings only

The TTEST Procedure

Variable: admit5

EPS	Method	N	Mean	Std Dev	Std Err	Minimum	Maximum
**0**		65	0.4585	0.2709	0.0336	0	1.0000
**1**		31	0.5032	0.2415	0.0434	0.2000	1.0000
**Diff (1-2)**	**Pooled**		-0.0448	0.2619	0.0572		
**Diff (1-2)**	**Satterthwaite**		-0.0448		0.0549		

EPS	Method	Mean	95% CL Mean		Std Dev	95% CL Std Dev	
**0**		0.4585	0.3913	0.5256	0.2709	0.2310	0.3276
**1**		0.5032	0.4146	0.5918	0.2415	0.1930	0.3228
**Diff (1-2)**	**Pooled**	-0.0448	-0.1583	0.0687	0.2619	0.2292	0.3055
**Diff (1-2)**	**Satterthwaite**	-0.0448	-0.1543	0.0648			

Method	Variances	DF	t Value	Pr > |t|
**Pooled**	Equal	94	-0.78	0.4356
**Satterthwaite**	Unequal	65.724	-0.82	0.4175

Equality of Variances				
**Method**	**Num DF**	**Den DF**	**F Value**	**Pr > F**
**Folded F**	64	30	1.26	0.4950

Lavie National survey
Significance testing for admit likelihood predictors for attendings only

The TTEST Procedure

Variable: admit5

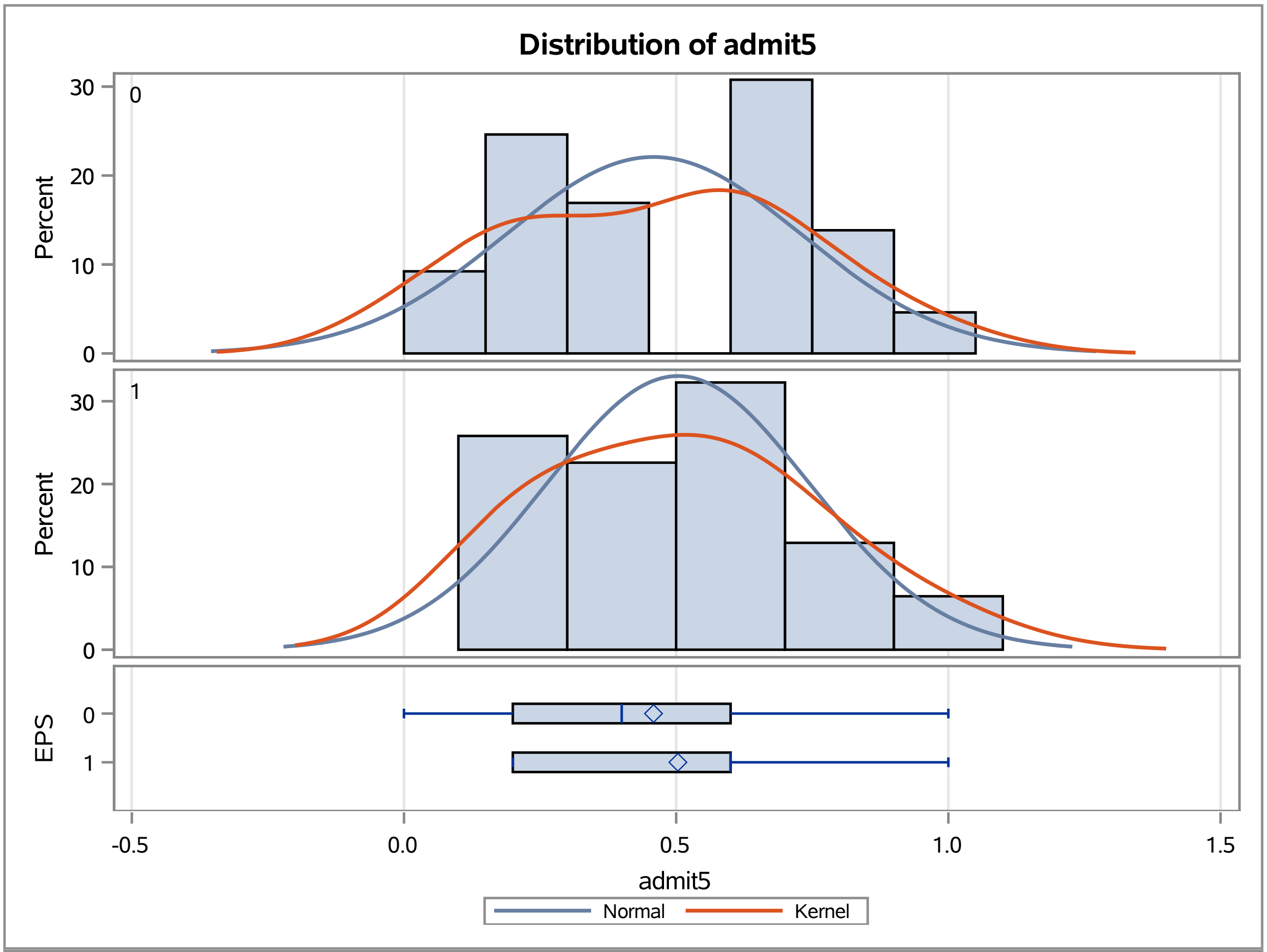

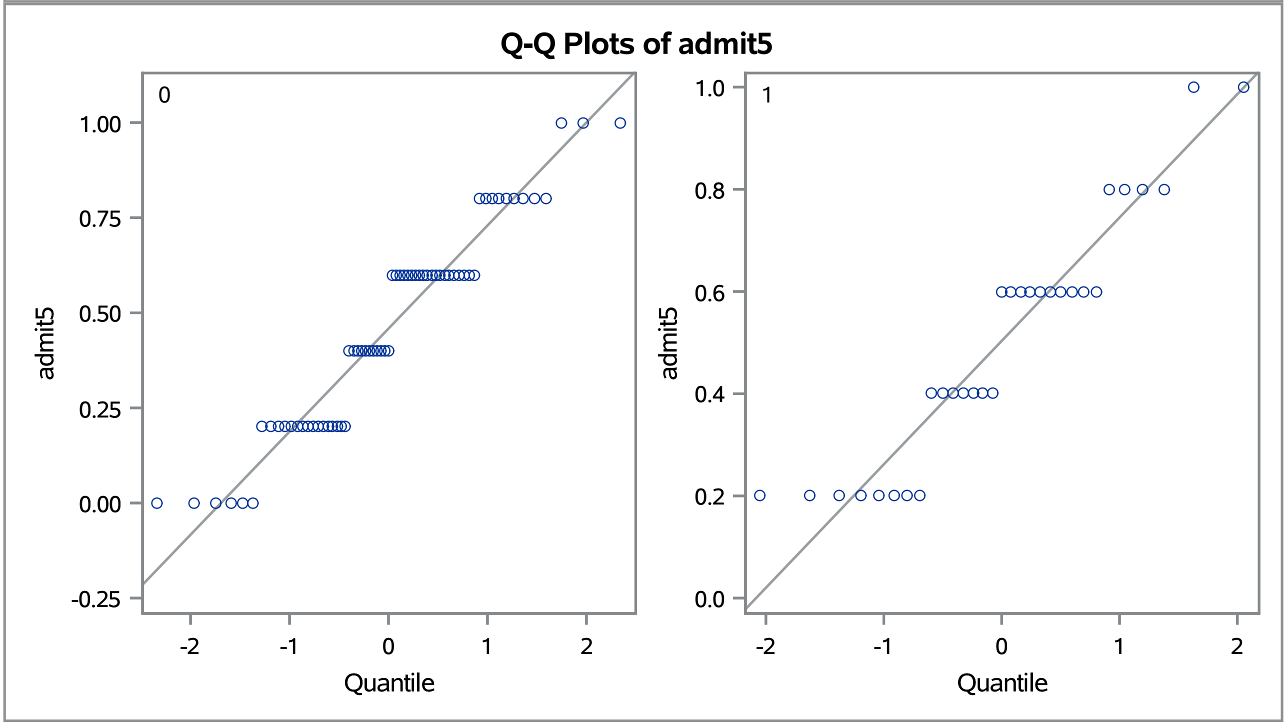

Lavie National survey
Significance testing for admit likelihood predictors for attendings only

The TTEST Procedure

Variable: admit5

inpx2	Method	N	Mean	Std Dev	Std Err	Minimum	Maximum
0		51	0.3686	0.2412	0.0338	0	0.8000
1		45	0.5911	0.2334	0.0348	0	1.0000
Diff (1-2)	Pooled		-0.2225	0.2376	0.0486		
Diff (1-2)	Satterthwaite		-0.2225		0.0485		

inpx2	Method	Mean	95% CL Mean		Std Dev	95% CL Std Dev	
0		0.3686	0.3008	0.4365	0.2412	0.2018	0.2999
1		0.5911	0.5210	0.6612	0.2334	0.1932	0.2948
Diff (1-2)	Pooled	-0.2225	-0.3190	-0.1260	0.2376	0.2079	0.2772
Diff (1-2)	Satterthwaite	-0.2225	-0.3188	-0.1262			

Method	Variances	DF	t Value	Pr > |t|
Pooled	Equal	94	-4.58	<.0001
Satterthwaite	Unequal	93.186	-4.59	<.0001

Equality of Variances				
Method	Num DF	Den DF	F Value	Pr > F
Folded F	50	44	1.07	0.8266

Lavie National survey
Significance testing for admit likelihood predictors for attendings only

The TTEST Procedure

Variable: admit5

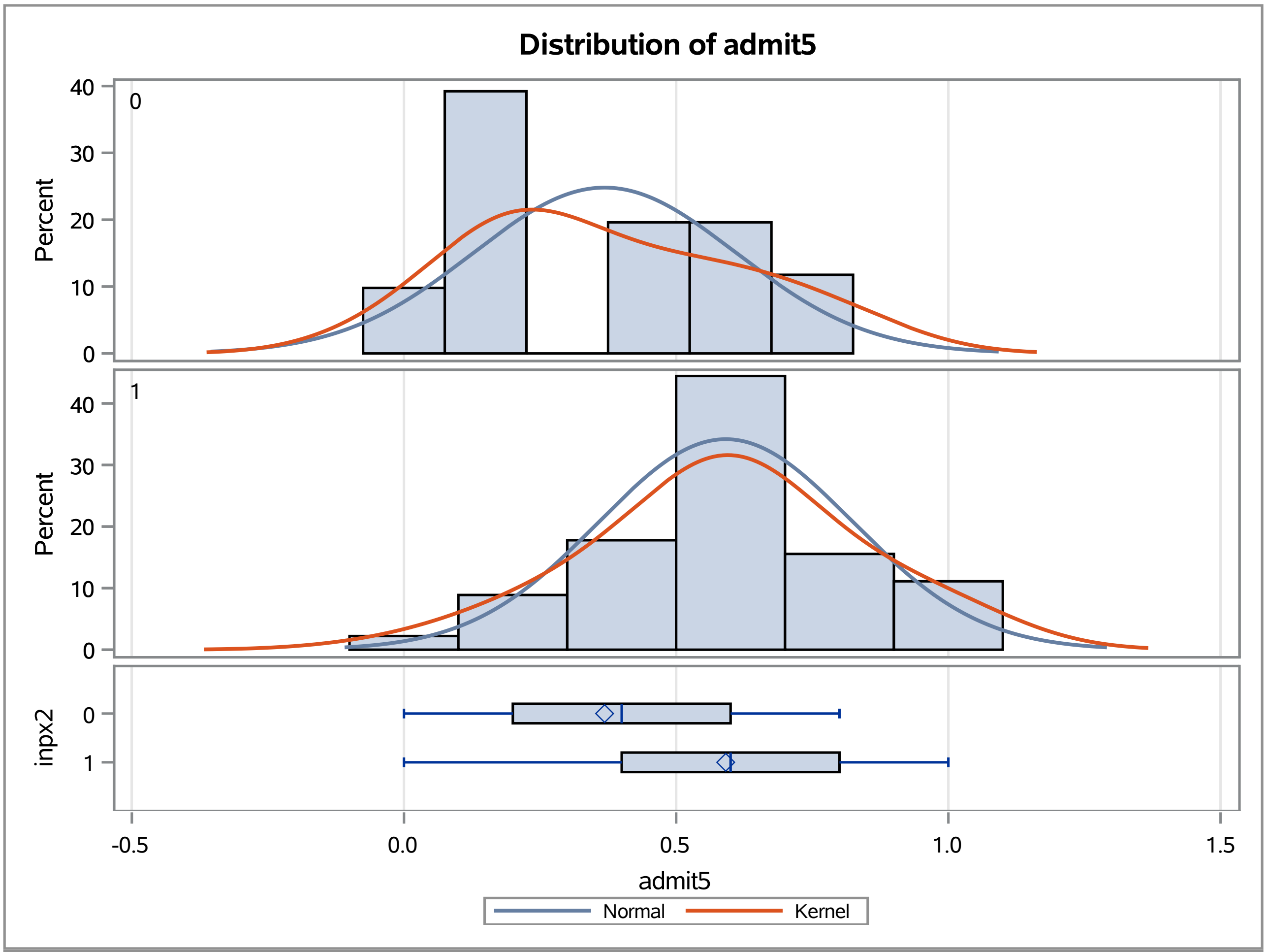

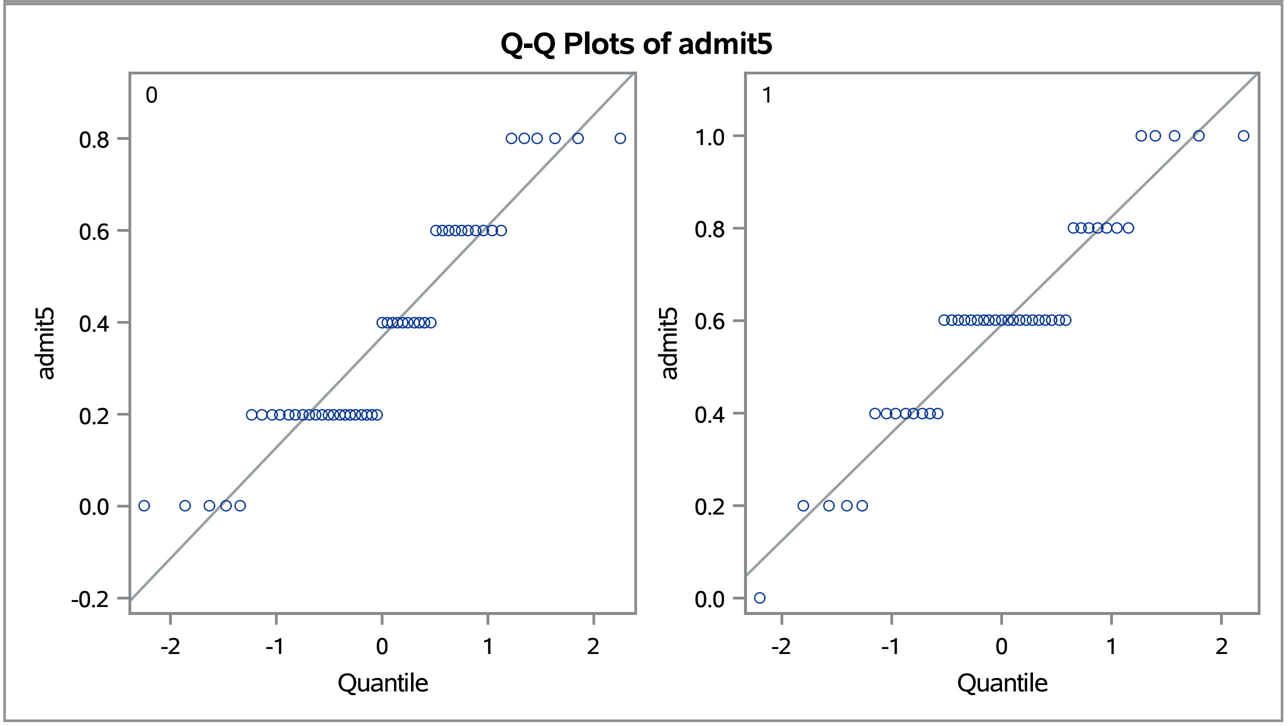

Lavie National survey
Significance testing for admit likelihood predictors for attendings only

The TTEST Procedure

Variable: admit5

outpx	Method	N	Mean	Std Dev	Std Err	Minimum	Maximum
**0**		47	0.5064	0.2665	0.0389	0	1.0000
**1**		49	0.4408	0.2549	0.0364	0	1.0000
**Diff (1-2)**	**Pooled**		0.0656	0.2607	0.0532		
**Diff (1-2)**	**Satterthwaite**		0.0656		0.0533		

outpx	Method	Mean	95% CL Mean		Std Dev	95% CL Std Dev	
**0**		0.5064	0.4281	0.5846	0.2665	0.2215	0.3348
**1**		0.4408	0.3676	0.5140	0.2549	0.2126	0.3184
**Diff (1-2)**	**Pooled**	0.0656	-0.0401	0.1712	0.2607	0.2281	0.3041
**Diff (1-2)**	**Satterthwaite**	0.0656	-0.0402	0.1713			

Method	Variances	DF	t Value	Pr > |t|
**Pooled**	Equal	94	1.23	0.2210
**Satterthwaite**	Unequal	93.298	1.23	0.2215

Equality of Variances				
**Method**	**Num DF**	**Den DF**	**F Value**	**Pr > F**
**Folded F**	46	48	1.09	0.7588

Lavie National survey
Significance testing for admit likelihood predictors for attendings only

The TTEST Procedure

Variable: admit5

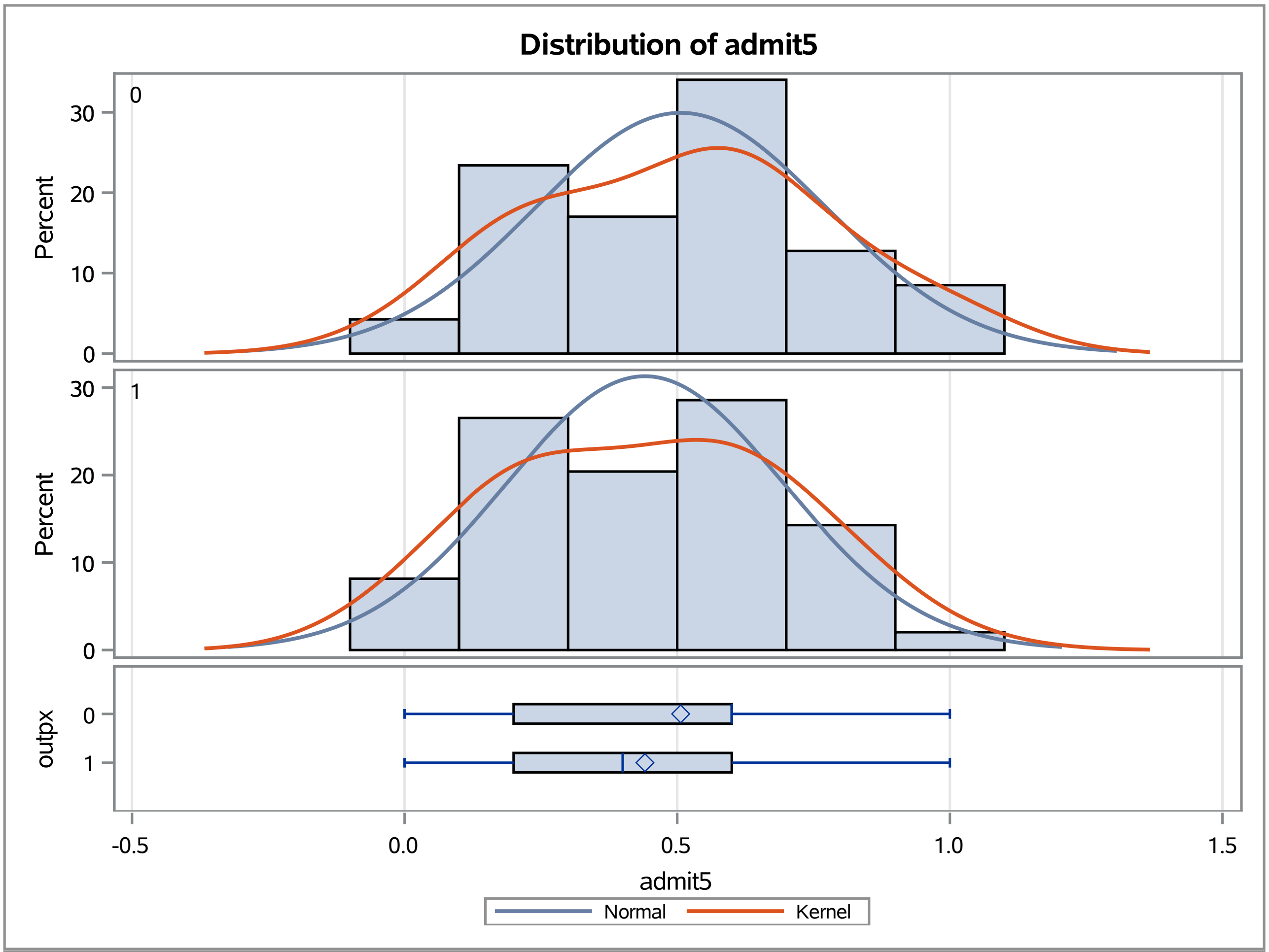

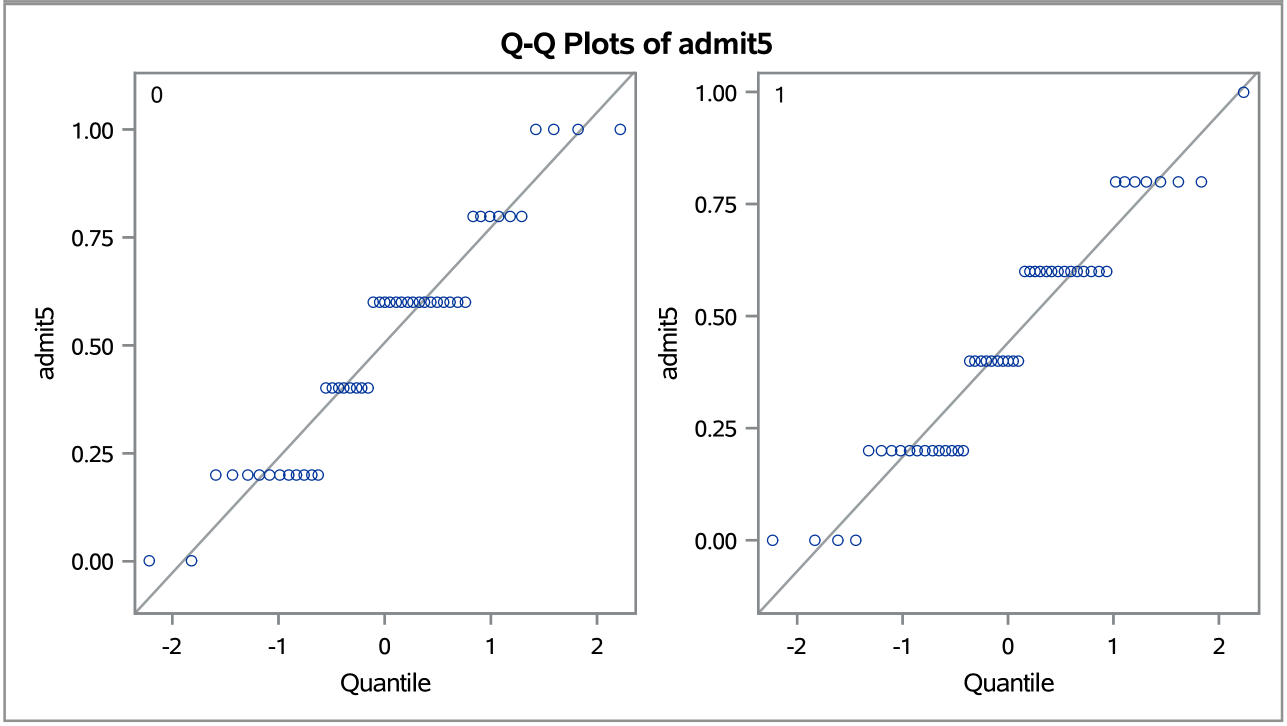

Lavie National survey
Significance testing for admit likelihood predictors for attendings only

The TTEST Procedure

Variable: admit5

pes	Method	N	Mean	Std Dev	Std Err	Minimum	Maximum
**0**		21	0.5810	0.2272	0.0496	0.2000	1.0000
**1**		23	0.4000	0.2558	0.0533	0	1.0000
**Diff (1-2)**	**Pooled**		0.1810	0.2426	0.0732		
**Diff (1-2)**	**Satterthwaite**		0.1810		0.0728		

pes	Method	Mean	95% CL Mean		Std Dev	95% CL Std Dev	
**0**		0.5810	0.4775	0.6844	0.2272	0.1738	0.3281
**1**		0.4000	0.2894	0.5106	0.2558	0.1979	0.3621
**Diff (1-2)**	**Pooled**	0.1810	0.0332	0.3287	0.2426	0.2001	0.3084
**Diff (1-2)**	**Satterthwaite**	0.1810	0.0340	0.3279			

Method	Variances	DF	t Value	Pr > |t|
**Pooled**	Equal	42	2.47	0.0176
**Satterthwaite**	Unequal	41.973	2.48	0.0170

Equality of Variances				
**Method**	**Num DF**	**Den DF**	**F Value**	**Pr > F**
**Folded F**	22	20	1.27	0.5972

Lavie National survey
Significance testing for admit likelihood predictors for attendings only

The TTEST Procedure

Variable: admit5

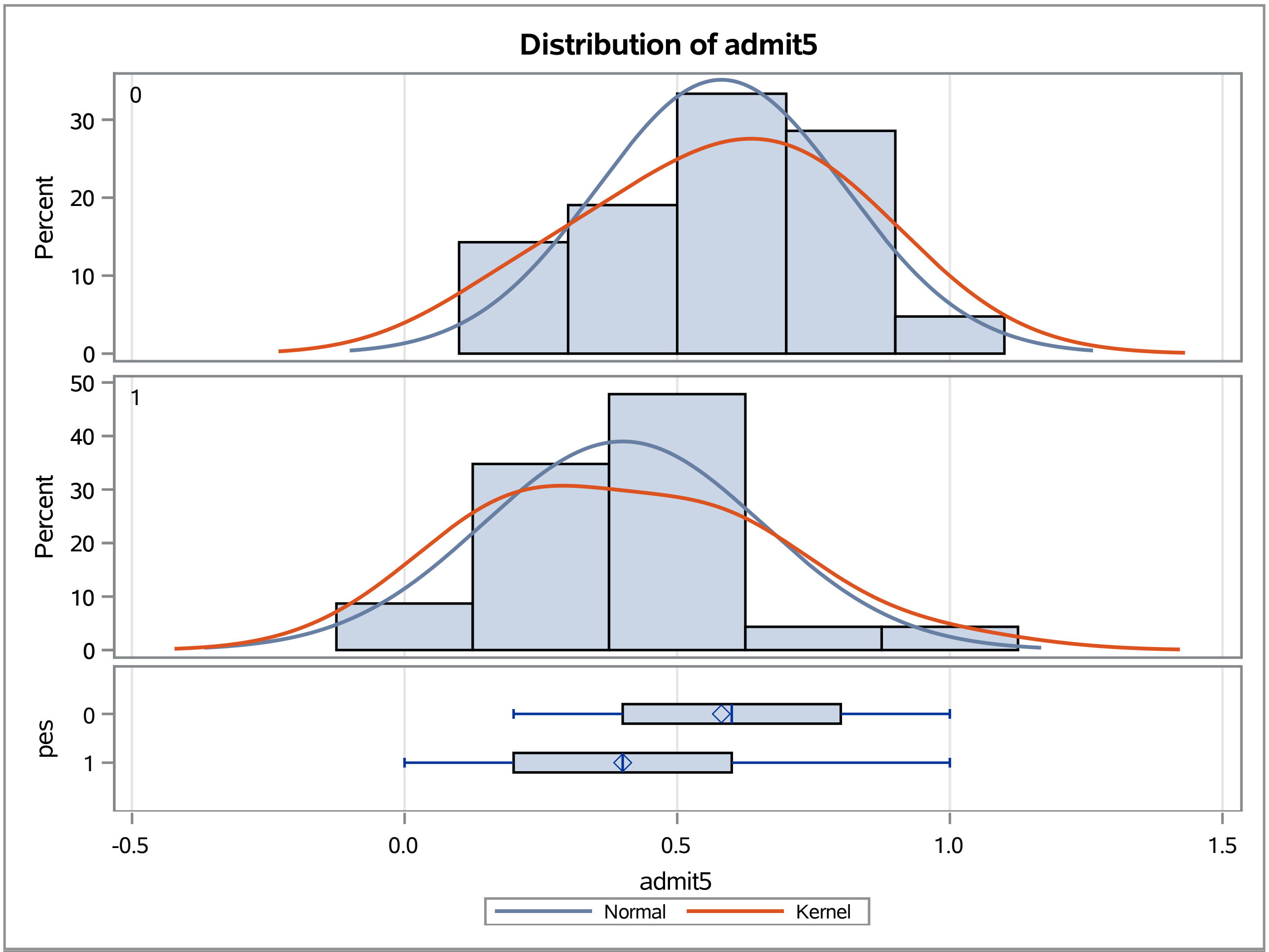

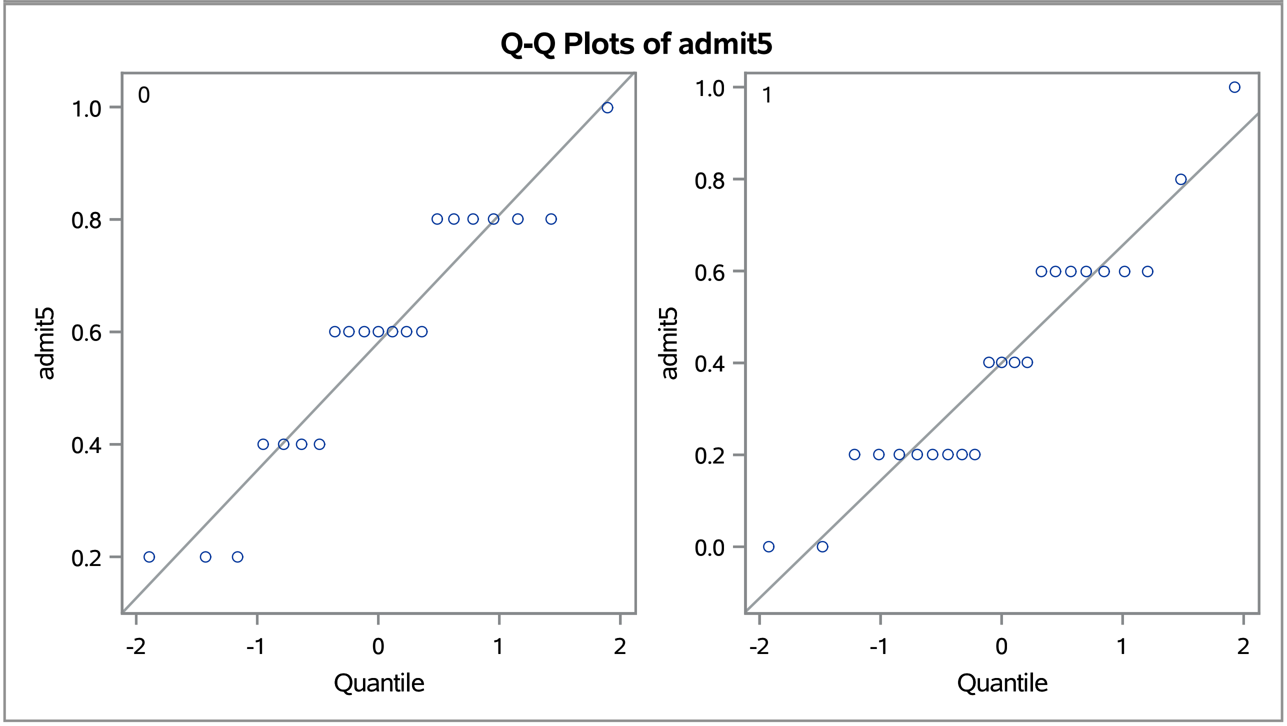

Lavie National survey
Significance testing for admit likelihood predictors for attendings only

The TTEST Procedure

Variable: admit5

training_pes	Method	N	Mean	Std Dev	Std Err	Minimum	Maximum
0		42	0.5333	0.2486	0.0384	0	1.0000
1		54	0.4259	0.2636	0.0359	0	1.0000
Diff (1-2)	Pooled		0.1074	0.2572	0.0529		
Diff (1-2)	Satterthwaite		0.1074		0.0525		

training_pes	Method	Mean	95% CL Mean		Std Dev	95% CL Std Dev	
0		0.5333	0.4559	0.6108	0.2486	0.2045	0.3170
1		0.4259	0.3540	0.4979	0.2636	0.2216	0.3255
Diff (1-2)	Pooled	0.1074	0.00235	0.2125	0.2572	0.2251	0.3000
Diff (1-2)	Satterthwaite	0.1074	0.00308	0.2117			

Method	Variances	DF	t Value	Pr > |t|
Pooled	Equal	94	2.03	0.0452
Satterthwaite	Unequal	90.523	2.05	0.0437

Equality of Variances				
Method	Num DF	Den DF	F Value	Pr > F
Folded F	53	41	1.12	0.7008

Lavie National survey
Significance testing for admit likelihood predictors for attendings only

The TTEST Procedure

Variable: admit5

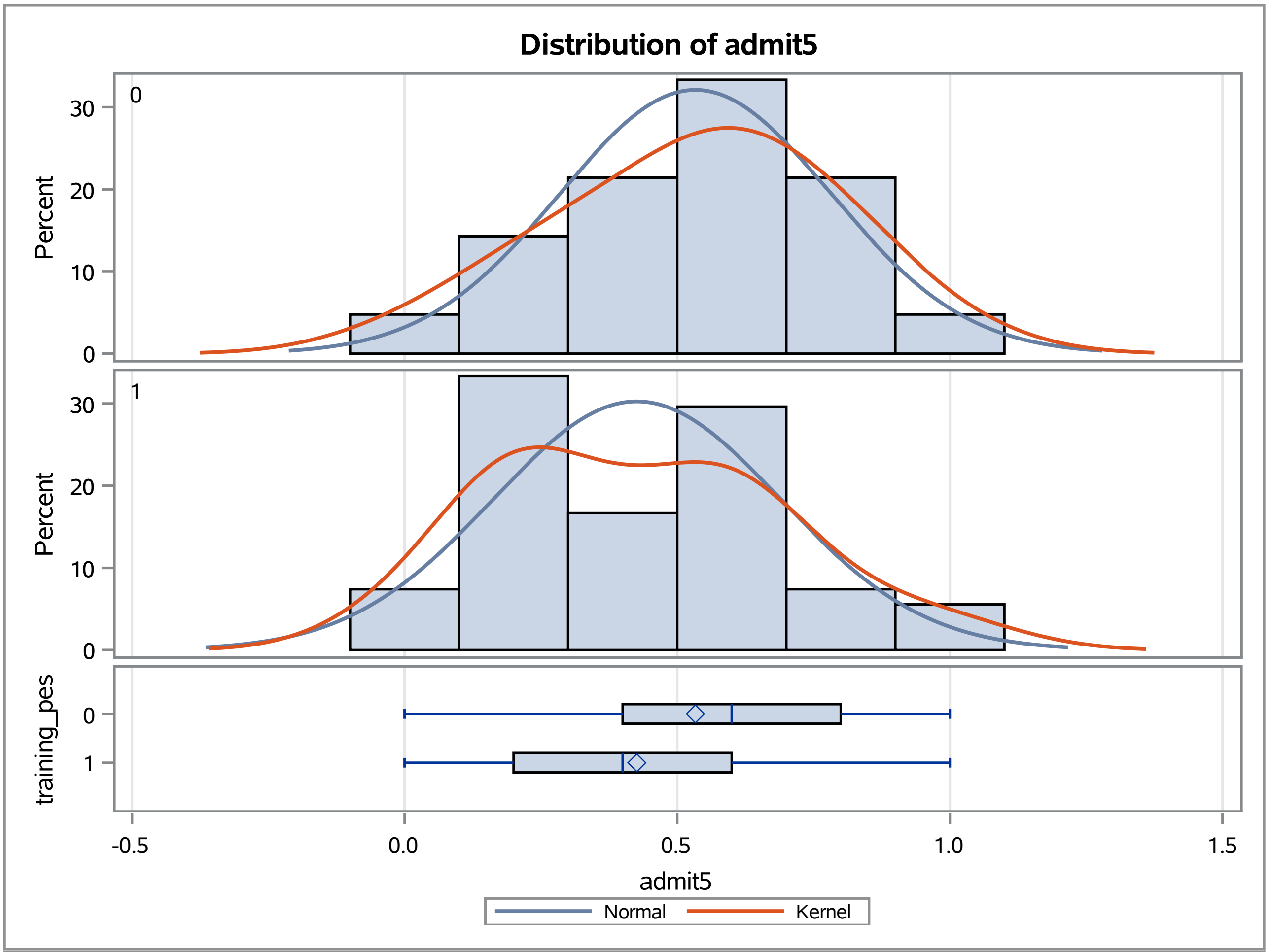

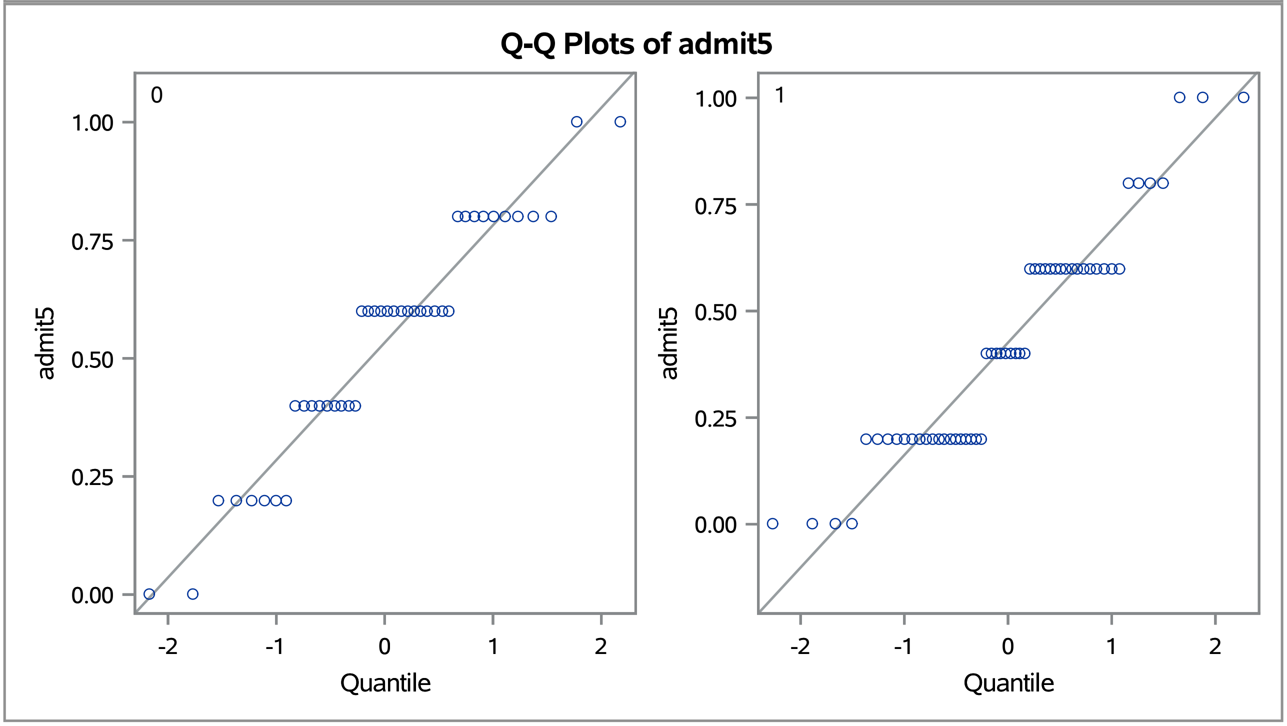

Lavie National survey
Significance testing for admit likelihood predictors for attendings only

The CORR Procedure

**2 With Variables:**	yearspracticing percent_er
**1 Variables:**	admit5

Simple Statistics							
**Variable**	**N**	**Mean**	**Std Dev**	**Sum**	**Minimum**	**Maximum**	**Label**
**yearspracticing**	96	2.58333	1.86754	248.00000	1.00000	9.00000	yearspracticing
**percent_er**	96	2.16667	1.63299	208.00000	1.00000	6.00000	percent_er
**admit5**	96	0.47292	0.26136	45.40000	0	1.00000	

Pearson Correlation Coefficients, N = 96<br>Prob > |r| under H0: Rho=0	
	**admit5**
**yearspracticing**<br>**yearspracticing**	0.14916<br>0.1469
**percent_er**<br>**percent_er**	0.03042<br>0.7686

Lavie National survey
Significance testing for admit likelihood predictors for trainees only

The CORR Procedure

**3 With Variables:**	PGY pgy2 time_er
**1 Variables:**	admit5

Simple Statistics							
**Variable**	**N**	**Mean**	**Std Dev**	**Sum**	**Minimum**	**Maximum**	**Label**
**PGY**	147	2.51701	1.20133	370.00000	1.00000	5.00000	PGY
**pgy2**	147	2.46259	1.10585	362.00000	1.00000	4.00000	
**time_er**	147	3.04082	0.78407	447.00000	1.00000	4.00000	time_er
**admit5**	147	0.47755	0.23344	70.20000	0	1.00000	

Pearson Correlation Coefficients, N = 147<br>Prob > |r| under H0: Rho=0	
	**admit5**
**PGY**<br>**PGY**	0.01725<br>0.8358
**pgy2**	-0.00726<br>0.9305
**time_er**<br>**time_er**	-0.06980<br>0.4008

Lavie National survey
Significance testing for admit likelihood
full sample (attendings + trainess) personality-related analyses

The CORR Procedure

**7 With Variables:**	paternalism worry_px use_laws risk_comfort ih_benefits perception_self_admit perception_inst_admit
**1 Variables:**	admit5

Simple Statistics							
**Variable**	**N**	**Mean**	**Std Dev**	**Sum**	**Minimum**	**Maximum**	**Label**
**paternalism**	240	2.69083	0.66893	645.80000	1.00000	4.40000	
**worry_px**	240	2.14167	0.50596	514.00000	1.00000	4.00000	worry_px
**use_laws**	241	3.08714	0.83459	744.00000	1.00000	4.00000	use_laws
**risk_comfort**	241	2.95436	0.62081	712.00000	1.00000	4.00000	risk_comfort
**ih_benefits**	241	2.56846	0.58138	619.00000	2.00000	4.00000	ih_benefits
**perception_self_admit**	241	2.02075	0.57335	487.00000	1.00000	3.00000	perception_self_admit
**perception_inst_admit**	240	1.84167	0.62709	442.00000	1.00000	3.00000	perception_inst_admit
**admit5**	246	0.47561	0.24573	117.00000	0	1.00000	

Pearson Correlation Coefficients<br>Prob > |r| under H0: Rho=0<br>Number of Observations	
	**admit5**
**paternalism**	0.14787<br>0.0219<br>240
**worry_px**<br>**worry_px**	0.08752<br>0.1766<br>240
**use_laws**<br>**use_laws**	-0.04451<br>0.4916<br>241
**risk_comfort**<br>**risk_comfort**	0.03917<br>0.5451<br>241
**ih_benefits**<br>**ih_benefits**	0.18867<br>0.0033<br>241
**perception_self_admit**<br>**perception_self_admit**	-0.21276<br>0.0009<br>241
**perception_inst_admit**<br>**perception_inst_admit**	-0.15081<br>0.0194<br>240

Lavie National survey
Significance testing for ~~admit likelihood~~

These next pages (1-25) are for
Confidence in admit/discharge decision
(avgabscon5)

The TTEST Procedure

Variable: avgabscon5

malefemale	Method	N	Mean	Std Dev	Std Err	Minimum	Maximum
**0**		117	66.4479	14.9451	1.3817	21.0000	100.0
**1**		100	69.9020	13.3880	1.3388	41.0000	100.0
**Diff (1-2)**	**Pooled**		-3.4541	14.2492	1.9406		
**Diff (1-2)**	**Satterthwaite**		-3.4541		1.9239		

malefemale	Method	Mean	95% CL Mean		Std Dev	95% CL Std Dev	
**0**		66.4479	63.7113	69.1844	14.9451	13.2445	17.1506
**1**		69.9020	67.2455	72.5585	13.3880	11.7547	15.5525
**Diff (1-2)**	**Pooled**	-3.4541	-7.2791	0.3708	14.2492	13.0203	15.7364
**Diff (1-2)**	**Satterthwaite**	-3.4541	-7.2463	0.3380			

Method	Variances	DF	t Value	Pr > |t|
**Pooled**	Equal	215	-1.78	0.0765
**Satterthwaite**	Unequal	214.51	-1.80	0.0740

Equality of Variances				
**Method**	**Num DF**	**Den DF**	**F Value**	**Pr > F**
**Folded F**	116	99	1.25	0.2606

Lavie National survey
Significance testing for admit likelihood

The TTEST Procedure

Variable: avgabscon5

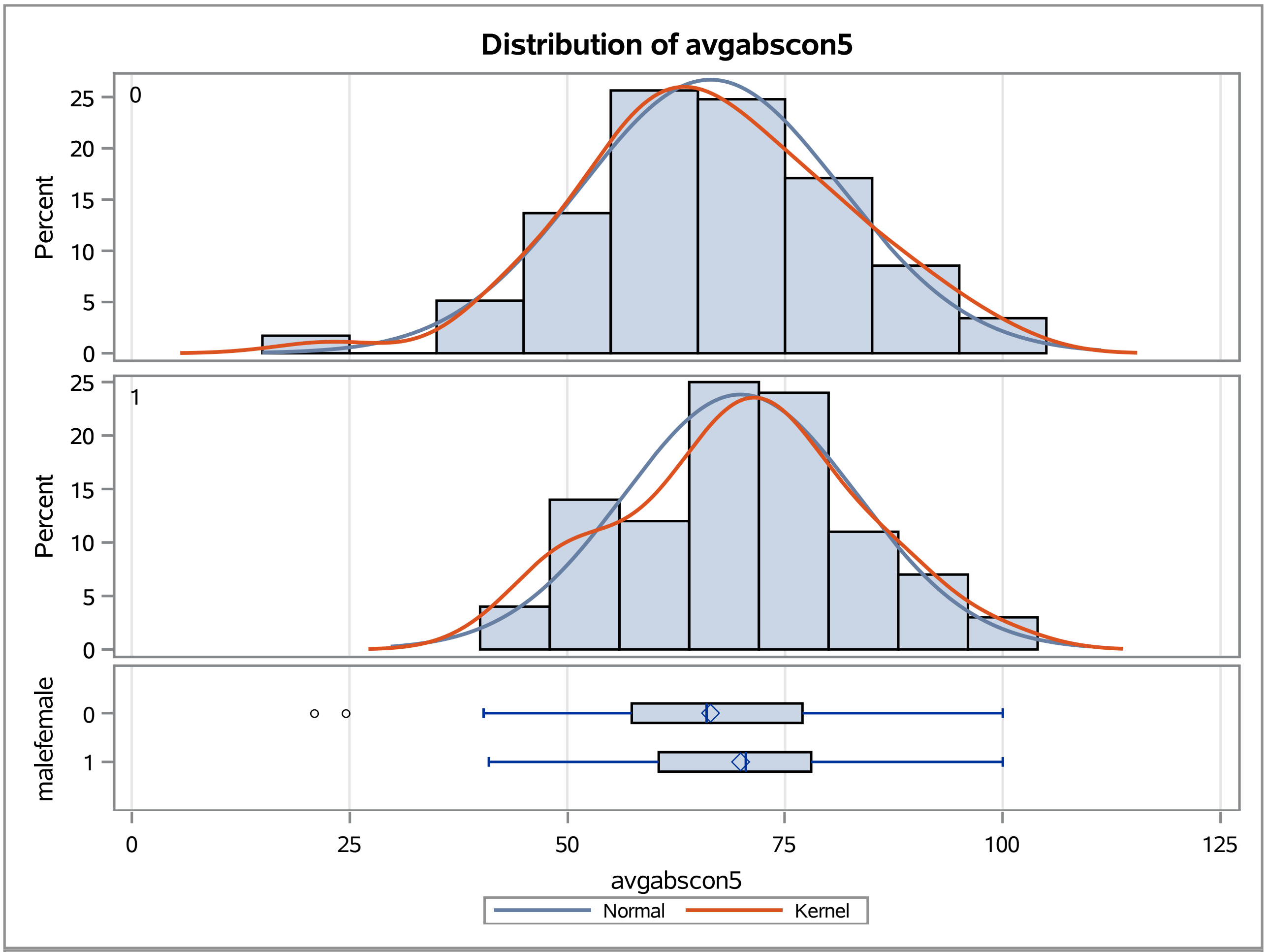

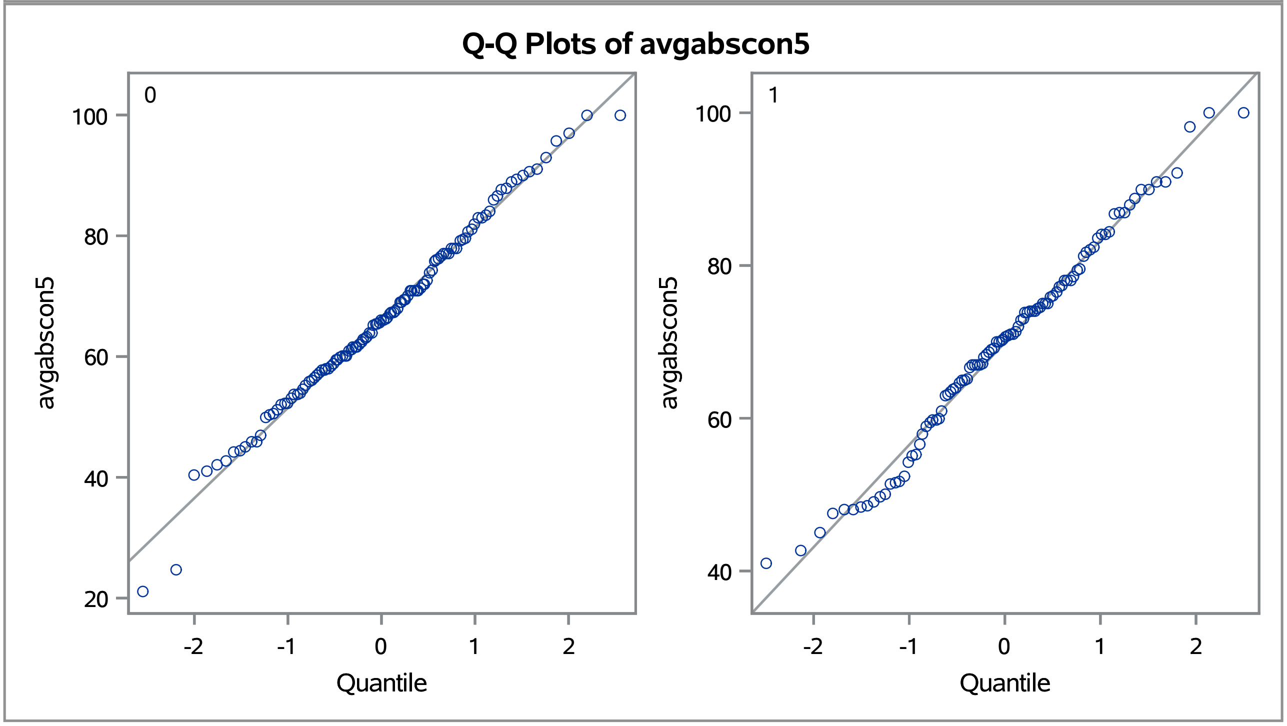

Lavie National survey
Significance testing for admit likelihood

The TTEST Procedure

Variable: avgabscon5

minority	Method	N	Mean	Std Dev	Std Err	Minimum	Maximum
**0**		105	66.8857	13.7199	1.3389	40.4000	100.0
**1**		108	69.6796	13.9969	1.3469	24.6000	100.0
**Diff (1-2)**	**Pooled**		-2.7939	13.8611	1.8997		
**Diff (1-2)**	**Satterthwaite**		-2.7939		1.8991		

minority	Method	Mean	95% CL Mean		Std Dev	95% CL Std Dev	
**0**		66.8857	64.2306	69.5409	13.7199	12.0819	15.8758
**1**		69.6796	67.0096	72.3496	13.9969	12.3466	16.1606
**Diff (1-2)**	**Pooled**	-2.7939	-6.5387	0.9509	13.8611	12.6553	15.3228
**Diff (1-2)**	**Satterthwaite**	-2.7939	-6.5376	0.9498			

Method	Variances	DF	t Value	Pr > |t|
**Pooled**	Equal	211	-1.47	0.1429
**Satterthwaite**	Unequal	210.99	-1.47	0.1427

Equality of Variances				
**Method**	**Num DF**	**Den DF**	**F Value**	**Pr > F**
**Folded F**	107	104	1.04	0.8385

Lavie National survey
Significance testing for admit likelihood

The TTEST Procedure

Variable: avgabscon5

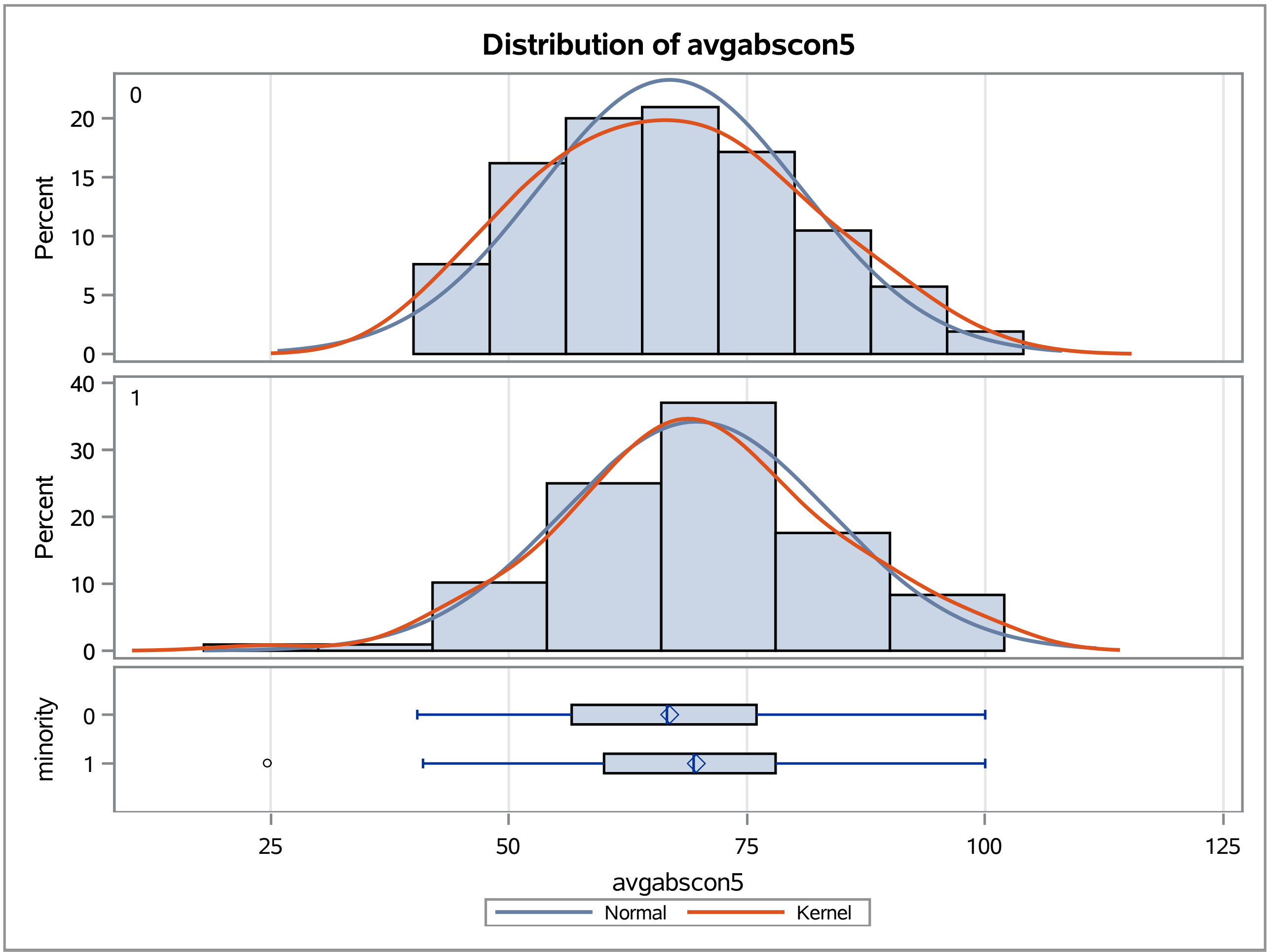

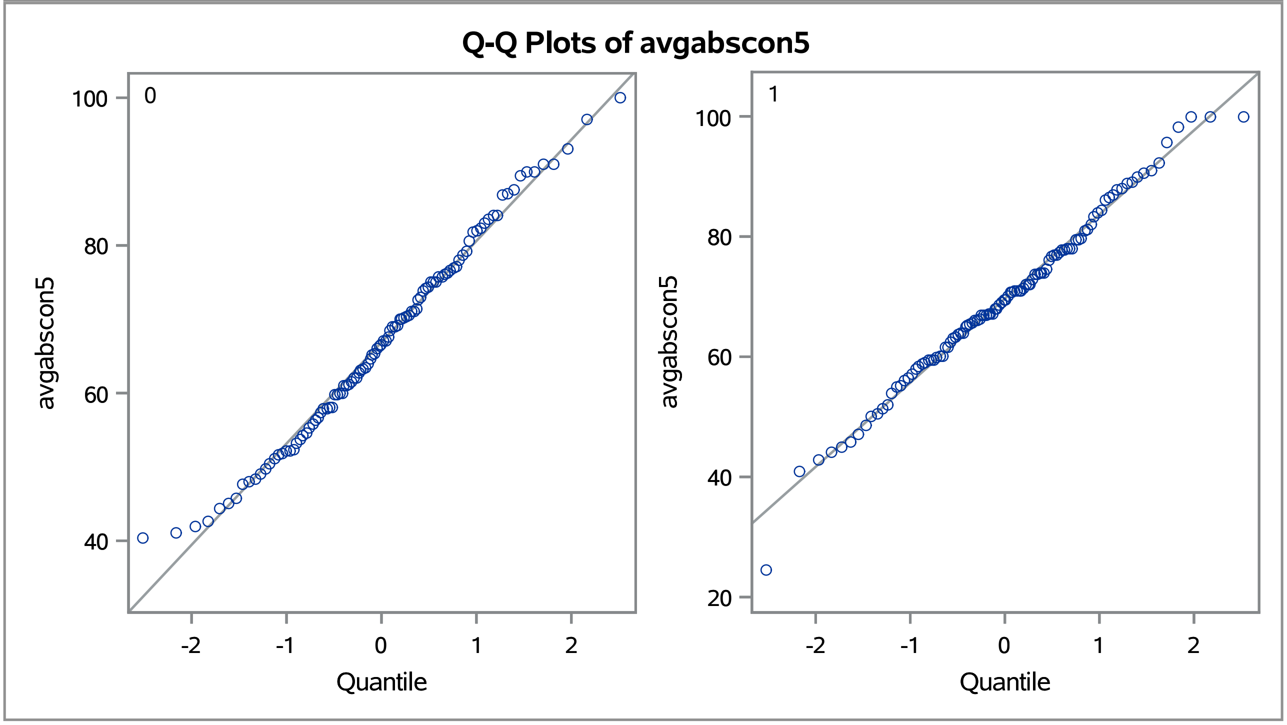

Lavie National survey
Significance testing for admit likelihood

The Mixed Procedure

Model Information	
**Data Set**	WORK.NAT4B
**Dependent Variable**	avgabscon5
**Covariance Structure**	Diagonal
**Estimation Method**	REML
**Residual Variance Method**	Profile
**Fixed Effects SE Method**	Model-Based
**Degrees of Freedom Method**	Residual

Class Level Information		
**Class**	**Levels**	**Values**
**agecat3**	3	1 2 3

Dimensions	
**Covariance Parameters**	1
**Columns in X**	4
**Columns in Z**	0
**Subjects**	1
**Max Obs per Subject**	222

Number of Observations	
**Number of Observations Read**	246
**Number of Observations Used**	222
**Number of Observations Not Used**	24

Covariance Parameter Estimates	
**Cov Parm**	**Estimate**
**Residual**	201.35

Fit Statistics	
**-2 Res Log Likelihood**	1796.1
**AIC (Smaller is Better)**	1798.1
**AICC (Smaller is Better)**	1798.1
**BIC (Smaller is Better)**	1801.5

Lavie National survey
Significance testing for admit likelihood

The Mixed Procedure

Type 3 Tests of Fixed Effects				
Effect	Num DF	Den DF	F Value	Pr > F
**agecat3**	2	219	1.27	0.2820

Least Squares Means						
Effect	agecat3	Estimate	Standard Error	DF	t Value	Pr > |t|
**agecat3**	1	67.8192	1.6608	219	40.84	<.0001
**agecat3**	2	66.6866	1.4407	219	46.29	<.0001
**agecat3**	3	70.5692	1.9678	219	35.86	<.0001

Lavie National survey
Significance testing for admit likelihood

The TTEST Procedure

Variable: avgabscon5

status2	Method	N	Mean	Std Dev	Std Err	Minimum	Maximum
0		85	68.8094	13.8698	1.5044	40.4000	98.2000
1		136	67.3706	14.4640	1.2403	21.0000	100.0
Diff (1-2)	Pooled		1.4388	14.2390	1.9688		
Diff (1-2)	Satterthwaite		1.4388		1.9497		

status2	Method	Mean	95% CL Mean		Std Dev	95% CL Std Dev	
0		68.8094	65.8178	71.8011	13.8698	12.0525	16.3377
1		67.3706	64.9177	69.8235	14.4640	12.9253	16.4219
Diff (1-2)	Pooled	1.4388	-2.4414	5.3190	14.2390	13.0212	15.7101
Diff (1-2)	Satterthwaite	1.4388	-2.4079	5.2855			

Method	Variances	DF	t Value	Pr > |t|
Pooled	Equal	219	0.73	0.4657
Satterthwaite	Unequal	184.08	0.74	0.4615

Equality of Variances				
Method	Num DF	Den DF	F Value	Pr > F
Folded F	135	84	1.09	0.6824

Lavie National survey
Significance testing for admit likelihood

The TTEST Procedure

Variable: avgabscon5

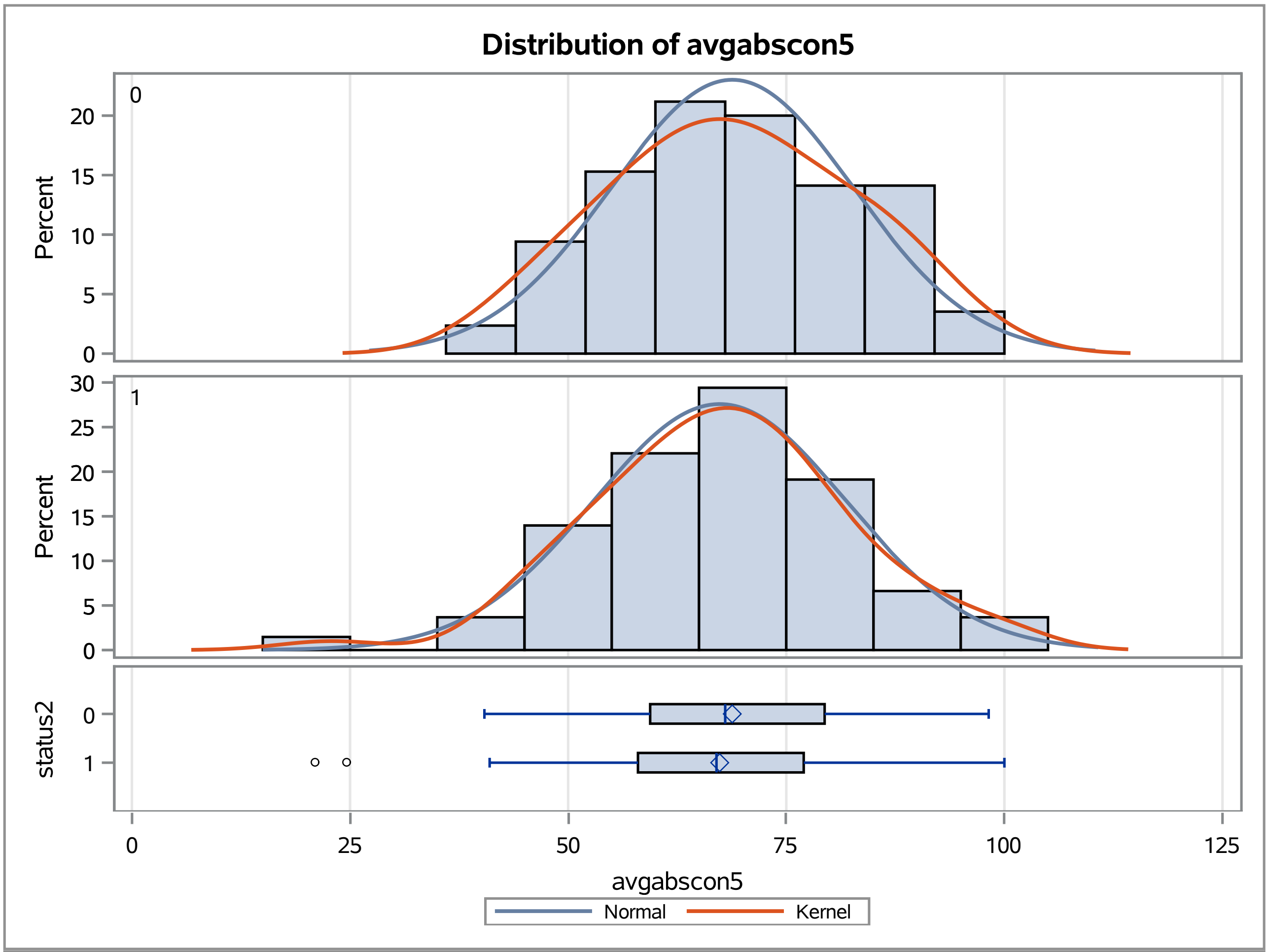

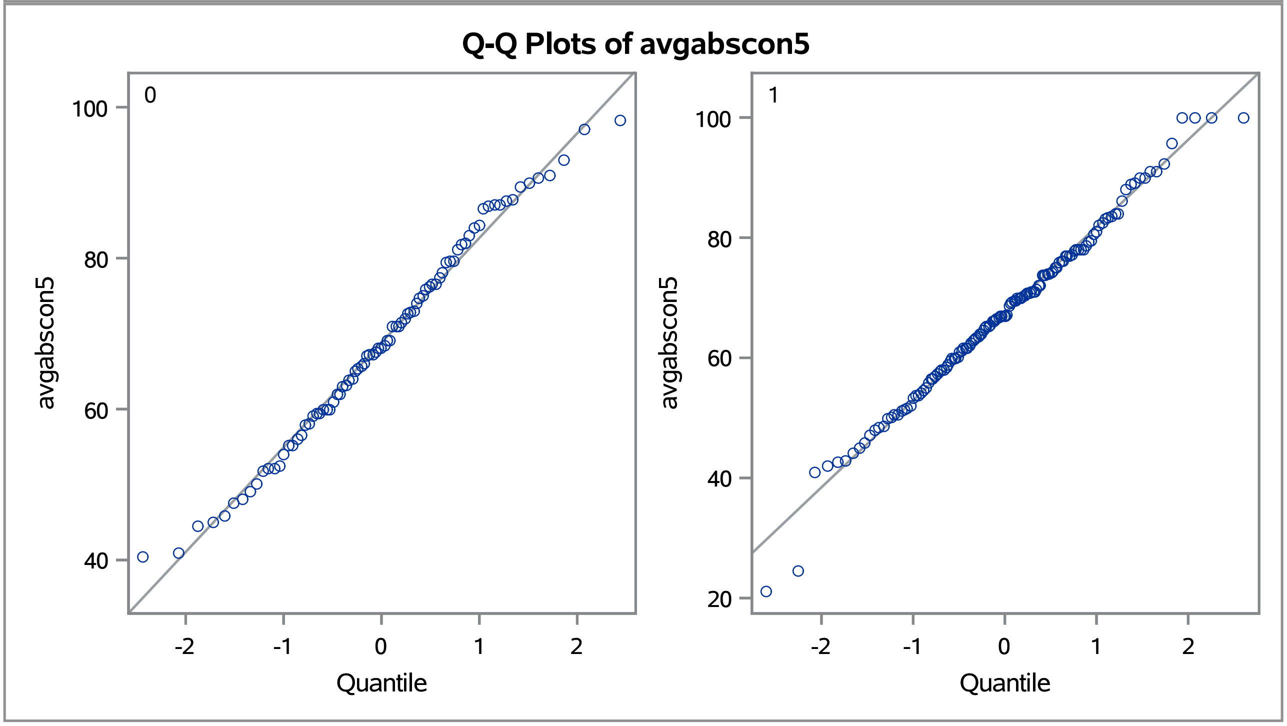

Lavie National survey
Significance testing for admit likelihood

The Mixed Procedure

Model Information	
Data Set	WORK.NAT4B
Dependent Variable	avgabscon5
Covariance Structure	Diagonal
Estimation Method	REML
Residual Variance Method	Profile
Fixed Effects SE Method	Model-Based
Degrees of Freedom Method	Residual

Class Level Information		
Class	Levels	Values
Site_region	5	1 2 3 4 5

Dimensions	
Covariance Parameters	1
Columns in X	6
Columns in Z	0
Subjects	1
Max Obs per Subject	223

Number of Observations	
Number of Observations Read	246
Number of Observations Used	223
Number of Observations Not Used	23

Covariance Parameter Estimates	
Cov Parm	Estimate
Residual	197.83

Fit Statistics	
-2 Res Log Likelihood	1789.6
AIC (Smaller is Better)	1791.6
AICC (Smaller is Better)	1791.6
BIC (Smaller is Better)	1795.0

Lavie National survey
Significance testing for admit likelihood

The Mixed Procedure

Type 3 Tests of Fixed Effects				
**Effect**	**Num DF**	**Den DF**	**F Value**	**Pr > F**
**Site_region**	4	218	2.16	0.0742

Least Squares Means						
**Effect**	**Site_region**	**Estimate**	**Standard Error**	**DF**	**t Value**	**Pr > |t|**
**Site_region**	1	71.0000	2.3442	218	30.29	<.0001
**Site_region**	2	70.0889	3.3152	218	21.14	<.0001
**Site_region**	3	62.8812	2.4864	218	25.29	<.0001
**Site_region**	4	69.5033	1.4744	218	47.14	<.0001
**Site_region**	5	65.6000	2.0738	218	31.63	<.0001

Differences of Least Squares Means							
**Effect**	**Site_region**	**Site_region**	**Estimate**	**Standard Error**	**DF**	**t Value**	**Pr > |t|**
**Site_region**	1	2	0.9111	4.0603	218	0.22	0.8227
**Site_region**	1	3	8.1188	3.4172	218	2.38	0.0184
**Site_region**	1	4	1.4967	2.7694	218	0.54	0.5894
**Site_region**	1	5	5.4000	3.1299	218	1.73	0.0859
**Site_region**	2	3	7.2076	4.1440	218	1.74	0.0834
**Site_region**	2	4	0.5856	3.6283	218	0.16	0.8719
**Site_region**	2	5	4.4889	3.9104	218	1.15	0.2523
**Site_region**	3	4	-6.6220	2.8907	218	-2.29	0.0229
**Site_region**	3	5	-2.7187	3.2377	218	-0.84	0.4020
**Site_region**	4	5	3.9033	2.5445	218	1.53	0.1265

Lavie National survey
Significance testing for admit likelihood predictors for attendings only

The TTEST Procedure

Variable: avgabscon5

er_exp	Method	N	Mean	Std Dev	Std Err	Minimum	Maximum
0		46	67.5391	14.4269	2.1271	40.4000	97.0000
1		39	70.3077	13.2102	2.1153	44.4000	98.2000
Diff (1-2)	Pooled		-2.7686	13.8831	3.0219		
Diff (1-2)	Satterthwaite		-2.7686		2.9999		

er_exp	Method	Mean	95% CL Mean		Std Dev	95% CL Std Dev	
0		67.5391	63.2549	71.8234	14.4269	11.9662	18.1711
1		70.3077	66.0254	74.5899	13.2102	10.7960	17.0250
Diff (1-2)	Pooled	-2.7686	-8.7791	3.2419	13.8831	12.0546	16.3708
Diff (1-2)	Satterthwaite	-2.7686	-8.7358	3.1986			

Method	Variances	DF	t Value	Pr > |t|
Pooled	Equal	83	-0.92	0.3622
Satterthwaite	Unequal	82.485	-0.92	0.3588

Equality of Variances				
Method	Num DF	Den DF	F Value	Pr > F
Folded F	45	38	1.19	0.5812

Lavie National survey
Significance testing for admit likelihood predictors for attendings only

The TTEST Procedure

Variable: avgabscon5

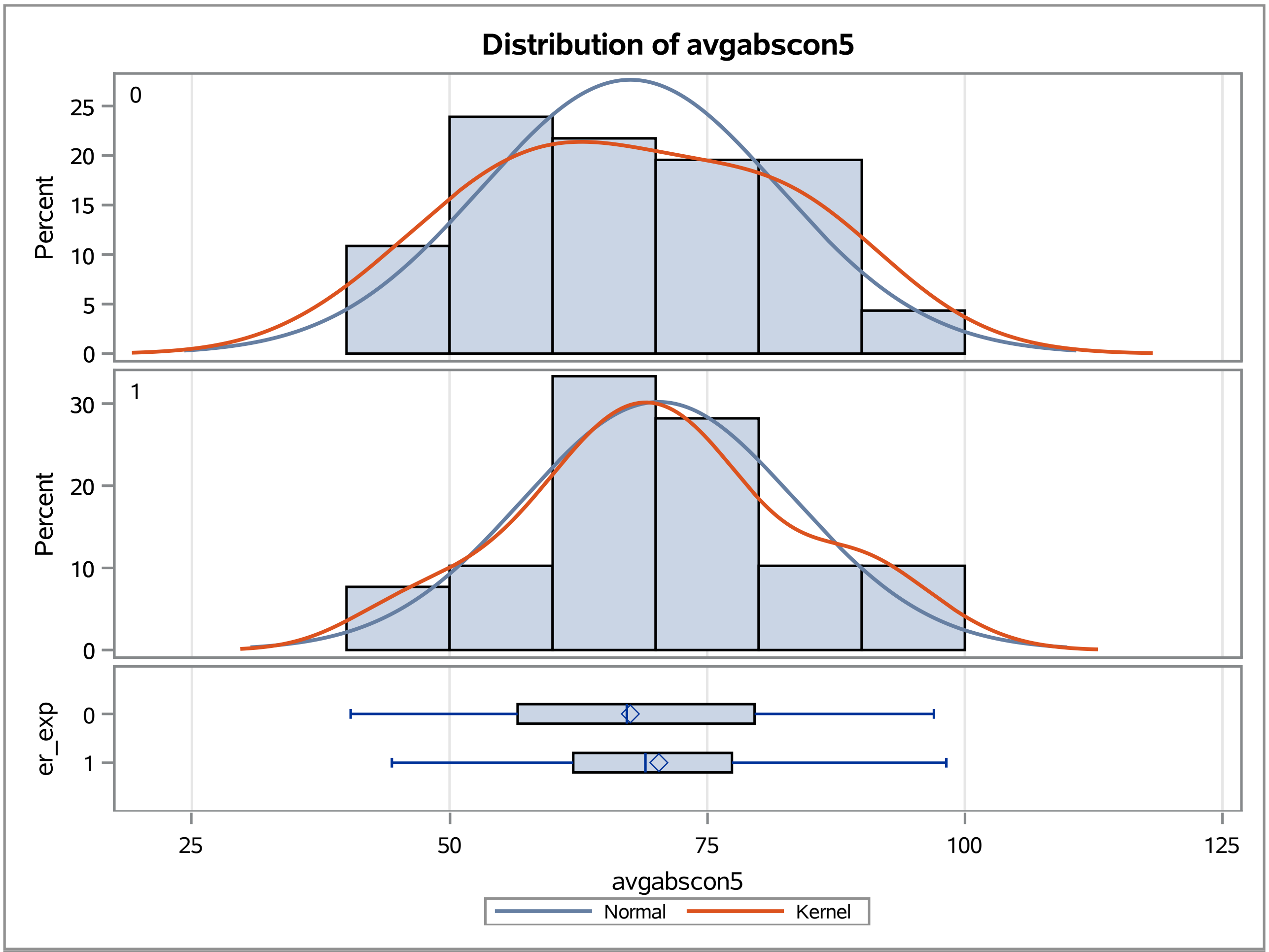

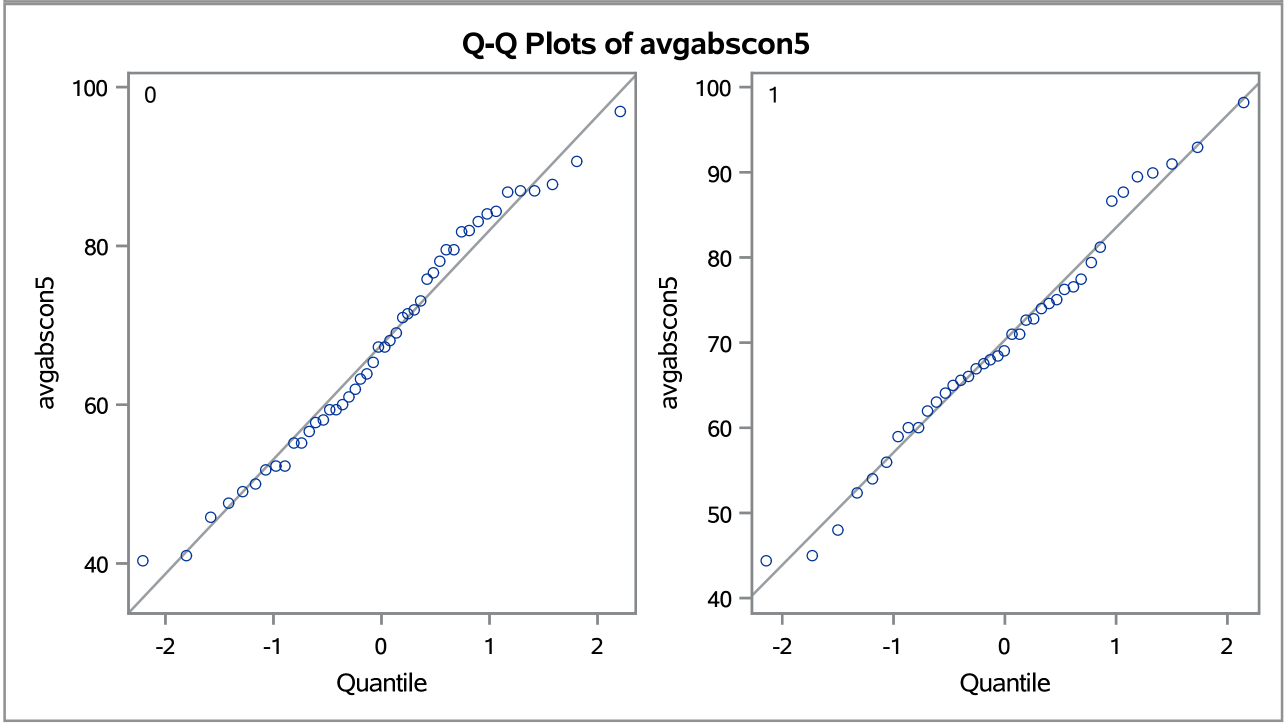

Lavie National survey
Significance testing for admit likelihood predictors for attendings only

The TTEST Procedure

Variable: avgabscon5

EPS	Method	N	Mean	Std Dev	Std Err	Minimum	Maximum
0		57	67.6140	14.3706	1.9034	40.4000	98.2000
1		28	71.2429	12.6888	2.3980	48.0000	93.0000
Diff (1-2)	Pooled		-3.6288	13.8460	3.1953		
Diff (1-2)	Satterthwaite		-3.6288		3.0616		

EPS	Method	Mean	95% CL Mean		Std Dev	95% CL Std Dev	
0		67.6140	63.8010	71.4271	14.3706	12.1325	17.6291
1		71.2429	66.3227	76.1631	12.6888	10.0320	17.2712
Diff (1-2)	Pooled	-3.6288	-9.9842	2.7266	13.8460	12.0223	16.3270
Diff (1-2)	Satterthwaite	-3.6288	-9.7524	2.4948			

Method	Variances	DF	t Value	Pr > |t|
Pooled	Equal	83	-1.14	0.2594
Satterthwaite	Unequal	60.217	-1.19	0.2406

Equality of Variances				
Method	Num DF	Den DF	F Value	Pr > F
Folded F	56	27	1.28	0.4862

Lavie National survey
Significance testing for admit likelihood predictors for attendings only

The TTEST Procedure

Variable: avgabscon5

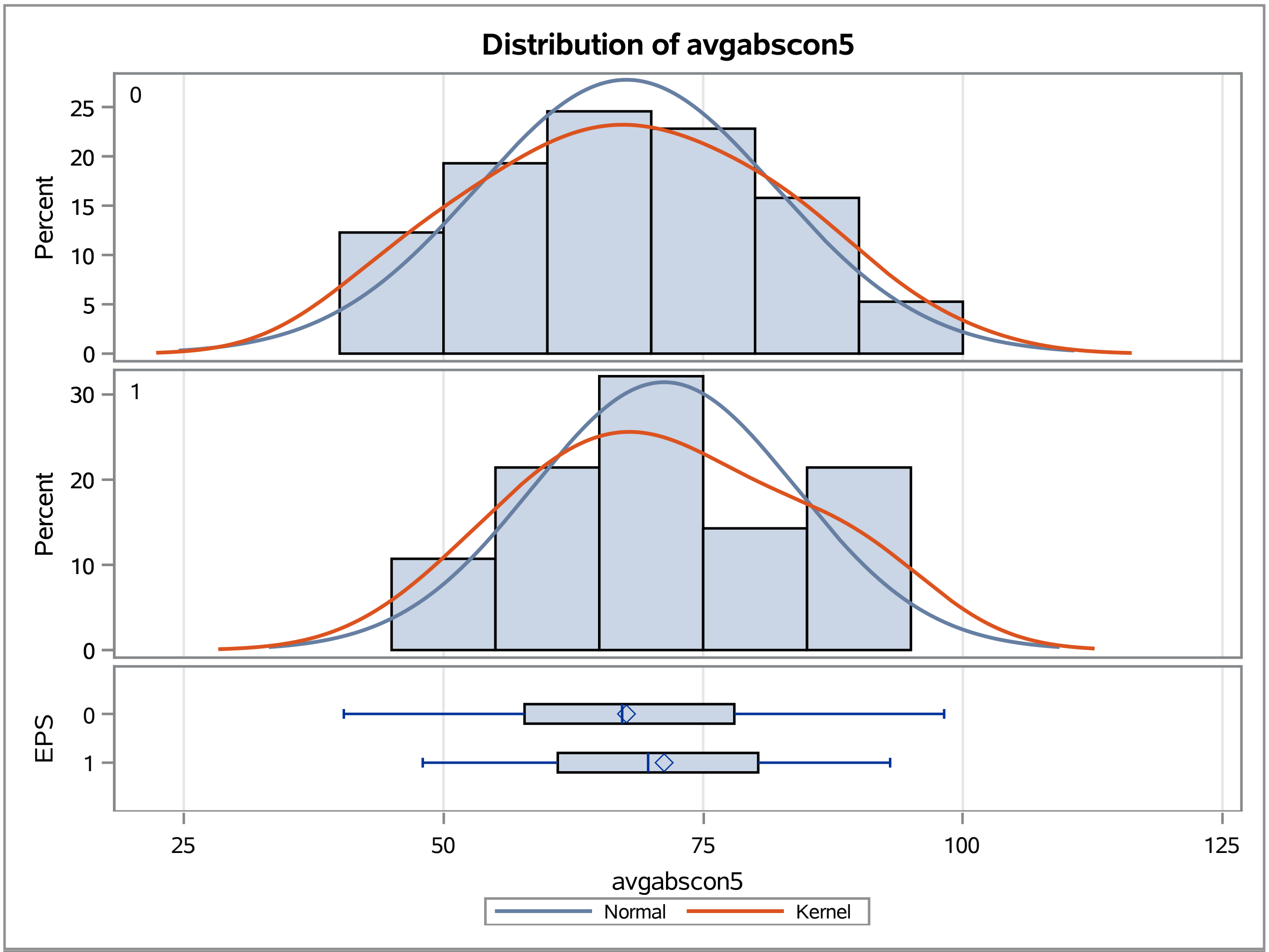

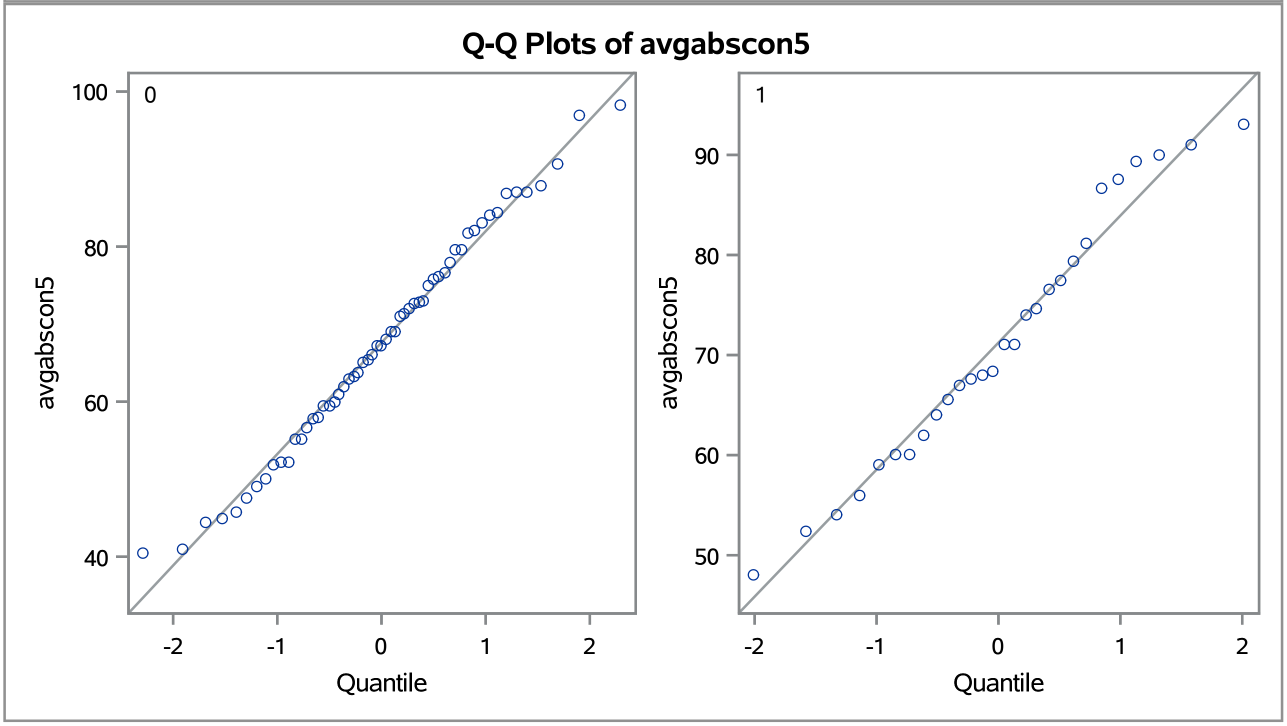

Lavie National survey
Significance testing for admit likelihood predictors for attendings only

The TTEST Procedure

Variable: avgabscon5

inpx2	Method	N	Mean	Std Dev	Std Err	Minimum	Maximum
0		49	67.1673	13.7611	1.9659	40.4000	90.6000
1		36	71.0444	13.8951	2.3159	44.4000	98.2000
Diff (1-2)	Pooled		-3.8771	13.8178	3.0332		
Diff (1-2)	Satterthwaite		-3.8771		3.0377		

inpx2	Method	Mean	95% CL Mean		Std Dev	95% CL Std Dev	
0		67.1673	63.2147	71.1200	13.7611	11.4757	17.1918
1		71.0444	66.3430	75.7459	13.8951	11.2701	18.1253
Diff (1-2)	Pooled	-3.8771	-9.9100	2.1558	13.8178	11.9979	16.2937
Diff (1-2)	Satterthwaite	-3.8771	-9.9284	2.1742			

Method	Variances	DF	t Value	Pr > |t|
Pooled	Equal	83	-1.28	0.2047
Satterthwaite	Unequal	75.159	-1.28	0.2058

Equality of Variances				
Method	Num DF	Den DF	F Value	Pr > F
Folded F	35	48	1.02	0.9380

Lavie National survey
Significance testing for admit likelihood predictors for attendings only

The TTEST Procedure

Variable: avgabscon5

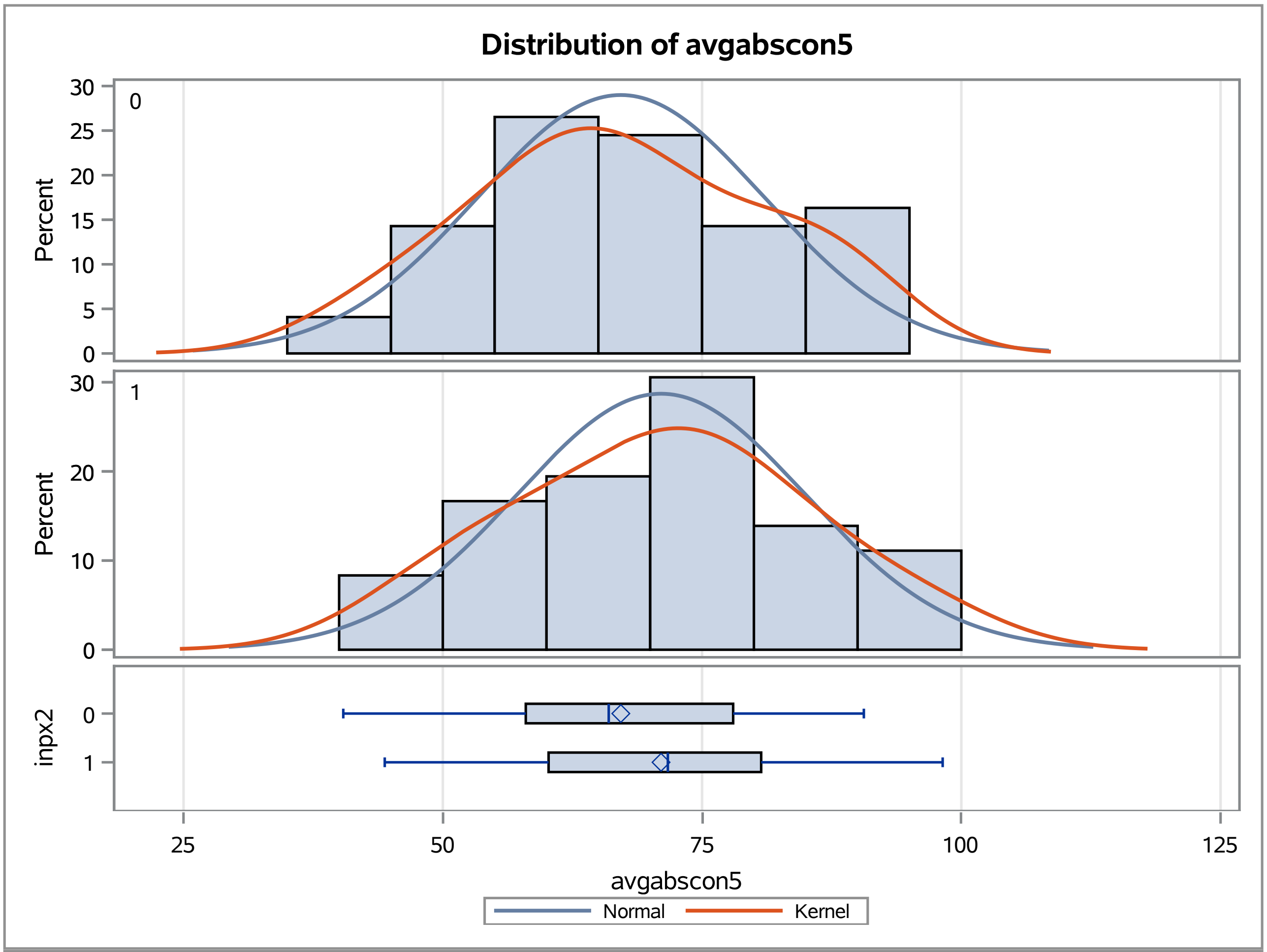

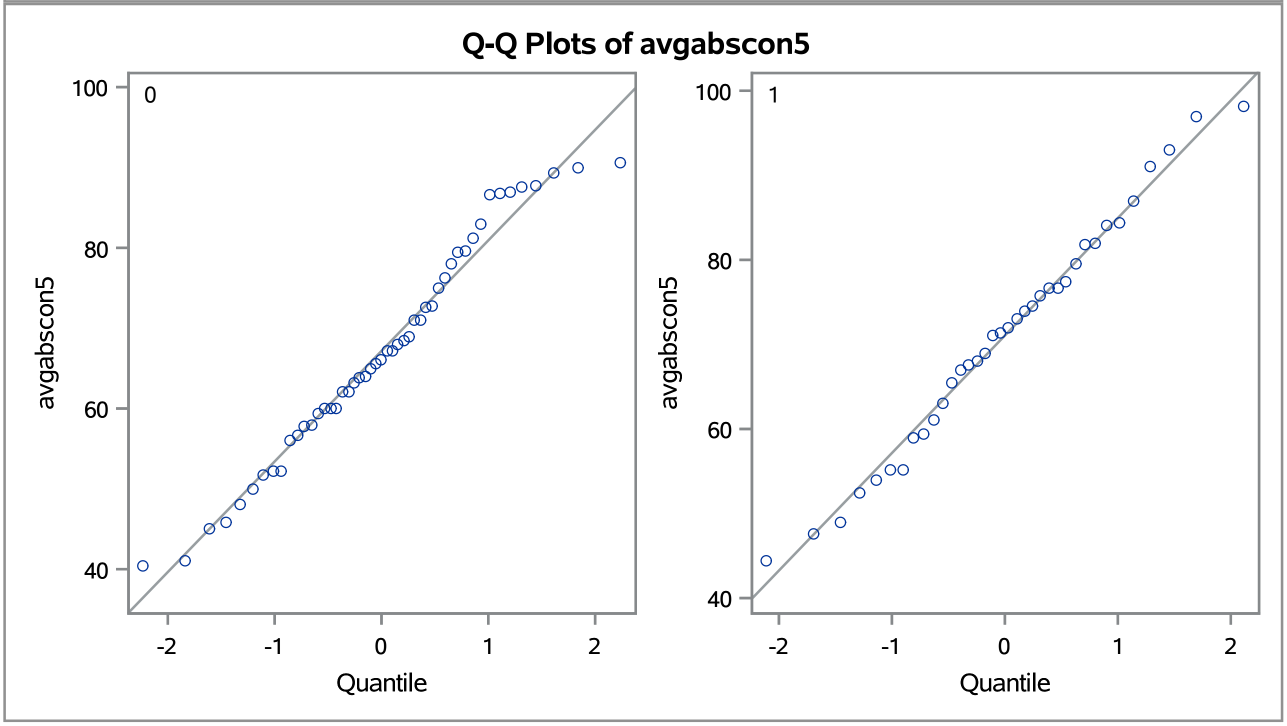

Lavie National survey
Significance testing for admit likelihood predictors for attendings only

The TTEST Procedure

Variable: avgabscon5

outpx	Method	N	Mean	Std Dev	Std Err	Minimum	Maximum
0		41	70.6488	14.3173	2.2360	41.0000	98.2000
1		44	67.0955	13.3746	2.0163	40.4000	90.6000
Diff (1-2)	Pooled		3.5533	13.8370	3.0035		
Diff (1-2)	Satterthwaite		3.5533		3.0108		

outpx	Method	Mean	95% CL Mean		Std Dev	95% CL Std Dev	
0		70.6488	66.1297	75.1679	14.3173	11.7547	18.3190
1		67.0955	63.0292	71.1617	13.3746	11.0504	16.9460
Diff (1-2)	Pooled	3.5533	-2.4206	9.5272	13.8370	12.0145	16.3163
Diff (1-2)	Satterthwaite	3.5533	-2.4368	9.5435			

Method	Variances	DF	t Value	Pr > |t|
Pooled	Equal	83	1.18	0.2402
Satterthwaite	Unequal	81.42	1.18	0.2414

Equality of Variances				
Method	Num DF	Den DF	F Value	Pr > F
Folded F	40	43	1.15	0.6601

Lavie National survey
Significance testing for admit likelihood predictors for attendings only

The TTEST Procedure

Variable: avgabscon5

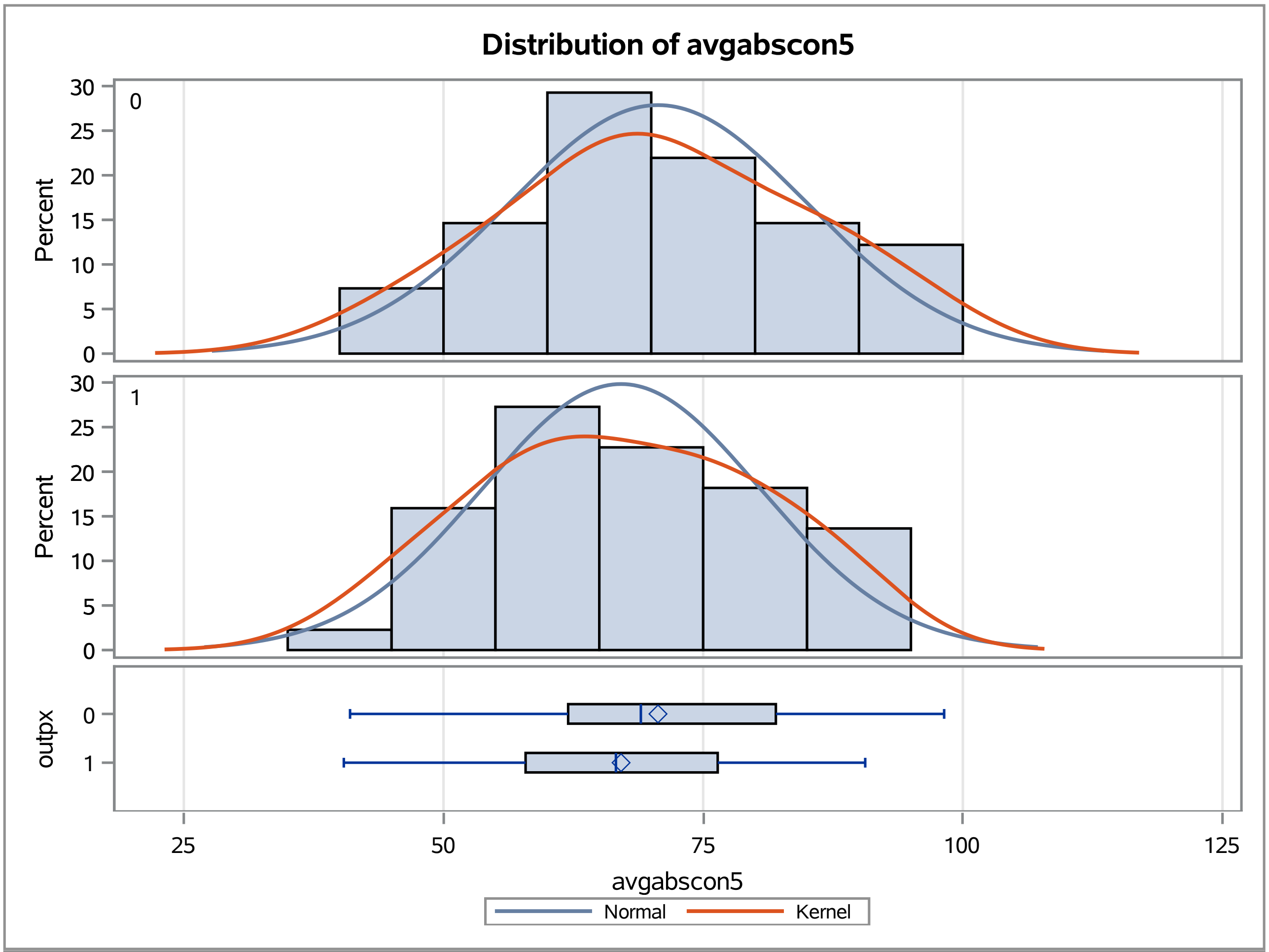

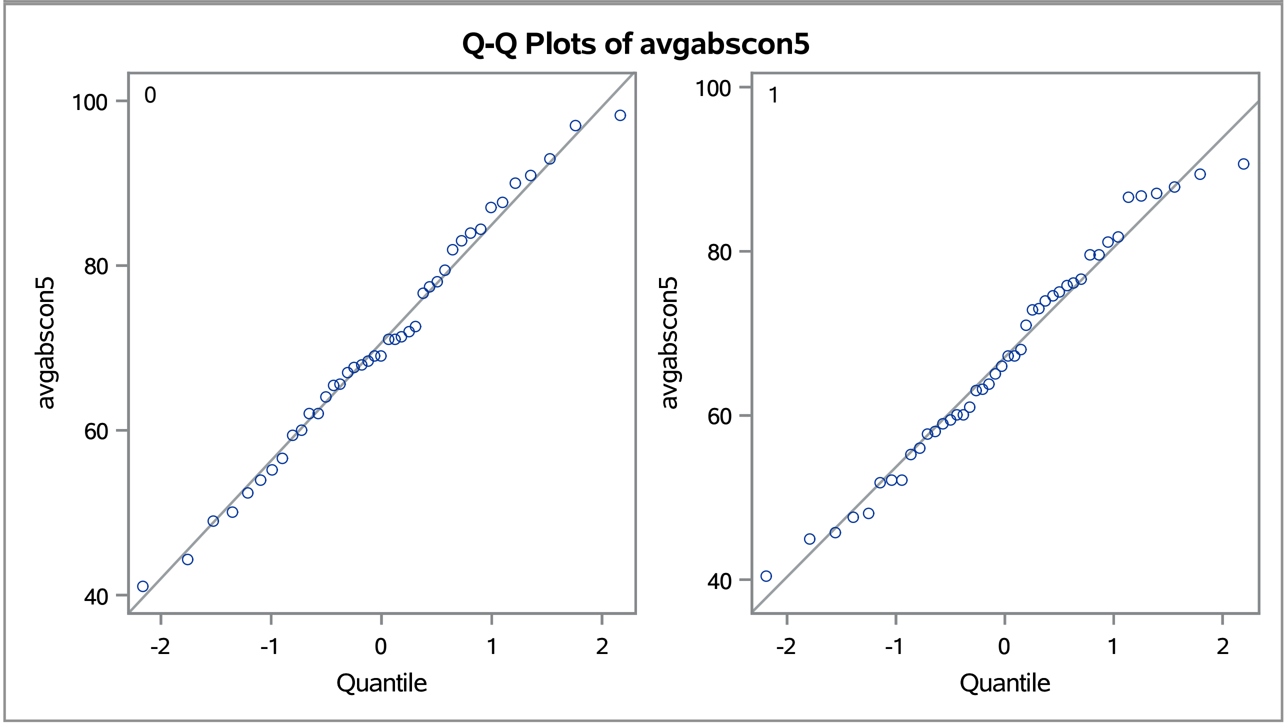

Lavie National survey
Significance testing for admit likelihood predictors for attendings only

The TTEST Procedure

Variable: avgabscon5

pes	Method	N	Mean	Std Dev	Std Err	Minimum	Maximum
0		20	69.3700	15.6531	3.5001	44.4000	98.2000
1		18	71.4222	10.6669	2.5142	56.0000	91.0000
Diff (1-2)	Pooled		-2.0522	13.5295	4.3956		
Diff (1-2)	Satterthwaite		-2.0522		4.3096		

pes	Method	Mean	95% CL Mean		Std Dev	95% CL Std Dev	
0		69.3700	62.0441	76.6959	15.6531	11.9040	22.8625
1		71.4222	66.1177	76.7268	10.6669	8.0043	15.9912
Diff (1-2)	Pooled	-2.0522	-10.9670	6.8625	13.5295	11.0023	17.5743
Diff (1-2)	Satterthwaite	-2.0522	-10.8136	6.7092			

Method	Variances	DF	t Value	Pr > |t|
Pooled	Equal	36	-0.47	0.6434
Satterthwaite	Unequal	33.652	-0.48	0.6370

Equality of Variances				
Method	Num DF	Den DF	F Value	Pr > F
Folded F	19	17	2.15	0.1178

Lavie National survey
Significance testing for admit likelihood predictors for attendings only

The TTEST Procedure

Variable: avgabscon5

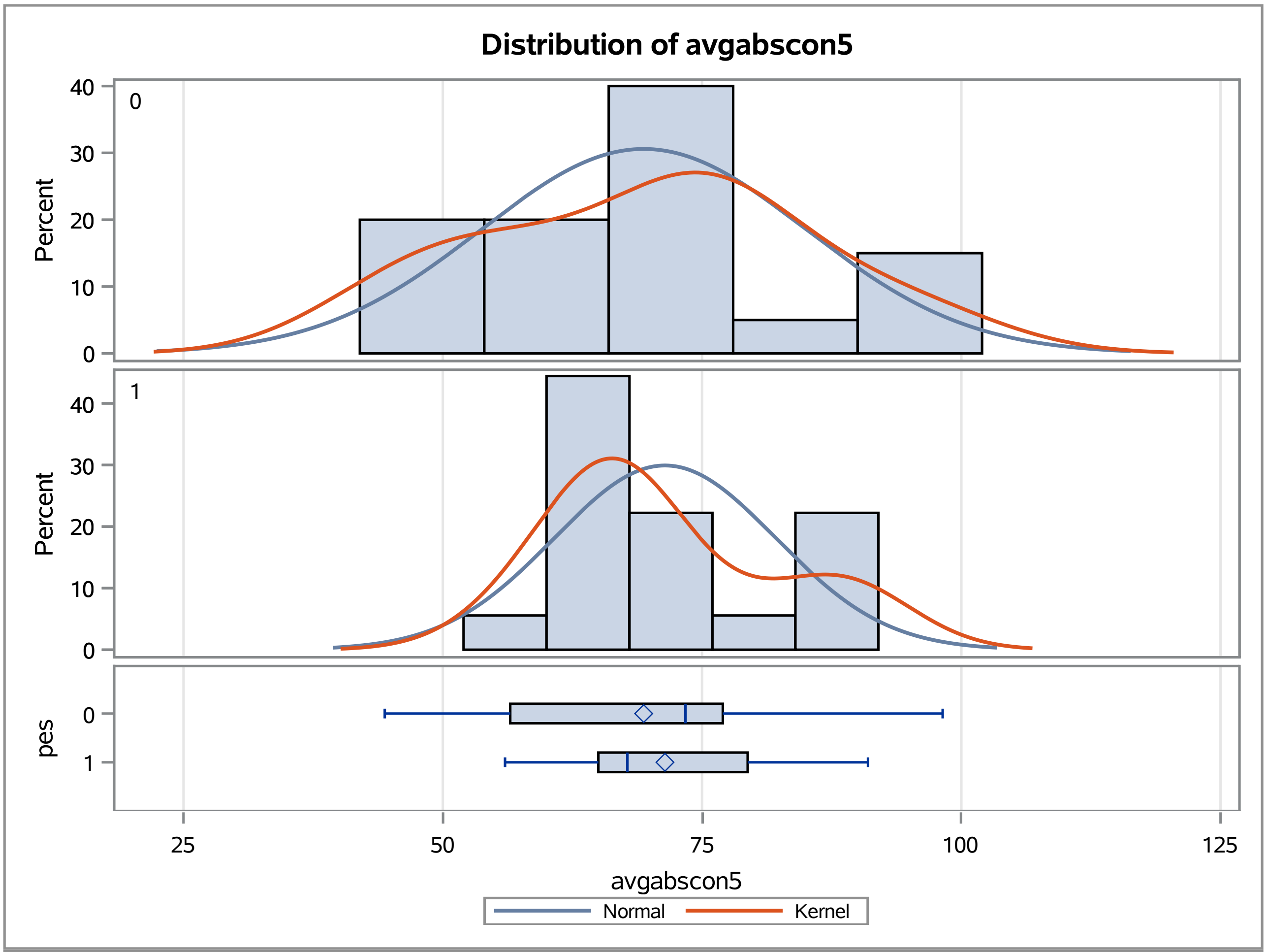

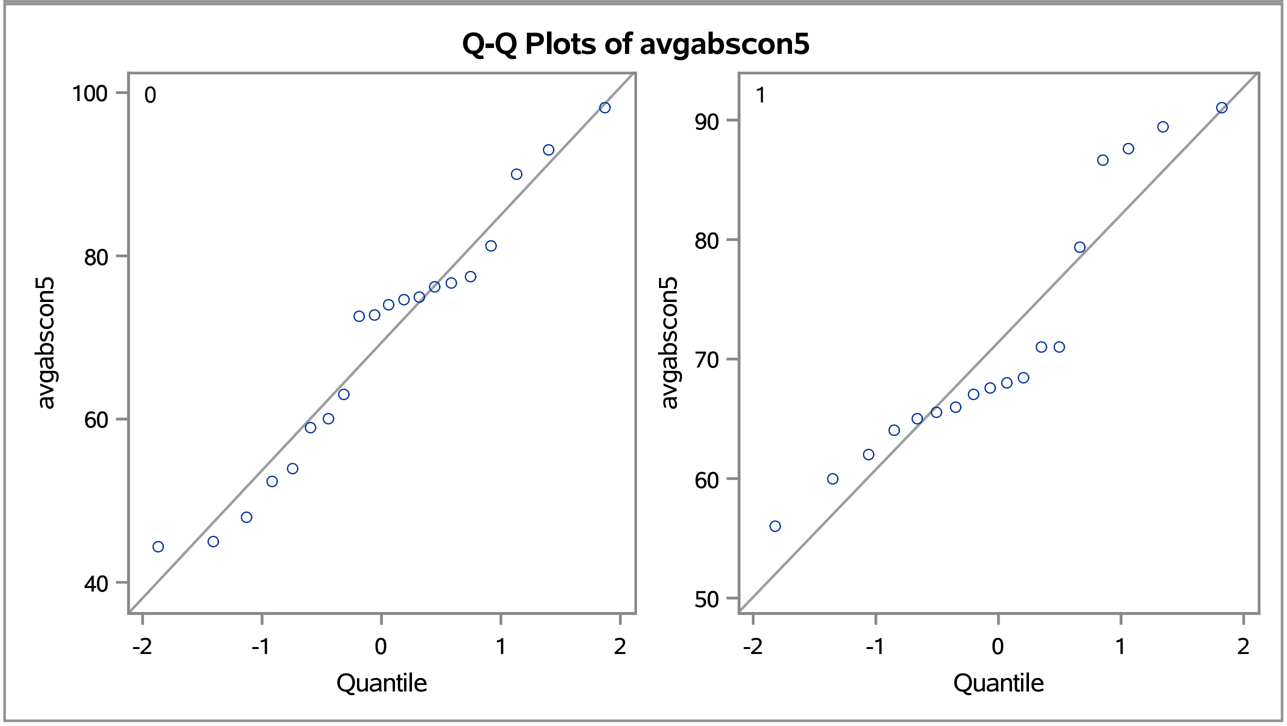

Lavie National survey
Significance testing for admit likelihood predictors for attendings only

The TTEST Procedure

Variable: avgabscon5

training_pes	Method	N	Mean	Std Dev	Std Err	Minimum	Maximum
**0**		39	66.1128	16.1195	2.5812	40.4000	98.2000
**1**		46	71.0957	11.3150	1.6683	48.0000	91.0000
**Diff (1-2)**	**Pooled**		-4.9828	13.7250	2.9875		
**Diff (1-2)**	**Satterthwaite**		-4.9828		3.0734		

training_pes	Method	Mean	95% CL Mean		Std Dev	95% CL Std Dev	
**0**		66.1128	60.8875	71.3382	16.1195	13.1736	20.7745
**1**		71.0957	67.7355	74.4558	11.3150	9.3851	14.2515
**Diff (1-2)**	**Pooled**	-4.9828	-10.9249	0.9592	13.7250	11.9173	16.1843
**Diff (1-2)**	**Satterthwaite**	-4.9828	-11.1181	1.1524			

Method	Variances	DF	t Value	Pr > |t|
**Pooled**	Equal	83	-1.67	0.0991
**Satterthwaite**	Unequal	66.57	-1.62	0.1097

Equality of Variances				
**Method**	**Num DF**	**Den DF**	**F Value**	**Pr > F**
**Folded F**	38	45	2.03	0.0235

Lavie National survey
Significance testing for admit likelihood predictors for attendings only

The TTEST Procedure

Variable: avgabscon5

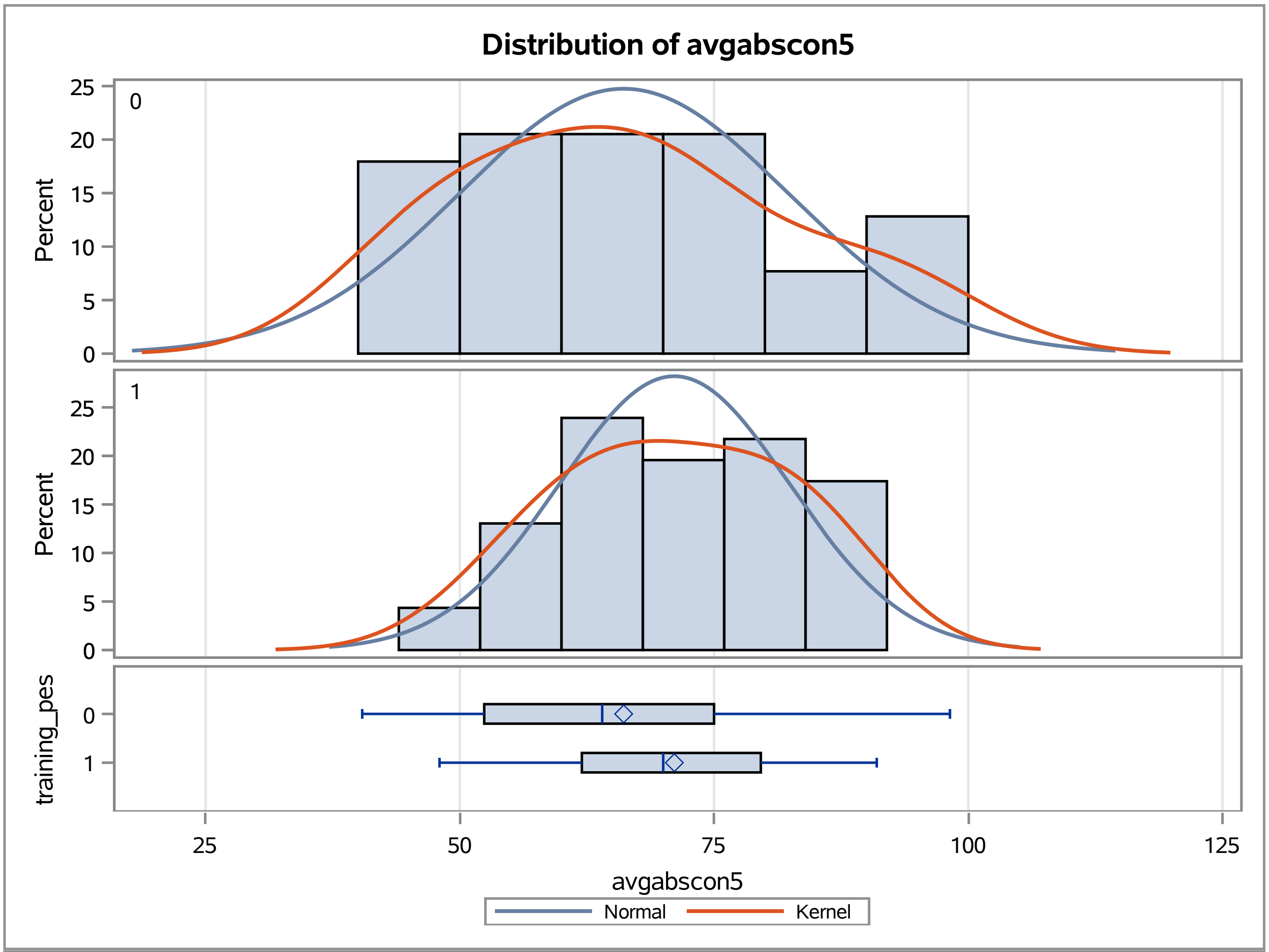

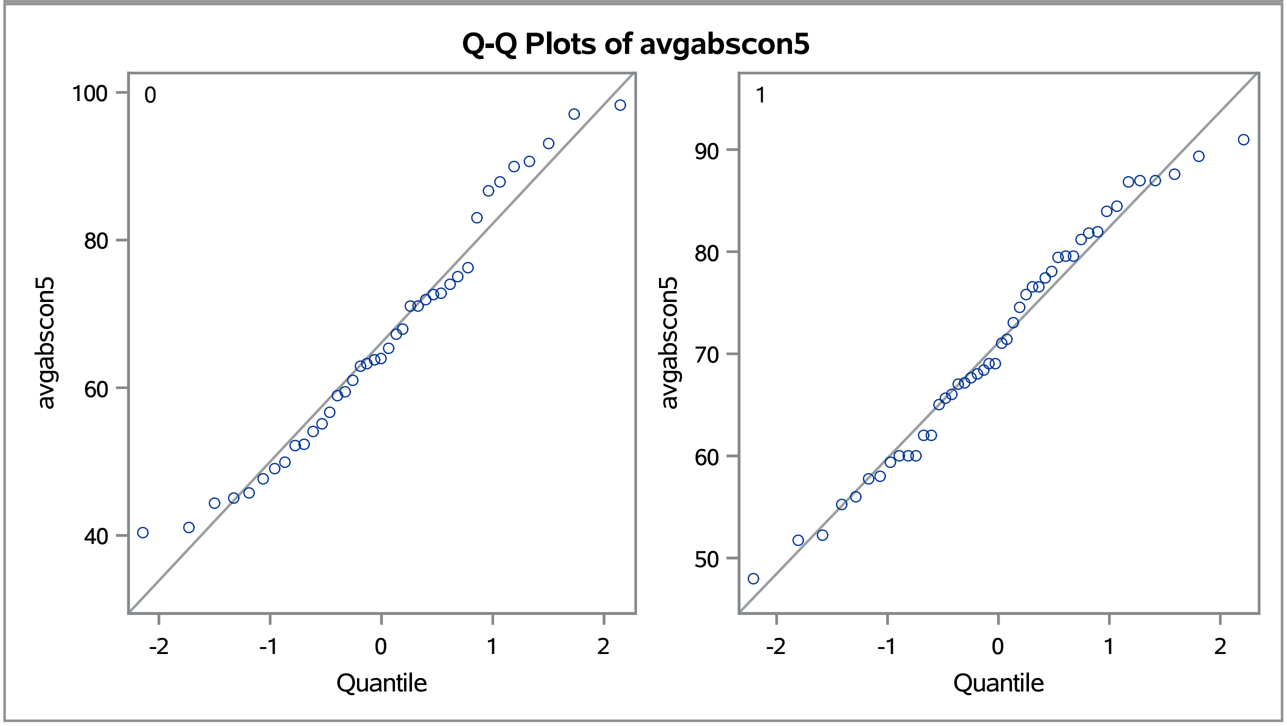

Lavie National survey
Significance testing for admit likelihood predictors for attendings only

The CORR Procedure

**2 With Variables:**	yearspracticing percent_er
**1 Variables:**	avgabscon5

Simple Statistics							
**Variable**	**N**	**Mean**	**Std Dev**	**Sum**	**Minimum**	**Maximum**	**Label**
**yearspracticing**	96	2.58333	1.86754	248.00000	1.00000	9.00000	yearspracticing
**percent_er**	96	2.16667	1.63299	208.00000	1.00000	6.00000	percent_er
**avgabscon5**	85	68.80941	13.86983	5849	40.40000	98.20000	

Pearson Correlation Coefficients<br>Prob > |r| under H0: Rho=0<br>Number of Observations	
	**avgabscon5**
**yearspracticing**<br>**yearspracticing**	0.08791<br>0.4237<br>85
**percent_er**<br>**percent_er**	0.21775<br>0.0453<br>85

Lavie National survey
Significance testing for admit likelihood predictors for trainees only

The CORR Procedure

**3 With Variables:**	PGY pgy2 time_er
**1 Variables:**	avgabscon5

Simple Statistics							
**Variable**	**N**	**Mean**	**Std Dev**	**Sum**	**Minimum**	**Maximum**	**Label**
**PGY**	147	2.51701	1.20133	370.00000	1.00000	5.00000	PGY
**pgy2**	147	2.46259	1.10585	362.00000	1.00000	4.00000	
**time_er**	147	3.04082	0.78407	447.00000	1.00000	4.00000	time_er
**avgabscon5**	136	67.37059	14.46402	9162	21.00000	100.00000	

Pearson Correlation Coefficients<br>Prob > |r| under H0: Rho=0<br>Number of Observations	
	**avgabscon5**
**PGY**<br>**PGY**	0.32323<br>0.0001<br>136
**pgy2**	0.35205<br><.0001<br>136
**time_er**<br>**time_er**	0.15854<br>0.0653<br>136

Lavie National survey
Significance testing for admit likelihood
full sample (attendings + trainess) personality-related analyses

The CORR Procedure

**7 With Variables:**	paternalism worry_px use_laws risk_comfort ih_benefits perception_self_admit perception_inst_admit
**1 Variables:**	avgabscon5

Simple Statistics							
**Variable**	**N**	**Mean**	**Std Dev**	**Sum**	**Minimum**	**Maximum**	**Label**
**paternalism**	240	2.69083	0.66893	645.80000	1.00000	4.40000	
**worry_px**	240	2.14167	0.50596	514.00000	1.00000	4.00000	worry_px
**use_laws**	241	3.08714	0.83459	744.00000	1.00000	4.00000	use_laws
**risk_comfort**	241	2.95436	0.62081	712.00000	1.00000	4.00000	risk_comfort
**ih_benefits**	241	2.56846	0.58138	619.00000	2.00000	4.00000	ih_benefits
**perception_self_admit**	241	2.02075	0.57335	487.00000	1.00000	3.00000	perception_self_admit
**perception_inst_admit**	240	1.84167	0.62709	442.00000	1.00000	3.00000	perception_inst_admit
**avgabscon5**	223	68.03677	14.21183	15172	21.00000	100.00000	

Pearson Correlation Coefficients<br>Prob > |r| under H0: Rho=0<br>Number of Observations	
	**avgabscon5**
**paternalism**	0.15461<br>0.0224<br>218
**worry_px**<br>**worry_px**	-0.14974<br>0.0271<br>218
**use_laws**<br>**use_laws**	0.07863<br>0.2465<br>219
**risk_comfort**<br>**risk_comfort**	0.38768<br><.0001<br>219
**ih_benefits**<br>**ih_benefits**	0.15644<br>0.0205<br>219
**perception_self_admit**<br>**perception_self_admit**	-0.03522<br>0.6042<br>219
**perception_inst_admit**<br>**perception_inst_admit**	0.06436<br>0.3431<br>219

Lavie National survey: correlations with 95% CI

The CORR Procedure

**7 With Variables:**	paternalism worry_px use_laws risk_comfort ih_benefits perception_self_admit perception_inst_admit
**2 Variables:**	admit5 avgabscon5

Simple Statistics

Variable	N	Mean	Std Dev	Sum	Minimum	Maximum	Label
**paternalism**	240	2.69083	0.66893	645.80000	1.00000	4.40000	
**worry_px**	240	2.14167	0.50596	514.00000	1.00000	4.00000	worry_px
**use_laws**	241	3.08714	0.83459	744.00000	1.00000	4.00000	use_laws
**risk_comfort**	241	2.95436	0.62081	712.00000	1.00000	4.00000	risk_comfort
**ih_benefits**	241	2.56846	0.58138	619.00000	2.00000	4.00000	ih_benefits
**perception_self_admit**	241	2.02075	0.57335	487.00000	1.00000	3.00000	perception_self_admit
**perception_inst_admit**	240	1.84167	0.62709	442.00000	1.00000	3.00000	perception_inst_admit
**admit5**	246	0.47561	0.24573	117.00000	0	1.00000	
**avgabscon5**	223	68.03677	14.21183	15172	21.00000	100.00000	

Pearson Correlation Coefficients
Prob > |r| under H0: Rho=0
Number of Observations

	admit5	avgabscon5
**paternalism**	0.14787<br>0.0219<br>240	0.15461<br>0.0224<br>218
**worry_px**<br>worry_px	0.08752<br>0.1766<br>240	-0.14974<br>0.0271<br>218
**use_laws**<br>use_laws	-0.04451<br>0.4916<br>241	0.07863<br>0.2465<br>219
**risk_comfort**<br>risk_comfort	0.03917<br>0.5451<br>241	0.38768<br><.0001<br>219
**ih_benefits**<br>ih_benefits	0.18867<br>0.0033<br>241	0.15644<br>0.0205<br>219
**perception_self_admit**<br>perception_self_admit	-0.21276<br>0.0009<br>241	-0.03522<br>0.6042<br>219
**perception_inst_admit**<br>perception_inst_admit	-0.15081<br>0.0194<br>240	0.06436<br>0.3431<br>219

Lavie National survey: correlations with 95% CI

The CORR Procedure

Pearson Correlation Statistics (Fisher's z Transformation)

Variable	With Variable	N	Sample Correlation	Fisher's z	Bias Adjustment	Correlation Estimate	95% Confidence Limits		p Value for H0:Rho=0
admit5	paternalism	240	0.14787	0.14897	0.0003094	0.14757	0.021340	0.269171	0.0218
avgabscon5	paternalism	218	0.15461	0.15586	0.0003562	0.15426	0.021833	0.281373	0.0223
admit5	worry_px	240	0.08752	0.08775	0.0001831	0.08734	-0.039727	0.211631	0.1767
avgabscon5	worry_px	218	-0.14974	-0.15088	-0.0003450	-0.14940	-0.276787	-0.016860	0.0269
admit5	use_laws	241	-0.04451	-0.04454	-0.0000927	-0.04442	-0.169833	0.082409	0.4920
avgabscon5	use_laws	219	0.07863	0.07879	0.0001803	0.07845	-0.054691	0.208853	0.2469
admit5	risk_comfort	241	0.03917	0.03919	0.0000816	0.03909	-0.087714	0.164639	0.5455
avgabscon5	risk_comfort	219	0.38768	0.40906	0.0008892	0.38692	0.268100	0.494147	<.0001
admit5	ih_benefits	241	0.18867	0.19095	0.0003931	0.18829	0.063430	0.307341	0.0032
avgabscon5	ih_benefits	219	0.15644	0.15774	0.0003588	0.15609	0.024016	0.282813	0.0204
admit5	perception_self_admit	241	-0.21276	-0.21606	-0.0004432	-0.21233	-0.329850	-0.088338	0.0009
avgabscon5	perception_self_admit	219	-0.03522	-0.03523	-0.0000808	-0.03513	-0.166931	0.097895	0.6046
admit5	perception_inst_admit	240	-0.15081	-0.15197	-0.0003155	-0.15050	-0.271951	-0.024337	0.0193
avgabscon5	perception_inst_admit	219	0.06436	0.06445	0.0001476	0.06422	-0.068943	0.195130	0.3435

The CORR Procedure

status2=.

**7 With Variables:**	paternalism worry_px use_laws risk_comfort ih_benefits perception_self_admit perception_inst_admit
**2 Variables:**	admit5 avgabscon5

Simple Statistics

Variable	N	Mean	Std Dev	Sum	Minimum	Maximum	Label
**paternalism**	0	.	.	.	.	.	
**worry_px**	0	.	.	.	.	.	worry_px
**use_laws**	0	.	.	.	.	.	use_laws
**risk_comfort**	0	.	.	.	.	.	risk_comfort
**ih_benefits**	0	.	.	.	.	.	ih_benefits
**perception_self_admit**	0	.	.	.	.	.	perception_self_admit
**perception_inst_admit**	0	.	.	.	.	.	perception_inst_admit
**admit5**	3	0.46667	0.41633	1.40000	0	0.80000	
**avgabscon5**	2	80.50000	3.81838	161.00000	77.80000	83.20000	

Pearson Correlation Coefficients
Prob > |r| under H0: Rho=0
Number of Observations

	admit5	avgabscon5
**paternalism**	.<br>.<br>0	.<br>.<br>0
**worry_px**<br>worry_px	.<br>.<br>0	.<br>.<br>0
**use_laws**<br>use_laws	.<br>.<br>0	.<br>.<br>0
**risk_comfort**<br>risk_comfort	.<br>.<br>0	.<br>.<br>0
**ih_benefits**<br>ih_benefits	.<br>.<br>0	.<br>.<br>0
**perception_self_admit**<br>perception_self_admit	.<br>.<br>0	.<br>.<br>0
**perception_inst_admit**<br>perception_inst_admit	.<br>.<br>0	.<br>.<br>0

The CORR Procedure

status2=.

Pearson Correlation Statistics (Fisher's z Transformation)									
Variable	With Variable	N	Sample Correlation	Fisher's z	Bias Adjustment	Correlation Estimate	95% Confidence Limits		p Value for H0:Rho=0
admit5	paternalism	0	.	.	.	.	.	.	.
avgabscon5	paternalism	0	.	.	.	.	.	.	.
admit5	worry_px	0	.	.	.	.	.	.	.
avgabscon5	worry_px	0	.	.	.	.	.	.	.
admit5	use_laws	0	.	.	.	.	.	.	.
avgabscon5	use_laws	0	.	.	.	.	.	.	.
admit5	risk_comfort	0	.	.	.	.	.	.	.
avgabscon5	risk_comfort	0	.	.	.	.	.	.	.
admit5	ih_benefits	0	.	.	.	.	.	.	.
avgabscon5	ih_benefits	0	.	.	.	.	.	.	.
admit5	perception_self_admit	0	.	.	.	.	.	.	.
avgabscon5	perception_self_admit	0	.	.	.	.	.	.	.
admit5	perception_inst_admit	0	.	.	.	.	.	.	.
avgabscon5	perception_inst_admit	0	.	.	.	.	.	.	.

The CORR Procedure

status2=0

**7 With Variables:**	paternalism worry_px use_laws risk_comfort ih_benefits perception_self_admit perception_inst_admit
**2 Variables:**	admit5 avgabscon5

Simple Statistics

Variable	N	Mean	Std Dev	Sum	Minimum	Maximum	Label
**paternalism**	95	2.59947	0.68279	246.95000	1.00000	4.20000	
**worry_px**	95	2.18947	0.51146	208.00000	1.00000	4.00000	worry_px
**use_laws**	95	3.25263	0.77141	309.00000	1.00000	4.00000	use_laws
**risk_comfort**	95	3.04211	0.66710	289.00000	1.00000	4.00000	risk_comfort
**ih_benefits**	95	2.61053	0.64072	248.00000	2.00000	4.00000	ih_benefits
**perception_self_admit**	95	1.93684	0.59806	184.00000	1.00000	3.00000	perception_self_admit
**perception_inst_admit**	94	1.91489	0.68249	180.00000	1.00000	3.00000	perception_inst_admit
**admit5**	96	0.47292	0.26136	45.40000	0	1.00000	
**avgabscon5**	85	68.80941	13.86983	5849	40.40000	98.20000	

Pearson Correlation Coefficients
Prob > |r| under H0: Rho=0
Number of Observations

	admit5	avgabscon5
**paternalism**	0.26027<br>0.0109<br>95	0.22734<br>0.0376<br>84
**worry_px**<br>worry_px	-0.07042<br>0.4977<br>95	-0.25550<br>0.0190<br>84
**use_laws**<br>use_laws	0.10942<br>0.2912<br>95	-0.00398<br>0.9714<br>84
**risk_comfort**<br>risk_comfort	0.22568<br>0.0279<br>95	0.39235<br>0.0002<br>84
**ih_benefits**<br>ih_benefits	0.31942<br>0.0016<br>95	0.36408<br>0.0007<br>84
**perception_self_admit**<br>perception_self_admit	-0.26914<br>0.0084<br>95	-0.00409<br>0.9705<br>84
**perception_inst_admit**<br>perception_inst_admit	-0.16971<br>0.1020<br>94	-0.04943<br>0.6552<br>84

Lavie National survey: correlations with 95% CI

The CORR Procedure

status2=0

Pearson Correlation Statistics (Fisher's z Transformation)

Variable	With Variable	N	Sample Correlation	Fisher's z	Bias Adjustment	Correlation Estimate	95% Confidence Limits		p Value for H0:Rho=0
admit5	paternalism	95	0.26027	0.26639	0.00138	0.25898	0.060595	0.437674	0.0106
avgabscon5	paternalism	84	0.22734	0.23139	0.00137	0.22604	0.012241	0.420080	0.0373
admit5	worry_px	95	-0.07042	-0.07053	-0.0003746	-0.07005	-0.267807	0.133381	0.4987
avgabscon5	worry_px	84	-0.25550	-0.26129	-0.00154	-0.25406	-0.444261	-0.041955	0.0187
admit5	use_laws	95	0.10942	0.10986	0.0005820	0.10884	-0.094778	0.303724	0.2920
avgabscon5	use_laws	84	-0.00398	-0.00398	-0.0000240	-0.00395	-0.218164	0.210620	0.9714
admit5	risk_comfort	95	0.22568	0.22963	0.00120	0.22454	0.024086	0.407635	0.0276
avgabscon5	risk_comfort	84	0.39235	0.41458	0.00236	0.39035	0.192028	0.558045	0.0002
admit5	ih_benefits	95	0.31942	0.33100	0.00170	0.31789	0.124313	0.488159	0.0015
avgabscon5	ih_benefits	84	0.36408	0.38158	0.00219	0.36217	0.160220	0.535025	0.0006
admit5	perception_self_admit	95	-0.26914	-0.27594	-0.00143	-0.26781	-0.445320	-0.070051	0.0081
avgabscon5	perception_self_admit	84	-0.00409	-0.00409	-0.0000246	-0.00407	-0.218271	0.210513	0.9706
admit5	perception_inst_admit	94	-0.16971	-0.17137	-0.0009124	-0.16883	-0.359158	0.034987	0.1021
avgabscon5	perception_inst_admit	84	-0.04943	-0.04947	-0.0002978	-0.04914	-0.260784	0.167019	0.6561

The CORR Procedure

status2=1

**7 With Variables:**	paternalism worry_px use_laws risk_comfort ih_benefits perception_self_admit perception_inst_admit
**2 Variables:**	admit5 avgabscon5

Simple Statistics							
**Variable**	**N**	**Mean**	**Std Dev**	**Sum**	**Minimum**	**Maximum**	**Label**
**paternalism**	145	2.75069	0.65516	398.85000	1.20000	4.40000	
**worry_px**	145	2.11034	0.50163	306.00000	1.00000	4.00000	worry_px
**use_laws**	146	2.97945	0.85878	435.00000	1.00000	4.00000	use_laws
**risk_comfort**	146	2.89726	0.58403	423.00000	1.00000	4.00000	risk_comfort
**ih_benefits**	146	2.54110	0.53982	371.00000	2.00000	4.00000	ih_benefits
**perception_self_admit**	146	2.07534	0.55193	303.00000	1.00000	3.00000	perception_self_admit
**perception_inst_admit**	146	1.79452	0.58625	262.00000	1.00000	3.00000	perception_inst_admit
**admit5**	147	0.47755	0.23344	70.20000	0	1.00000	
**avgabscon5**	136	67.37059	14.46402	9162	21.00000	100.00000	

Pearson Correlation Coefficients<br>Prob > |r| under H0: Rho=0<br>Number of Observations		
	**admit5**	**avgabscon5**
**paternalism**	0.06196<br>0.4591<br>145	0.12920<br>0.1368<br>134
**worry_px**<br>worry_px	0.20842<br>0.0119<br>145	-0.09703<br>0.2647<br>134
**use_laws**<br>use_laws	-0.14287<br>0.0854<br>146	0.10895<br>0.2084<br>135
**risk_comfort**<br>risk_comfort	-0.11240<br>0.1768<br>146	0.37989<br><.0001<br>135
**ih_benefits**<br>ih_benefits	0.07811<br>0.3487<br>146	0.01101<br>0.8992<br>135
**perception_self_admit**<br>perception_self_admit	-0.17391<br>0.0358<br>146	-0.04347<br>0.6166<br>135
**perception_inst_admit**<br>perception_inst_admit	-0.13351<br>0.1081<br>146	0.13819<br>0.1100<br>135

Lavie National survey: correlations with 95% CI

The CORR Procedure

status2=1

Pearson Correlation Statistics (Fisher's z Transformation)									
Variable	With Variable	N	Sample Correlation	Fisher's z	Bias Adjustment	Correlation Estimate	95% Confidence Limits		p Value for H0:Rho=0
**admit5**	**paternalism**	145	0.06196	0.06204	0.0002152	0.06175	-0.102289	0.222519	0.4597
**avgabscon5**	**paternalism**	134	0.12920	0.12992	0.0004857	0.12872	-0.041781	0.291935	0.1370
**admit5**	**worry_px**	145	0.20842	0.21152	0.0007237	0.20773	0.046286	0.358594	0.0117
**avgabscon5**	**worry_px**	134	-0.09703	-0.09734	-0.0003648	-0.09667	-0.261966	0.074131	0.2652
**admit5**	**use_laws**	146	-0.14287	-0.14386	-0.0004927	-0.14239	-0.297946	0.020534	0.0854
**avgabscon5**	**use_laws**	135	0.10895	0.10939	0.0004065	0.10855	-0.061533	0.272512	0.2088
**admit5**	**risk_comfort**	146	-0.11240	-0.11288	-0.0003876	-0.11202	-0.269560	0.051367	0.1771
**avgabscon5**	**risk_comfort**	135	0.37989	0.39993	0.00142	0.37868	0.224054	0.514703	<.0001
**admit5**	**ih_benefits**	146	0.07811	0.07827	0.0002693	0.07784	-0.085694	0.237286	0.3493
**avgabscon5**	**ih_benefits**	135	0.01101	0.01101	0.0000411	0.01097	-0.158282	0.179593	0.8993
**admit5**	**perception_self_admit**	146	-0.17391	-0.17569	-0.0005997	-0.17333	-0.326580	-0.011194	0.0356
**avgabscon5**	**perception_self_admit**	135	-0.04347	-0.04350	-0.0001622	-0.04331	-0.210727	0.126571	0.6172
**admit5**	**perception_inst_admit**	146	-0.13351	-0.13432	-0.0004604	-0.13306	-0.289258	0.030035	0.1082
**avgabscon5**	**perception_inst_admit**	135	0.13819	0.13908	0.0005156	0.13768	-0.032020	0.299668	0.1101

The CORR Procedure

**2 With Variables:**	yearspracticing percent_er
**1 Variables:**	avgabscon5

Simple Statistics							
**Variable**	**N**	**Mean**	**Std Dev**	**Sum**	**Minimum**	**Maximum**	**Label**
**yearspracticing**	96	2.58333	1.86754	248.00000	1.00000	9.00000	yearspracticing
**percent_er**	96	2.16667	1.63299	208.00000	1.00000	6.00000	percent_er
**avgabscon5**	223	68.03677	14.21183	15172	21.00000	100.00000	

Pearson Correlation Coefficients<br>Prob > |r| under H0: Rho=0<br>Number of Observations	
	**avgabscon5**
**yearspracticing**<br>yearspracticing	0.08791<br>0.4237<br>85
**percent_er**<br>percent_er	0.21775<br>0.0453<br>85

Pearson Correlation Statistics (Fisher's z Transformation)									
**Variable**	**With Variable**	**N**	**Sample Correlation**	**Fisher's z**	**Bias Adjustment**	**Correlation Estimate**	**95% Confidence Limits**		**p Value for H0:Rho=0**
**avgabscon5**	**yearspracticing**	85	0.08791	0.08814	0.0005233	0.08739	-0.128121	0.295019	0.4248
**avgabscon5**	**percent_er**	85	0.21775	0.22129	0.00130	0.21651	0.003553	0.410687	0.0451

Lavie National survey: correlations with 95% CI

The CORR Procedure

**3 With Variables:**	PGY pgy2 time_er
**1 Variables:**	avgabscon5

Simple Statistics							
**Variable**	**N**	**Mean**	**Std Dev**	**Sum**	**Minimum**	**Maximum**	**Label**
**PGY**	147	2.51701	1.20133	370.00000	1.00000	5.00000	PGY
**pgy2**	147	2.46259	1.10585	362.00000	1.00000	4.00000	
**time_er**	147	3.04082	0.78407	447.00000	1.00000	4.00000	time_er
**avgabscon5**	223	68.03677	14.21183	15172	21.00000	100.00000	

Pearson Correlation Coefficients<br>Prob > |r| under H0: Rho=0<br>Number of Observations	
	**avgabscon5**
**PGY**<br>PGY	0.32323<br>0.0001<br>136
**pgy2**	0.35205<br><.0001<br>136
**time_er**<br>time_er	0.15854<br>0.0653<br>136

Pearson Correlation Statistics (Fisher's z Transformation)									
**Variable**	**With Variable**	**N**	**Sample Correlation**	**Fisher's z**	**Bias Adjustment**	**Correlation Estimate**	**95% Confidence Limits**		**p Value for H0:Rho=0**
**avgabscon5**	**PGY**	136	0.32323	0.33525	0.00120	0.32216	0.162643	0.465258	0.0001
**avgabscon5**	**pgy2**	136	0.35205	0.36778	0.00130	0.35091	0.194037	0.490281	<.0001
**avgabscon5**	**time_er**	136	0.15854	0.15989	0.0005872	0.15797	-0.010650	0.317847	0.0652

```
******************************************************************************;
* this file produces results for the manuscript on physician characteristics
******************************************************************************;

**************************************************************************;
* FEB 27, 2026. FILE AMENDED TO ADDRESS R1 CONCERNS FROM PLOS MH REVIEWS ;
**************************************************************************;


PROC IMPORT
DATAFILE='location'; OUT=national
DBMS=SAV replace;
RUN;


data national2;
set national;

*recoding decision data so that 1=admit and 0=discharge;
if d_1=2 then rd_1=0;
else if d_1=1 then rd_1=1;
if d_2=2 then rd_2=0;
else if d_2=1 then rd_2=1;
if d_3=2 then rd_3=0;
else if d_3=1 then rd_3=1;
if d_4=2 then rd_4=0;
else if d_4=1 then rd_4=1;
if d_5=2 then rd_5=0;
else if d_5=1 then rd_5=1;
if d_6=2 then rd_6=0;
else if d_6=1 then rd_6=1;
if d_7=2 then rd_7=0;
else if d_7=1 then rd_7=1;
if d_8=2 then rd_8=0;
else if d_8=1 then rd_8=1;


missd=nmiss(of d_1 d_2 d_3 d_4 d_5 d_6 d_7 d_8);
missc=nmiss(of c_1 c_2 c_3 c_4 c_5 c_6 c_7 c_8);

missd5=nmiss(of d_3 d_4 d_5 d_6 d_8);
missc5=nmiss(of c_3 c_4 c_5 c_6 c_8);

*create a variable for proportion of admit decisions;
admit5=mean(of rd_3 rd_4 rd_5 rd_6 rd_8);
admit5B=SUM(of rd_3 rd_4 rd_5 rd_6 rd_8);

*remove 2 cases with missing data on the decision;
if UID=55 or UID=128 then delete;
*if missc5 gt 0;

*recode missing confidence data for anyone missing 1 or 2 of the 5 central vignettes;
if missc5 le 2 then do;
array recode5m c_3 c_4 c_5 c_6 c_8;
do over recode5m;
```

```
if recode5m=. then recode5m=50;
end;
end;

*use decision variables to weigh confidence scores;
*admit scores are positive;
*discharge scores are all multiplied by -1;
if rd_3=0 then rc_3=c_3*-1;
else if rd_3=1 then rc_3=c_3;

if rd_4=0 then rc_4=c_4*-1;
else if rd_4=1 then rc_4=c_4;

if rd_5=0 then rc_5=c_5*-1;
else if rd_5=1 then rc_5=c_5;

if rd_6=0 then rc_6=c_6*-1;
else if rd_6=1 then rc_6=c_6;

if rd_8=0 then rc_8=c_8*-1;
else if rd_8=1 then rc_8=c_8;


*0 is not a valid training region. Flags people who skipped question or demo survey;
if training_region_combined=0 then training_region_combined=.;

*attending versus resident (fellows currently not included);
if status=0 then attending=1;
else if status=1 then attending=0;

avgabscon5=mean(of c_3 c_4 c_5 c_6 c_8);
avgreccon5=mean(of rc_3 rc_4 rc_5 rc_6 rc_8);
if missc5 ge 3 then avgabscon5=.;
if missc5 ge 3 then avgreccon5=.;

run;

******************************************************************************;
******************************************************************************;
******************************************************************************;

**********************;
* CALCULATE VARIABLES;
**********************;

data national3;
set national2;

gender=catx('_', man, woman, QU, NB);
label gender= "man, woman, QU, NB";

* two versions of gender;
if man=1 then male=1;
else male=0;

if tm=1 or tw=1 or qu=1 or nb=1 or ogender=1 then malefemale=.;
```

```
else if N_gender=0 then malefemale=.;
else malefemale=male;

races=catx('_', White, Black, SAsian, EAsian, MENA, ORace, skip_Race);
label races= "White, Black, SAsian, EAsian, MENA, ORace, skip_race";

*Coding Non-Hispanic White versus other races, multiple races, and/or Latino;
if white=1 and N_race=1 and Latino ne 1 then minority=0;
else minority=1;
if N_Race=0 or skip_race=1 then minority=.;

*collapsing age into 3 categories, < 30, 30s, and 40+;
if focus1_age=1 or focus1_age=2 then agecat3=1;
else if focus1_age=3 then agecat3=2;
else if focus1_age ge 4 then agecat3=3;


*collapsing age into 2 categories, < 40 and 40+;
if focus1_age=1 or focus1_age=2 or focus1_age=3 then agecat2a=1;
else if focus1_age ge 4 then agecat2a=2;

*collapsing age into 2 categories, < 30 and 30+;
if focus1_age=1 or focus1_age=2 then agecat2b=1;
else if focus1_age ge 3 then agecat2b=2;

*creating 2nd version of status where fellows are combined with residents;
if status=2 then status2=1;
else status2=status;

*combining Fellows with PGY4 given small cells;
if pgy=5 then pgy2=4;
else pgy2=pgy;

if percent_er=1 then erexp=0;
else if percent_er ge 2 then erexp=1;

***************************************************************************;
* NOTE. Recode so that higher scores = MORE paternalism.
* Items 1-5 are the ones that load on the paternalism factor.
* Note that paternalism items were coded so that higher = LESS paternalism.
* Create a reverse-coded score so that higher scores = MORE paternalism;
***************************************************************************;
rpat1=6-pat1;
rpat2=6-pat2;
rpat3=6-pat3;
rpat4=6-pat4;
rpat5=6-pat5;


paternalism=mean(of rpat1 rpat2 rpat3 rpat4 rpat5);


*Practice settings;
*three variables for setting: outpx, inpx, unclear;
if amc_opx=1 or comm_opx=1 or sud_opx=1 or sud_ipx=1 or PHP=1 or private=1 then outpx=1;
else outpx=0;
```

```
if amc_ipx=1 or eps=1 or comm_ipx=1 or state_H=1 or CL=1 then inpx=1;
else inpx=0;
*make a 2nd version of inpx that excludes PES, given the opposite pattern of results;
if amc_ipx=1 or comm_ipx=1 or state_H=1 or CL=1 then inpx2=1;
else inpx2=0;
if VA_H=1 or County_H=1 or Correctional=1 then ukpx=1;
else ukpx=0;

settings=catx('_', ukpx, outpx, inpx, eps, inpx2 );
label settings= "other_outpx_inpxORpes_pes_inpxNOpes";
settings2=catx('_', outpx, eps, inpx2 );
label settings2= "outpx__pes_inpxNOpes";
esettings=catx('_', eps, percent_er, pes );
label esettings= "eps__percent_er_pes";

run;

**************************************************************************************;
**************************************************************************************;
**************************************************************************************;

 * OUTPUT FILES FOR RESULTS

 **************************************************************************************;
 **************************************************************************************;
 **************************************************************************************;


 ******************************************;
 * PLoS MH REVISION ANALYSES FROM FEB 2026
 ******************************************;


 ODS PDF FILE='location'; title1 'Lavie National survey: correlations with 95% CI';
 data nat3;
 set national3;
 proc corr fisher;
 var admit5 avgabscon5;
 with paternalism worry_px use_laws risk_comfort ih_benefits perception_self_admit
 perception_inst_admit run;
 proc sort;
 by status2;
```

```
proc corr fisher;
var admit5 avgabscon5;
with paternalism worry_px use_laws risk_comfort ih_benefits perception_self_admit
perception_inst_admit; by status2;
run;

*confidence. attendings only. Results reported in text;
proc corr fisher;
var avgabscon5;
with yearspracticing percent_er;
run;
*confidence. trainees only. Results reported in text;
proc corr fisher;
var avgabscon5;
with pgy pgy2 time_er;
run;

ODS PDF CLOSE;
run;
title1;
title2;
title3;

*EASIER TO CALCULATE EFFECT SIZES AND 95% ciS IN SPSS. EXPORTING DATA;
proc export data=national3
outfile='location_filename' dbms=spss replace;
run;

***************************************************************;
***************************************************************;
***************************************************************;
***************************************************************;
* END REVISION ANALYSES 2.27.2026;


**************************************************************;
* ANALYSES FROM OCTOBER 2024 REPORTED IN ORIGINAL SUBMISSION
**************************************************************;



*ODS PDF FILE='location_filename'; data nat4;
set national3;

missdemos=nmiss(of site_region agecat3 malefemale minority status);
run;

title2 'Descriptive Information';
title3 'Missing data in main variables';
proc freq;
table missc5 missdemos;
run;

title3 'Demographic variable frequencies';
```

```
proc freq;
table gender malefemale white black SAsian EAsian MENA Orace skip_race N_race Latino minority
agecat3 status status2 site_region/missing;
run;

proc freq;
table malefemale*gender/missing;
run;
proc freq;
table races*minority/missing;
run;
proc freq;
table focus1_age*agecat3/missing;
run;

title3 'Ns and Means of main variables';
proc means;
var admit5 avgabscon5 siteno malefemale agecat3 minority
paternalism worry_px use_laws risk_comfort ih_benefits perception_self_admit
perception_inst_admit; run;

title3 'distribution of admit decisions';
proc freq;
table admit5;
run;

title3 'correlations between admit rates, confidence in admit, and absolute level of confidence ';
proc corr;
var admit5 avgreccon5 avgabscon5 ;
run;

data attendings;
set nat4;
if status=0;
title2 'descriptive data for attendings only';
title3 ;
proc freq;
table yearspracticing training_pes percent_er er_exp;
run;


title3 'settings';
proc freq;
table eps
outpx private amc_opx comm_opx php sud_opx*sud_ipx
inpx2 amc_ipx comm_ipx state_h cl
ukpx correctional VA_H County_H/missing;
run;
proc freq;
table N_settings settings2 inpx2 outpx eps;
run;
proc freq;
table esettings;
run;
proc freq;
```

```
table pes*er_exp;
run;
proc corr;
var inpx2 outpx pes er_exp;
run;


data trainees;
set nat4;
if status2=1;
title2 'descriptive data for trainees only';
title3 ;
proc freq;
table pgy;
run;

*ODS PDF CLOSE;
run;

*****************************************************************;
*****************************************************************;
*****************************************************************;

ODS PDF FILE='location_filename'; data nat4b;
set nat4;

title2 'Significance testing for admit likelihood';
title3;
proc ttest;
class malefemale;
var admit5;
run;
proc ttest;
class minority;
var admit5;
run;
proc mixed;
class agecat3;
model admit5=agecat3;
lsmeans agecat3;
run;
/*
proc ttest;
class agecat2a;
var admit5;
run;
proc ttest;
class agecat2b;
var admit5;
run;
*/;
proc ttest;
class status2;
var admit5;
run;
```

```
proc mixed;
class site_region;
model admit5=site_region;
lsmeans site_region /pdiff;
run;

data attendings;
set nat4;
if status=0;
title3 'predictors for attendings only';

proc ttest;
class er_exp;
var admit5;
run;

proc ttest;
class eps;
var admit5;
run;

proc ttest;
class inpx2;
var admit5;
run;

proc ttest;
class outpx;
var admit5;
run;

proc ttest;
class pes;
var admit5;
run;

proc ttest;
class training_pes;
var admit5;
run;

proc corr;
var admit5;
with yearspracticing percent_er;
run;

*TRAINEES ONLY.;
data PGYfellow;
set nat4;
if status2=1;
title3 'predictors for trainees only';

if pgy=5 then pgy2=4;
else pgy2=pgy;
```

```
proc corr;
var admit5;
with pgy pgy2 time_er;
run;


* full sample (attendings + trainess) personality-related analyses;
data all;
set nat4;
title3 'full sample (attendings + trainess) personality-related analyses';
proc corr;
var admit5 ;
with paternalism worry_px use_laws risk_comfort ih_benefits perception_self_admit
perception_inst_admit; run;
ODS PDF CLOSE;
run;




****************************************************************;
****************************************************************;
****************************************************************;

ODS PDF FILE='location_filename; data nat4b;
set nat4;

title2 'Significance testing for admit likelihood';
title3;
proc ttest;
class malefemale;
var avgabscon5;
run;
proc ttest;
class minority;
var avgabscon5;
run;
proc mixed;
class agecat3;
model avgabscon5=agecat3;
lsmeans agecat3;
run;
/*
proc ttest;
class agecat2a;
var avgabscon5;
run;
proc ttest;
class agecat2b;
var avgabscon5;
run;
*/;
proc ttest;
class status2;
var avgabscon5;
```

```
run;
proc mixed;
class site_region;
model avgabscon5=site_region;
lsmeans site_region /pdiff;
run;
data attendings;
set nat4;
if status=0;
title3 'predictors for attendings only';

proc ttest;
class er_exp;
var avgabscon5;
run;

proc ttest;
class eps;
var avgabscon5;
run;

proc ttest;
class inpx2;
var avgabscon5;
run;

proc ttest;
class outpx;
var avgabscon5;
run;

proc ttest;
class pes;
var avgabscon5;
run;

proc ttest;
class training_pes;
var avgabscon5;
run;

proc corr;
var avgabscon5;
with yearspracticing percent_er;
run;

*TRAINEES ONLY.;
data PGYfellow;
set nat4;
if status2=1;
title3 'predictors for trainees only';

if pgy=5 then pgy2=4;
else pgy2=pgy;
```

```
proc corr;
var avgabscon5;
with pgy pgy2 time_er;
run;


* full sample (attendings + trainess) personality-related analyses;
data all;
set nat4;
title3 'full sample (attendings + trainess) personality-related analyses';
proc corr;
var avgabscon5 ;
with paternalism worry_px use_laws risk_comfort ih_benefits perception_self_admit
perception_inst_admit; run;
ODS PDF CLOSE;
run;

proc sort;
by status2;
proc ttest;
class malefemale;
var avgabscon5;
by status2;
run;
proc ttest;
class minority;
var avgabscon5;
by status2;
run;

ODS PDF FILE='location_filename';
proc corr;
var admit5 avgabscon5;
with paternalism worry_px use_laws risk_comfort ih_benefits perception_self_admit
perception_inst_admit; run;
proc sort;
by status2;
proc corr;
var admit5 avgabscon5;
with paternalism worry_px use_laws risk_comfort ih_benefits perception_self_admit
perception_inst_admit; by status2;
run;
ODS PDF CLOSE;
run;
```
