# Supplementary material for: Identifying psychiatrist characteristics associated with likelihood of recommending involuntary hospitalization for patients using a novel tool to assess decision-making
Source: PLOS Ment Health. 2026 Apr 20;3(4):e0000598. doi: 10.1371/journal.pmen.0000598 (PMC13095014; doi:10.1371/journal.pmen.0000598)
Supplement: Attachment — Submitted filename: Response_to_Reviewers.pdf [file pmen.0000598.s003.pdf]

RESPONSE TO REVIEWERS

Thank you for your feedback on our original submission. We are pleased that both reviewers found the study to be worthy of publication and that Reviewer 2 had only minor editorial suggestions. We have carefully considered each suggestion from each reviewer. In the following text, we have numbered the original comments and added our response below each comment. On occasion we have broken down longer comments into smaller points. Relevant sections of text added to the revised manuscript have been reproduced in italics and/or are referenced by the line number corresponding to the pdf file that contains <u>the track changes version</u> of the revised manuscript. Please note the revised manuscript does not have line numbers between 395 and 460. We are unsure what formatting caused this but in the below response letter we refer to the line numbers as shown in the marked version of the manuscript.

Journal Requirements:

1. Your current Financial Disclosure states, "This work was supported by an internal institutional Dean's Fund award to the first author.". However, your funding information on the submission form indicates that you did not receive funding. Please indicate by return email the full and correct funding information for your study and confirm the order in which funding contributions should appear. Please be sure to indicate whether the funders played any role in the study design, data collection and analysis, decision to publish, or preparation of the manuscript.

The funding information has been removed from the Acknowledgments section of the revised manuscript file. We have entered the information in the Financial Disclosures section with the additional text required on the role of the funder. We have emailed the journal office directly to ask them to edit the Funding Information section, as we are unable as authors to make changes after the initial submission.

2. Please provide separate figure files in .tif or .eps format.

Better quality figures in .tif format have been uploaded. Image files were run through the journal PACE tool to ensure adequate clarity for publication.

3. Please insert an Ethics Statement at the beginning of your Methods section, under a subheading 'Ethics Statement'. It must include:

> **1) The name(s) of the Institutional Review Board(s) or Ethics Committee(s)**
> **2) The approval number(s), or a statement that approval was granted by the named board(s)**
> **3) (for human participants/donors) - A statement that formal consent was obtained (must state whether verbal/written) OR the reason consent was not obtained (e.g. anonymity).**

An Ethics Statement with the required information has been added to the revised manuscript (L149)

4. We do not publish any copyright or trademark symbols that usually accompany proprietary names, eg (R), (C), or TM (e.g. next to drug or reagent names). Please remove all instances of trademark/copyright symbols throughout the text, including © on page 8.

All trademark symbols have been removed.

5. If the reviewer comments include a recommendation to cite specific previously published works, please review and evaluate these publications to determine whether they are relevant and should be cited. There is no requirement to cite these works unless the editor has indicated otherwise.

N/A

REVIEWER 1
Reviewer Recommendation Term: Major Revision

1. This is an important research topic and the study was well executed within available resource constraints. The authors do not step outside the limitations of cross-sectional online surveys. The writing is mostly clear, but the manuscript needs further work to do this justice before publication in my view.

Thank you for this positive feedback.

2. The research questions are not very precise and rather exploratory: "to better understand the role of physician characteristics on psychiatrists' individual differences in the IH decisions using the PIHD instrument in a national sample". Rather than a clear hypothesis or set of questions, the authors say "We expect there will be differences" across effect modifiers / subgroups (region, practice setting).

We agree that the study is exploratory and now state this explicitly in both the Introduction (L138) and Discussion (L465-466). There is a lack of prior research focused on how physician individual characteristics relate to medical decision-making regarding involuntary hospitalization. Instead, most previous studies have focused either on how patient characteristics impact IH, or on how IH rates vary across regions. Thus, the present study makes a novel contribution to existing literature by exploring whether IH decision-making and confidence varies across physician characteristics. We have added new text justifying this approach in the Introduction:

> *L132. By conducting analyses of cross-sectional study that includes data on a wide range of physician characteristics, we hope to gain initial insights on patterns that can be used to generate hypotheses about the factors that influence individual differences in physician IH decision-making. A better understanding of individual differences could lead to greater standardization of care across practitioners, which could in turn decrease bias and increase the quality of care for all patients."*

However, in both the original and the revised manuscript, we included details on the handful of studies that have examined physician characteristics and note that there is some limited evidence that demographics, clinician experience and training, and attitudes and beliefs have been associated with IH. These studies, presented on L94-109, justify our choice of measures. Moreover, we did include specific hypotheses at the end of the final paragraph of the Introduction (L138-144) so do not agree with the statement that there are no clear hypotheses or questions.

3. It is better in my opinion to focus on one or few risk factors / exposures / independent variables of primary interest and then treat other variables as covariates/confounders - subgroups as effect modifiers / interaction terms only if theoretically justified and sample size is large enough.

While we appreciate this reviewer's comments, as mentioned above, there is not enough existing evidence to determine which individual factors would be "main" predictors/risk factors rather than "covariates/confounders." Our goal was to capture a wide range of physician factors that have the potential to affect IH decision-making, including demographic characteristics, clinical and training experiences, and physician beliefs and attitudes.

4. It is difficult to recruit using online surveys and achieve large sample sizes (I have attempted this myself) so I empathise with the sample size issue, but I doubt there is enough statistical power for subgroup analyses here (unless you can convince me with a formal statistical power calculation).

While we appreciate the authors' concern, we do not believe that post-hoc power analyses are appropriate, as they are completely determined by the p-value. High p-values (i.e., non-significance) will always have low power. Low p-values will always have high power. Thus, nothing is learned from post hoc power calculations and these calculations can sometimes send misleading messages about the importance of non-significant effects (Dziak et al., 2021). Thus, the biostatistics expert on the team strongly discourages the use of post-hoc power calculations.

Dziak JJ, Dierker LC, Abar B. The Interpretation of Statistical Power after the Data have been Gathered. *Curr Psychol. 2020 Jun;39(3)*:870-877. doi: 10.1007/s12144-018-0018-1. Epub 2018 Oct 2. PMID: 32523323; PMCID: PMC7286546.

Moreover, we dispute the fact that the small cell sizes of our subgroups limited our ability to find statistically significant effects, as one of the significant differences in likelihood of admittance was found among those attendings who did or did not work within a Psychiatric Emergency Setting (PES). As only attending physicians who reported working in the ED were asked the follow-up question about the PES, this comparison was based on cell sizes of 24 vs 21 (Fig 1) yet the difference between groups was statistically significant at $p < .05$. Similarly, we find significant correlations between physician attitudes with PIHD outcomes among our subgroup of attendings, but not trainees, despite the fact that the latter group has twice the sample size of the former.

5. Going forward I recommend to pre-register very specific research questions (and any subgroups proposed) before analysis, on Open Science Framework (OSF) or similar. This is not a requirement for publication in PLOS Mental Health, but will improve precision and help convince peers that there were more precise questions beyond general understanding, expecting differences of some kind, and permitted a wide range of predictors to be "significant" (see point below - avoid p value thresholds).

We agree that future studies on this topic would benefit from pre-registration of specific hypotheses.

6. Many reviewers and readers will use critical appraisal checklists to review manuscripts (https://casp-uk.net/casp-checklists/CASP-checklist-cross-sectional-study-2024.pdf), and when reading this version, I nearly ticked "Can't tell" for the first question "Did the study address a clearly focused issue?". At which point, many would stop or have been trained to not continue reading any paper unless the answer is "yes" to this first question. I continued because I think the research has been conducted well and the aims/objectives are clear - they are just not clear enough in the writing yet (particularly at the end of the introduction). The authors understand what they have done, and I think I understand, but had to re-read several parts of the manuscript in order to reach or check my understanding, which is not ideal - it should be rewritten so that it cannot be misunderstood and readers don't have to check back to the introduction to remind themselves what the specific research questions were.

CASP 7. What is the main result - I can't tell yet, but this is not a flaw in the analysis (it just needs further work on the writing)

CASP 9. Is there a clear statement of findings - I can't tell yet.

Other questions are "yes" in my view - so this can all be addressed in a revision. I don't want to sound discouraging, there is lots of good work here, and it will be suitable for publication in my view when rewritten.

We appreciate this reviewer's use of the CASP checklist to aid in the review and are pleased that our manuscript met most of these rigorous criteria.

7. The take-home message is not clear enough yet. I struggled to get a sense of what this would add to our understanding - there are lots of interesting insights, but the writing doesn't tell the story currently. This is good work, but you need to hold our hands and connect the introduction, methods, results, conclusions together with a bolder narrative arc. This does matter in scientific writing, and will strengthen any revision. What is the message in 12 words?

The take-home message is that there are a wide range of physician demographic, training, clinical, and psychological characteristics that are associated with IH decision-making. As noted in newly added text in the Discussion (L468): *"Results from this study are important because they signal that there is significant variation in practice across psychiatrists when faced with the same clinical scenario."*

We have added also new text (highlighted) on the importance of the problem to the opening paragraphs of the introduction:

> *L37. Psychiatric involuntary hospitalization (IH) is utilized to ensure that individuals in imminent danger of harming themselves or others receive the required level of care. Nevertheless, the decision to hospitalize is often complex, involving a balance of risks, patient autonomy, and beneficence. Currently, there are no widely accepted, clinically grounded guidelines to standardize the decision-making process. Some psychiatrists are more conservative, valuing patient safety over autonomy, whereas other psychiatrists prioritize individual patient rights. Individual differences in how patients are treated can raise issues of quality of care. In psychiatry, this is particularly important for decisions regarding IH, given the ethical issues of removing, at least temporarily, patient autonomy in decision-making.*
>
> *To date, most studies of individual differences in IH have focused on how patient characteristics, such as demographics, diagnosis, and symptom severity are associated with IH [1-5]. However, fewer studies have focused on how contextual factors and physician characteristics relate to IH decision-making [6]. Indeed, at present, there has been no systematic evaluation of whether psychiatrists across the United States (U.S.) have similar thresholds for IH or to identify the factors that influence their decisions.*

Thus, our paper is focused on how physician characteristics may be associated with individual differences in IH decision-making, which is also stated clearly towards the end of the Introduction, which also includes new text (highlighted):

> *L130. Thus, the goal of the current study is to better understand the role of physician characteristics on psychiatrists' individual differences in the IH decisions using the PIHD instrument in a national sample. By conducting analyses of cross-sectional study that includes data on a wide range of physician characteristics, we hope to gain initial insights on patterns that can be used to generate hypotheses about the factors that influence individual differences in physician IH decision-making. A better understanding*

of individual differences could lead to greater standardization of care across practitioners, which could in turn decrease bias and increase the quality of care for all patients.

8. The data is not yet fully available. It is not clear where readers can find the data.

An Excel file with the data has been uploaded as a Supporting Information file. The file contains both the raw item-level data from the survey as well as calculated variables used in the final statistical analyses. A separate worksheet provides a codebook for the variables.

9. L41 For readers outside US, can you provide some detail on the extent to which state vs federal laws (between and within) vary? Variation in psychiatrists' decisions is the focus, but presumably there is variation across states to consider. L50 onwards has a good summary of international variation (this could be a review paper in its own right!) but for this paper, the between and within state variation is more relevant I think.

We have removed the reference to US federal and state laws from this sentence on L41, as going into the details of how these differ is beyond the scope of the Introductory paragraph. However, in both the original and revised manuscript, we have provided some examples of differences in laws across states in the Discussion when interpreting the pattern of results for regional differences.

> *L475. "Local laws and structural barriers may partially account for these regional differences by raising the threshold for when someone can be admitted involuntarily. For example, in California, a court order is required for IH, whereas in New York, it is not [24]. Additionally, although most states require the criteria of danger to self and others, Georgia only requires the criterion of being mentally ill to be admitted involuntarily [24]. In the U.S., 22 states have adopted the 72-hour hold, whereas other states limit holds to 24/48 hours [24]. Previous research indicates 72-hour holds lead to decreased admission rates, perhaps by allowing more time for patient stabilization [25].*

10. In case interesting/relevant, in the United Kingdom, sectioning (forced hospitalization) requires two psychiatrists, and an AMHP (social worker, nurse, occupational therapist or psychologist). The AMHP could prevent hospitalisation against the recommendation of the two psychiatrists.
https://www.legislation.gov.uk/ukpga/2007/12/notes/division/6/1/2?view=plain

Thank you for bringing this to our attention. In researching the UK Mental Health Act and its 2007 amendment, we further came across a peer-reviewed paper that summarized differences between the UK and Italy. We have now added this citation to the paper:

> *L65. "For example, in the United Kingdom, IH requires the approval of two medical practitioners, one of whom must be a psychiatrist, as well as approval from a non-medical professional who has been specially trained as an Approved Mental Health Professional, while in Italy, the patients must have an initial assessment by a medical practitioner and by a second physician belonging to the Local Health Unit, and the Mayor of the patient's municipality must formally authorize the IH [14]."*

11. L67 This is confusing because four locations are compared, two with relative and two with absolute measures. If possible, report the absolute for all four (or relative for all four). It reads awkwardly if these difference lenses are mixed.

While we appreciate this feedback, the statistics for the 4 different states were drawn from 2 different citations that did not calculate IH rates using the same metrics—the Lee & Cohen (2021) article used a population-based rate of number of IH per 100,000 people, while the SAMHSA data was based on a subset of people with serious mental illness, and were expressed as rates per 1,000 persons with serious mental illness. Thus, there was no way to compare the rates across all 4 states directly.

Unfortunately, the link to the SAMHSA data is no longer active at the time of this revision, so we have deleted the information and citation from the paper and now focus solely on the peer-reviewed study citing a 33-fold difference reported between the lowest IH-rate state (Connecticut) and the highest IH-rate state (Florida). We have added the absolute rates per 100,000 people for the two states and clarified that they represent the lowest and highest rates reported. The exact text now reads:

> *L76. "Within the U.S., IH rates vary widely by state and region. For example, one analysis of IH rates (per 100,000 persons) across 25 US states between 2011-2018 reported that IH rates ranged from a low of 0.00029 (Connecticut) to a high of .00966 (Florida), a 33-fold difference [16]."*

12. L68 This is a crucial point - without crisis support, severity of psychosis might be much higher at the point hospitalization is required. Consider expanding this. When under-resourced, are psychiatrists more likely to recommend hospitalization because severity of episode at the point of presentation is higher than it might be if crisis and other support had been available earlier? Is this a confounding factor - the decisions might be quite different if presenting with less severe symptoms owing to other sources of support.

We agree that access to other crisis support interventions is likely to play a key role in decision-making. Thus, we have expanded on this point in the Introduction with new text and a new citation:

> *L79. "For example, analysis of more than 2600 patient evaluations throughout the state of Virginia revealed that fewer community-based alternatives to hospitalization, such as temporary housing or short-term crisis stabilization, were associated with IH decisions [17]."*

13. …can you strengthen the argument that the vignettes fully capture scenarios where things have gotten so serious that hospital admission is almost certainly needed, vs. someone has found earlier, alternative support, and might not need it?

Please note that the development and validation of the PIHD instrument has been published previously and the reference is cited in the current manuscript (L182). The vignettes are not intended to be restricted to situations where things have gotten so serious that hospital admission is almost certainly needed. Indeed, the PIHD intentionally focuses on ambiguous clinical situations.

As reported in the original and revised manuscripts, the PIHD instrument includes two anchor vignettes—one in which there is a clear decision to admit and one where there is a clear decision to discharge. In this sample, as well as in our previously published sample, over 99% of respondents

correctly answered both anchor questions (reported in Results on L303-304), indicating they accurately captured the intended clinical severity. Responses to these vignettes are not analyzed, given the lack of individual variability, but are provided as validity checks for the measure.

In contrast, the 5 vignettes analyzed were designed to be clinically complex, to maximize the individual differences in physician response. As reported in the Results of the current paper (L305-307), "…*the average likelihood of admittance score for the five complex vignettes was 0.48 (SD = 0.25) and individual responses ranged from no admit decisions (0) to all admit decisions (1)*", indicating that the vignettes did indeed maximize physician response.

14. L81 Again, I think worth mentioning that some countries require two psychiatrists to decide, which may lower concern (particularly if one is more experienced than the other).

We added the suggested information that the number of physicians required for IH varies in our earlier paragraph on structural differences across countries, including discussion of differences in legal requirements (see response to Comment #10). As the paragraph the reviewer is referring to here is about prior research on individual-level characteristics, not macro-level factors, we have opted not to repeat this fact here.

15. L110 It is not an essential requirement, but consider pre-registering your research question on Open Science Framework (OSF) or similar with hypotheses, subgroups etc.

As noted under Comment #5, we agree that future studies on this topic would benefit from pre-registration of specific hypotheses. Pre-registration protects transparency, validity, and trust in scientific results.

Regarding the suggestion that we also <u>register hypotheses for the current study</u>, we have opted not to do so. The biostatistics expert on the team cautions against registering hypotheses after data have been collected and analyzed, which is often referred to as "HARKing" (Hypothesizing After Results are Known; see Kerr, 1998). Registering hypotheses after analysis introduces confusion between exploratory and confirmatory analyses, which misrepresents the research process, affecting research reproducibility and trust. HARKing can further damage scientific integrity, increase bias, and misrepresent findings as more certain than is warranted. Thus, we have not registered hypotheses for the current study.

Kerr, N. L. (1998). HARKing: Hypothesizing after the results are known. *Personality and social psychology review, 2(3)*, 196-217.

16. L115 Report N and % psychiatrists vs trainees.

For this paper we think it is more appropriate to present the sample descriptive information in the Results section. First, we want to be transparent about our response rate, so in the results we report our estimated target population (N=798) and the number of surveys received with useable data (N=246). Second, the distinction between attending psychiatrists versus trainee is one of our main study predictors, so we think presenting the specific Ns for attending psychiatrists versus trainees (and distinguishing between residents and fellows) is best kept in Table 1, which also contains the Ns for all the other subgroups.

17. L117 Report ethical approval number/reference

Details have been added to a new section called *Ethics Statement* (L149).

18. L123 Compensated how (was it identifiable data)? How much for how long?

We have added (L166): "*The survey took approximately 10 minutes to complete. Participants were compensated for their time with a $25 Amazon giftcard that was sent electronically*."

19. L148-157 Did you consider possible differences between men/women?

We believe the reviewer missed this information in the original manuscript.

In the Methods under the *Participant demographic characteristics and clinical experience* section, we stated (L199): "*All subjects were given multiple choice questions assessing age,* ***gender****, race, ethnicity, and attending versus trainee status*."

The mean outcome scores for males and females were shown in Figs 1 and 2 and results of analyses were summarized in the Results. We do note that we could have been clearer in stating that the only subgroup difference for the confidence in decision-making outcome was for the regional differences, so have added the following sentence (L349-350): "*None of the other differences across the subgroups shown in Fig 2 were statistically significant*."

20. L173 What is meant by race vs. ethnicity? Why not "ethnic group"

The separation of race and ethnicity is a common practice in US research. We have expanded on these distinctions in new text added to the Methods (L200):

> *"In accordance with guidelines used to collect data on the US population during the decennial census, separate questions were asked regarding participant racial and ethnic group. Respondents were asked to choose from a list of eight racial categories (White, Black, Southeast Asian, East Asian, Native American/Alaskan Native, Native Hawaiian/Other Pacific Islander, Middle Eastern/North African, and other racial group not listed). Multiple responses were permitted. Respondents then answered a yes or no question on whether they of Hispanic or Latino origin (ethnicity)."*

21. L182 Typo "for a response rate" - presumably "a response rate"

Thank you for noticing this typo. The word "for" has been deleted (L266).

22. L190 Now reaching Table 1, gender differences are included. But why not mentioned above with other characteristics? Small cell counts should be treated with caution, because these cannot be meaningfully analysed as categories (n = 3). Be cautious for statistical disclosure concerns prior to publication (e.g. in the UK, Office of National Statistics say no cells <10 people should be published even if risk of identification is low). I would report that small groups were included/eligible/represented, but excluded for practical/statistical reasons.

Please see our response to Comment #19 regarding the earlier mention of gender differences.

Regarding the small cell sizes, Table 1 is a descriptive table which presents the data from all N=246 respondents. Thus, we have included all responses, including those with low-frequency, and also noted the number of subjects with missing data for each categorical variable.

We note, however, that this is not how the data were analyzed. As was described in the original and revised manuscript, our *Statistical Analysis* section (L242) details how the data in the categorical subgroups were combined for the analyses. For example, although Table 1 shows that the age group question had 7 different response options (plus the No response category), for analytic purposes these groups were collapsed into three groups as described on L255-257.

In addition, the data presented in Figs 1 and 2 list the subgroups that were compared, along with the cell sizes (e.g., males versus females; thus, omitting the N=3 respondents who selected “Other Gender” and the N=3 respondents who did not provide a response).

However, to make this explicit, we have added the following information to the *Statistical Analysis* section:

> L247. “*Missing responses were omitted from statistical analyses. Low-frequency responses were either omitted (i.e., “other” gender) or combined with other categories to minimize small cell sizes.*”

23. For readers outside the US, explain Current Status (Attending, Resident, Fellow) and what is "trainee" vs. "psychiatrist"? Is there a direct mapping onto year in training, if they were cross-tabulated? Surely they are very highly correlated? Do you need both?

We have added details on the differences between attending, resident, and fellows:

> *L208. “In the US, physicians who have completed medical school do not become practicing psychiatrists until they have completed at least four years of training from an accredited institution and have obtained a state medical license to practice psychiatry, which requires passing all licensing exams and background checks required by the state they will be practicing in. Adult psychiatrists require four years of specialized training after medical school, and child psychiatrists must have at least three years of adult psychiatry training and two additional years of child psychiatry training. After completing adult or child psychiatry training, physicians can also opt to complete one- or two-year fellowships in psychiatric subspecialties. Physicians in training for adult psychiatry are referred to as residents. Physicians who are completing the two-year child psychiatry training or other specialized psychiatry training are referred to as fellows. Physicians who have completed their training and are employed at academic medical centers are referred to as attending psychiatrists.”*

The year in training question was asked only among trainees, as stated on L221. The range of years was shown in Table 1, although in this revision we have removed the preface PGY (which stands for Post [medical school] Graduate Year). The years in practice question was asked only among attending psychiatrists, as stated on L222-223, with the different response options shown in Table 1.

While both variables are assessing level of experience, they do not correlate with the trainee versus attending status variable, as they reflect the amount of experience relative to their peers. Note further that while the comparison of outcomes between trainees and attending is based on the full sample (i.e.,

comparing responses from the 96 attendings versus 147 trainees), correlations between years of training and outcomes are calculated only among the subset of 147 trainees, while correlations of years of experience and outcomes are calculated only among the 96 attending physicians.

24. L167 Is "Statistical analysis" better here as a heading?

Changed as suggested (L242).

25. L198 The analytic sample has changed to N = 246 (previously 248). It is clear why, but for simplicity I think it is better to define analytic sample as those with available data and use the same N throughout, if missing data proportion is small and completely at random. If only 2 people are lost, this is immaterial. Report as excluded from the off.

We have made the change as suggested. Please see text on L266.

26. L271 I recommend to avoid p values and use 95% confidence intervals (here, and throughout). p values are sensitive to sample size. CIs convey the same information, and additionally the precision of the estimates. The effect sizes (R, beta etc.) convey the effect size.

We have removed the p-values from the correlations shown in Table 2 and added 95% CIs around the correlation coefficients, as suggested. We note that the data presented in Figures 1 and 2 show the precision of estimates for each subgroup, as indicated by the conventional standard errors (also see response to Comment #29, below). While we have kept the p-values for the comparisons across subgroups described in the Results text (lines 310-330 and 344-355), we have added effect sizes (Coen's *d* for comparisons of two groups; omega-squared for F-tests) and their 95% CIs for all significant effects.

27. L277 "This study explored factors" is not a good way to start a discussion section of a scientific paper. Start with a bold reminder of the message.

This was a great suggestion. The opening paragraph of the Discussion now reads:

> *L413. "This study examined how individual physician characteristics and attitudes affect IH decision-making in a sample of U.S. psychiatrists. A novel feature of the study is that it used a standardized, vignette-based instrument to assess both likelihood of IH and confidence in decision-making. This study adds to a limited body of work understanding individual differences in IH decision-making, most of which has been done outside the U.S. Although our exploratory approach requires replication in other samples, we found that several factors were associated with IH decision-making, but patterns differed for individual differences in likelihood of admittance versus confidence in decision. Results from this study are important because they signal that there is significant variation in practice across psychiatrists when faced with the same clinical scenario."*

28. L346 There is unlikely to be a conflict here, but it is recommended to include a statement that the funder (even if at the Institution) had no role in the research question / study conception , analysis, decision to publish etc.

As directed by the Editorial Office, we have removed statements regarding funding from the Acknowledgments section and placed them in a separate Financial Disclosures section during submission of the revision. Thus, there is no longer an Acknowledgements section in the revised manuscript. As directed, in the Financial Disclosures section we have included the statements that the funder played no role in the project.

29. Figure 1 and 2 - these are not referenced in the text. What is their purpose, when we have tables already? Are the bars standard errors or confidence intervals?

This statement is not correct. The two figures were referenced in the text of the original manuscript: Figure 1 was first referenced on L207 and Figure 2 was first referenced on L234. In the revised manuscript, these references now occur on L310 and L344, respectively.

Per journal submission instructions, the figures were uploaded as separate .tif files, but the figure titles/headings and notes were included in the text of the manuscript following the paragraph in which they are first referred to (currently L332-337 show the title/heading for Figure 1; L357-361 show the title/heading for Figure 2). Note the figure headings in the manuscript text contain notes stating that the bars reflect the means for each subgroup with error bars denoting standard errors for each subgroup.

We apologize that the two figures were hard to read in the original submission and have uploaded new versions of the image files for this revision that we have run through the journal's PACE tool to confirm that they are of acceptable quality for publication.

30. I don't think the figure is needed, the table can include all the information here.

We do not agree with this statement.
While the Results text presents the results of the statistical analyses, i.e., the statistical tests and p-values, the 2 figures are critical for presenting the means and standard errors for each of the different subcategories in an efficient and engaging manner. Presenting means across subgroups in graph format makes it easier to see patterns, to judge the practical significance of mean differences among subgroups, and to compare patterns of results for the likelihood of admittance outcome (Fig 1) and the confidence in decision outcome (Fig 2).

To clarify further, the 2 tables and 2 figures that have been included in the original and the revised manuscript each present a unique set of results:

Table 1. Description of Sample Characteristics for the full N=246, not linked to either PIHD outcome. These data show the cell sizes for the full set of responses, including responses (e.g., missing data) that were not included in the statistical analyses,
Figure 1. Summary plots showing average likelihood of admittance scores across various categorical subgroups. Note the figure also shows how categories were combined and reports the exact Ns of each cell that was included in the statistical analysis.
Figure 2. Summary plots showing average confidence in decision scores across various categorical subgroups. Note the figure also shows how categories were combined and reports the exact Ns of each cell that was included in the statistical analysis.
Table 2. Correlations of the average likelihood of admittance scores AND average confidence in decision scores WITH continuous measures of physician attitudes and beliefs.

REVIEWER 2
Reviewer Recommendation Term: accept with comments

1. L50-54: valid point made about variations in legal policies and regulations across countries, with a relevant reference given

We appreciate the positive feedback.

2. L54: "The study authors also found that dangers to oneself was not a prerequisite…."
Suggested edit: "In this study, the authors also found that… / the authors in the study also found that…"

Changed as suggested (L57).

3. L59: "There are also national differences in the number and type of experts required for certification."
Comment: Please provide additional information into what "certification" entails

Cerification was referring to the legal process that leads to involuntary hospitalization (IH). Because this does vary across countries and states, we have replaced the term certification with IH. (L65)

4. L68-70: Accessibility is an important point for this paper, can benefit from additional discussion

We have added a specific example of how accessibility impacts IH decision-making and included a new citation for this statement:

> *L79. "For example, analysis of more than 2600 patient evaluations throughout the state of Virginia revealed that fewer community-based alternatives to hospitalization, such as temporary housing or short-term crisis stabilization, were associated with IH decisions [17]."*

5. L159: "...was used to assess physician's views on the…."
Suggested edit: "physicians' views on the…"

Changed as suggested (L233).

6. L311-312: "If someone is worried about patient safety, admitting them to care would reduce that concern."
Suggested edit: "could"/"may" in place of "would"

Changed as suggested (L460).

7. L329-334 Limitations Section
Comment: Something to add here would be availability of inpatient beds / availability of hospital staff

Thank you for this suggestion. We have added these examples of differences in resources to the Limitations section (L480-481).

8. Figures are a little blurry on paper prior to clicking on access/download link. Is there any way the image can be more clear?

Better quality figures in .tif format have been uploaded. Image files were run through the journal PACE tool to ensure adequate clarity for publication.

9. Overall, very well written paper

Thank you!

Reviewers' comments:

Reviewer's Responses to Questions

Comments to the Author

1. Does this manuscript meet PLOS Mental Health's publication criteria? Is the manuscript technically sound, and do the data support the conclusions? The manuscript must describe methodologically and ethically rigorous research with conclusions that are appropriately drawn based on the data presented.

Reviewer #1: Yes

Reviewer #2: Yes

2. Has the statistical analysis been performed appropriately and rigorously?

Reviewer #1: I don't know

Reviewer #2: Yes

3. Have the authors made all data underlying the findings in their manuscript fully available (please refer to the Data Availability Statement at the start of the manuscript PDF file)?

The PLOS Data policy requires authors to make all data underlying the findings described in their manuscript fully available without restriction, with rare exception. The data should be provided as part of the manuscript or its supporting information, or deposited to a public repository. For example, in addition to summary statistics, the data points behind means, medians and variance measures should be available. If there are restrictions on publicly sharing data—e.g. participant privacy or use of data from a third party—those must be specified.

Reviewer #1: No

Reviewer #2: Yes

4. Is the manuscript presented in an intelligible fashion and written in standard English?

PLOS Mental Health does not copyedit accepted manuscripts, so the language in submitted articles must be clear, correct, and unambiguous. Any typographical or grammatical errors should be corrected at revision, so please note any specific errors here.

Reviewer #1: Yes

Reviewer #2: Yes
